# Vasopressor use after noncardiac surgery: an international observational study

**DOI:** 10.1016/j.bja.2025.07.034

**Published:** 2025-07-11

**Authors:** Ib Jammer, Peter Martin, Hannah Wunsch, Sophie Debouche, Pierre Harlet, Ramani Moonesinghe, Lui Forni, Ben Creagh-Brown, Writing committee for the Squeeze investigators, Meriem Abodun, Souad Bouaoud, Kamel Bouchenak, Hind Saada, Amine Naili, Shruti Chitnis, Marlena Bartmanska, Lip-Yong Choo, Jolene Lim, Estelle Meirau, Rhys Powell, Erica Remedios, Jam Sadullah, Alex Shivarev, Archana Shrivathsa, Marissa Woodburn, Andrew Hughes, Benjamin King-Koi, Anil Mall, Tharindu Vithanage, Baraniselvan Ramalingam, Malcolm Ronald James Bannerman, Clare Margaret Shiner, Trylon Matthew Tsang, David Highton, Steven Ayotte, Allison Kearney, Edward Thornely, Susanna Van Haeringen, Amos Moody, Daniel Kim, Claire Rose, Mahmoud Ugool, Will Zore, Andrew Toner, Patricia Anagnostides, Jodie Jamieson, Hilary Leeson, Susan March, Ronithung Ovung, Alessandra Parini, Toby Shipway, Wai Phen Arthur Teo, Huw Wilkins, Kahina Wotton-Hamrioui, Jodie Jamieson, Sarah Liew, Ashleigh Cargill, Dale Currigan, Edward Gomm, Calvin Lo, Peri Mickle, Marli Smit, Simon Bradbeer, Paul Köglberger, Thomas Geitmann, Laurenz Hell, Johann Knotzer, Dimitar Tonev, Tanislav Ilchev, Dimitrinka Todorova, Karim Ladha, Ciara Hanley, Gabriella Mattina, Janneth Pazmino-Canizares, Bijan Teja, Matteo Parotto, Samareh Ajami, Humara Poonawala, Carlos Jose Perez Rivera, Laura Ramirez, Juan P. Garcia-Mendez, Sharon Idarraga, Ileana Lulic, Gorana Fingler, Jadranka Pavicic Saric, Jakov Jozić, Višnja Nesek Adam, Tatjana Goranović, Marija Josipović, Ida Kožul, Tina Tomić Mahečić, Leonora Bračun, Josip Kovačević, Katarina Lojna, Anton Šarčević, Marko Tripković, Karlo Uroda, Olav Lilleholt Schjørring, Steen Kåre Fagerberg, Birgitte Brandsborg, Zidryne Karaliunaite, Jens Aage Kølsen-Petersen, Christian Melchior Olesen, Mikkel Andreas Strømgaard Andersen, Henrik Wolsted, Aleksander Fjeld Haugstvedt, Stefan Gärtner, Christine Hangaard Hansen, Mirjana Cihoric, Nicolai Bang Foss, Amalie Rosendahl, Laurits S. Kromberg, Marina A. Nielsen, Bjarne O. Nielsen, Morten Vester-Andersen, Rasmus Philip Nielsen, Katrine Maul Andersen, Mark Billmann, Lars H. Lundstrøm, Gine Glargaard, Christine N. Svendsen, Michael Bøndergaard, Jacob Steinmetz, Felicia Dinesen, Liva Thoft Jensen, Lars Simon Rasmussen, L. Andreas H. Burén, Yomna E. Dean, Elsakka Abdelrahman, S. Rozan Samah, Rozza Hebatullah, Sabry Ahmed, Shehata Sameh, Shehata Mostafa, Talat Nesreen, Dina Ramadan, Mohamed Shemies, Yousef Tanas, Ahmed Abbas, Mostafa Abbas, Gena Elassall, Saied Elsawy, Ramy Hassan, Magdy Mahdy, Fatma Monib, Abdelrahman Ramdan, Mahmoud Saad, Khaled Abdelwahab, Ahmed Eid, Omar Hamdy, Eman Mansour, Moataz Maher Emara, Mohamed Bonna, Maiseloon Mogahed, Hamza Asmaa, Salma Elnoamany, Zeinab Ismail, Mohamed Sameh, Eman Ibrahim El-Desoki Mahmoud, Ahmed Hegazi, Ahmed Samy, Aiman Al-Touny, Shimaa Al-Touny, Eman Teema, Edmundo Pereira De Souza Neto, Kevin Arandel, Remi Bouquerel, Benjamin Le Gaillard, Christophe Pelletier, Antoine Strzelecki, Emmanuel Boselli, Nicolas Chardon, Pierre Rodriguez, Guillaume Besch, Julien Villeneuve, Grégoire Wallon, Mathilde Lefevre, Pierre-Grégoire Guinot, Belaid Bouhemad, Maxime Nguyen, Guillaume Raveau, Gilles Lebuffe, Hélène Beloeil, Ludovic Meuret, Bounes Fanny, Nicolas Ducrocq, Philippe Guerci, Fanny Crouton, Stephanie Chevalier, Marc Anger, Marc Danguy Des Deserts, Philippe Aries, Nicolas Herzog, Johan Schmitt, Xavier Tete, Sascha Treskatsch, Golschan Asgarpur, Tobias Schäzl, Theodor Kempe, Philipp Brandhorst, Henriette Hegermann, Oliver Hölsken, Bernadette Kleikamp, Sophie Reimers, Lars Bergmann, Andreas Mania, Christoph Sponholz, Amir Ali Akbari, Moritz Herzberg, Ann-Catrin Paul, Götz Schmidt, Emmanuel Schneck, Christian Koch, Marit Habicher, Michael Sander, Heinrich Klingler, Mareike Diekmann, Sebastian Schmid, Raimund Huf, Benedikt Schick, Julia Wallqvist, Kowark Ana, Linda Grüßer, Rolf Rossaint, Hanna Schröder, Sebastian Ziemann, Daniel Reuter, Annika Haas, Bernd Saugel, Tom Daubenfeld, Moritz Flick, Alina Kröker, Lorenz Rosenau, Christina Vokuhl, Mirja Wegge, Luisa Weskamm, Kassiani Theodoraki, Sofia Apostolidou, George Gkiokas, Konstantinos Stamatis, Chrysoula Stachtari, Meltem Perente, Georgios Pistiolas, Charalampos Martinos, Theodoros Aslanidis, Eirini Sidiropoulou, Anna Efthymiou, Ghrysanthi Sklavou, Nikolaos Barbetakis, Apostolos Gogakos, Achilleas Lazopoulos, Eleni Mavroudi, Dimitrios Paliouras, Thomas Rallis, Evangelia Samara, Ioanna Iatrelli, Eleni Panagiotou, Eumorfia Kondili, Anthoula Ntakoula, Eleftherios Papadakis, Konstantinos Sorokos, Martin I. Sigurdsson, Helgi Egilsson, Piyush Ranjan, Puneet Khanna, Arun Kumar, Ashu Sara Mathai, Gincy Ann Lukachan, Radhika Nair, Kalpana Balakrishnan, Punitha Chockalingam, Shah Bhagyesh, Edward Johnson Joseph, Veena Gopal, Arjun Bhagavath K R, Shivakumar Channabasappa, Pooja Shah, Najah Hadi, Ali Najeh Al-Awwady, Maytham Aqeel Al-Juaifari, Angelo Giacomucci, Francesco Brunelli, Elisa Scarpone, Elena Giovanna Bignami, Valentina Bellini, Andrea Bonetti, Jessica Colla, Savino Spadaro, Giacomo Baldisserotto, Paolo Priani, Margherita Sella, Andrea Russo, Laura Cascarano, Bruno Romanò, Giulia Torregiani, Maurizio Cecconi, Massimiliano Greco, Nicolò Martinetti, Sergio Palma, Andrea Pradella, Rosella Nicoletti, Barbara Bacer, Martina Guarnera, Michela Lotierzo, Sara Miori, Sergio Lassola, Andrea Sanna, Iacopo Cappellini, Filippo Becherucci, Lucia Zamidei, Guglielmo Consales, Lorenzo Tutino, Luigi Vetrugno, Gloria Marson, Gianluca Zani, Giulia Felloni, Maurizio Fusari, Claudio Gecele, Massimo Terenzoni, Andrea Cortegiani, Giulia Catalisano, Tatiana Catania Cucchiara, Dario Calogero Fricano, Giulia Ingoglia, Mariachiara Ippolito, Claudia Marino, Gabriele Presti, Lucia Mirabella, Antonio De Candia, Nicole Pepe, Kiyoyasu Kurahashi, Munehito Uchiyama, Hiroshi Morimatsu, Kosuke Kuroda, Kaori Yamashita, Tatsuya Kida, Tomohide Takei, Sohaib Al-Omary, Lara Alnajjar Lara, Majjd Alnajjar Lara, Amro Abuleil, Antigona Hasani, Marin Almahroush, Marya Bensalem, Mawadaa Alttir, Muhammed Elhadi, Akram Alkseek, Hibah Bileid Bakeer, Eman Abdulwahed, Entisar Alshareea, Reem Ghmagh, Doaa Gidiem, Enas Soula, Mohd Zulfakar Mazlan, Sanihah Che Omar, Mohamad Hasyizan Hassan, Shamsul Kamalrujan Hassan, Huda Zainal Abidin, Ion Chesov, Mihai Tiple, Natalia Zadiraca, Diana Boleac, Doina Oglinda, Abdelghafour El Koundi, Hicham Balkhi, Mustapha Bensghir, Noureddine Kartite, Abdelilah Ghannam, Othman Belarabi, Zakaria Belkhadir, Brahim El Ahmadi, Elisavet Karkala, Maria Christina Ravn, Oda Uhlin Husebekk, Renate Johnsen, Ib Jammer, Vegard Lundevall, Wiszt Radovan, Andreas Haugerud, Ine Karoline Stenersen, Agnete Prydz, David Frederic Knutsen, Heidi Marthea Ohnstad, Roy B. Olsen, Elise Runde Krogstad, Anna Sigurdardottir, Krzych Łukasz, Michal Szewczyk, Cristina Granja, Catarina Dourado, Nidia Gonçalves, Francisco Matias, Ana Raimundo, Rui Pedro Cunha, Miguel Tavares, Alexandre Pinto, Cristina Torrão, Lnês Amaral, Ana Rita Costa, Ricardo Marinho, Miguel Ricardo, César Vidal, Alice Santos, Julia Mendonça, Daniela Xara, Raul Neto, João Tiago Rodrigues, Ricardo Amaral, Diogo Oliveira, José Sampaio, Francisca Cardoso, José Caldeiro, Rogério Corga, Edite Mendes, Pedro Moura, Rita Passos, Francisco Silva, Sofia Trovisco, Inês Fonseca, Décio Pereira, Lina Miranda, Muhammad Shakeel Riaz, Hamed Elgendy, Hashaam Ghafoor, Mohammed Haji, Vipin Kumari, Lakshmi Ramanathan, Jassim Rauf, Nissar Shaikh, Abdul Gafoor Tharayil, Marija Toleska, Aleksandar Dimitrovski, Filip Naumovski, Angela Trposka, Ioana Marina Grintescu, Cristian Cobilinschi, Ana-Maria Cotae, Liliana Mirea, Raluca Ungureanu, Liana Valeanu, Bianca Morosanu, Serban Bubenek-Turtoni, Cornel Robu, Alida Moise, Carmen Balescu, Catalin Traian Guran, Madalina Herman, Alexander Kulikov, Igor Zabolotskikh, Dmitriy Fedunets, Nikita Trembach, Valerii Subbotin, Ilyas Izmailov, Maria Miroshnichenko, Ekaterina Orlova, Elizaveta Serdobintseva, Mikhail Kirov, Aleksey Avidzba, Vsevolod Kuzkov, Anton Nikonov, Sergey Astrakov, Elena Neporada, Victoria Khoronenko, Vladislav Karpeikin, Anna Malanova, Pavel Suvorin, July Zaharenkova, Sergey Efremov, Oleg Kuleshov, Alexey Kulikov, Elizaveta Leonova, Olivera Marinkovic, Ana Sekulic, Ivan Palibrk, Marija Djukanovic, Svetlana Sreckovic, Radmila Klacar, Dragana Vracevic, Miodrag Milenovic, Aleksandra Nikolic, Marija Rajkovic, Dragana Lončar Stojiljković, Nikola Djukanović, Biljana Novaković, Maja Stojanovic, Milan Markovic, Slobodan Popovic, Janez Dolinar, Sandra Blagojević Štembergar, Goran Kurnik, Peter Poredos, Vanja Oven, Andreja Möller Petrun, Bojana Drobnjak, Maša Furman, Marko Lokar, Jernej Novak, Katarina Katja Primožič, Palesa Motshabi Chakane, Sithandiwe Dingezweni, Leballo Gontse, Zainub Jooma, Hlamatsi Moutlana, Lunganga Toms Lushiku, Grace Manjooran, Palesa Mogane, Mathabe Sehlapelo, Sean Chetty, Stephen Venter, Triesie Lotz, Pablo Monedero, Carmen Cara-Gilabert, Angela Escribano-Arranz, Marta Luque-Peláez, Pablo Montero-López, Inigo Iñigo Rubio-Baines, Carmen Sala-Trull, Ana María García Sánchez, Cristina Blanco Dorado, Angela Casquero Murciego, Francisco García Lázaro, Yaiza Molero Diez, F Javier García-Miguel, Estefania Chamorro Garci, Rosalia Navarro-Perez, Luis Santé, Andrea Gutiérrez, Marta Luzón, Rosalba Martinez, Eduardo Passariello, Ana Ruiz, Ferran Serralta, Jaume Valero, Susana Altaba Tena, Maria Lidon Mateu Campos, Luisa Cueva Castro, Albert Bainac Albadalejo, Astrid Batalla Gonzalez, Cecilia Diez García, Marta Giné Servén, Laura Pardo Pinzón, Hector Villanueva Sanchez, Ángel Becerra-Bolaños, Antonio Arencibia-Almeida, Gema Hernanz-Rodríguez, Virginia Muiño-Palomar, Nazario Ojeda-Betancor, Aurelio Rodríguez-Pérez, José Ignacio García-Sánchez, Tamara Brunete, David Delgado, Pablo Redondo, Viviana Varón, Diana Zamudio, Javi Ripolles, Susana González-Suárez, Elena Regla Gómez-González, María Del Carmen Iribarren Mateos, Hytham K.S. Hamid, Alaa Musa, Elfayadh Saidahmed, Ahmed Mohamed Ibrahim Mohamed, Muntasir Abdelsakhi, Walaa Abdelrouf Ibrahim, Abdulrhman Khaity, Michelle Chew, Helen Didriksson, Carina Jonsson, Thorir S. Sigmundsson, Anna Granström, Malin Jonsson Fagerlund, Anna Schening, Arman Valadkhani, Christina Blixt, Malin Hansson, Kristina Kilsand, Åke Norberg, Eva Strandberg, Egidijus Semenas, Lina Jonikaite, Alexander Dullenkopf, Ivan Chau, Lina Petersen, Benedikt Preckel, Ali Kaplan, Jimmy Schenk, Denise Petra Veelo, Felix Van Lier, Rene Van Bruchem, Seppe SHA. Koopman, Toine Van Den Ende, Hans D. De Boer, Henriëtte Smid-Nanninga, Paul A. Van Beest, Eric E.C. De Waal, Thomas W.L. Scheeren, Ilonka N. De Keijzer, Constanze Brucker, Sandra Brookman, Inge J.E. Paas, Lerzan Dogan, Hazal Yazgec, Cigdem Yildirim Guclu, Sanem Cakar Turhan, Basak Ceyda Meco, Ali Alagoz, Hilal Sazak, Arzu Yıldırım Ar, Öznur Demiroluk, Yıldız Yiğit, Osman Ekinci, Serap Adana Kavlak, Seymanur Altintas Filizoglu, Günseli Orhun, Mert Canbaz, Kemal Tolga Saracoglu, Elif Akova Deniz, Banu Eler Cevik, Ayca Sultan Sahin, Ebru Kaya, Hande Gurbuz, Derya Karasu, Seyda Efsun Ozgunay, Eren Fatma Akcil, Ozlem Korkmaz Dilmen, Yusuf Tunali, Kerem Erkalp, Ali Ozalp, Mehmet Salih Sevdi, Maryna Freigofer, Olena Khomenko, Pavlo Hurin, Dmytro Dmytriiev, Eugenii Lysak, Tamsin Gregory, Shaw Alison, Ratcliffe Anita, Hairsine Brigid, Adam Farrar, Samson A. Williams, Joyce Yeung, Syed Abid, Adetoro Akintunde, Roshni Bahri, Marta Burak, Libby Dias, Yash Dinesh, Iman Farah, Ciara Gibson, Joanne Gresty, Fiona Harris, Alex Jones, Chuck Lam, William Mciver, Teresa Melody, Ninoshka Merchant, Safwaan Patel, Gursharan Virdee, Bryan Wong, Jessica Davis, Jordan Alfonso, Mohamed Elbahnasy, Monica Trivedi, Efthymia Maria Kapasouri, Galina Maneva, Peta Masters, Malgorzata Opalinska, Luke Winslow, Bamford Peter, Prince Judith, Faulkner Maria, Ivison Alison, Barham Elin, Barton Matthew, Hadlett Max, Russell Nicki, Verghese Prashant, Karunaratne Nicholas, Murphy Thomas, Sundar Ashok, Christopher Black, Zakaulla Belagodu, Ryan Coe, Katy Collins, Tracy Edmunds, Charlotte Kamundi, Prasanna Patlola, Laura Johnson, Naomi Oakley, Olumide Olufuwa, Luciana Rusu, Juleen Fasham, Amit Das, Anna Ratcliffe, Ben Parish, Freeman Lizzie, Gary Minto, Gunarathna Perumbadage, Jessica Sinclair, Lucy Guile, Matthew Baldwin, Stephanie Pauling, Wael Alhalabi, Moustafa Shebl Zahra, Eva Beranova, Tracy Hazelton, Alicia Knight, Trudy Parfrey, Jhanielle Quindoyos, Hazel Ramos, Gabriella Tutt, Joanne Deery, Himanshu Arora, David Freeman, Qasim Tayyib Ahmed, Alexander Gurnee, Rachel Harding, Tom Mckernan, Aayesha Kazi, Nicholas Truman, Stephen Lewis, Eid Ahmed, Baiju Barath, Bernardo Solomon, Hau Lam Clara Fong, Stevenson Joe, Katarzyna Anna Marasinska, Abelarde Kaye, Essuman Lorinda, Whitmarsh Thomas, Jack Tooze, Bland Yvonne, Andrew Lowes, Mohamed Abdelsalam, Jon Braviner, Rachael Lucas, Jenny Ritzema, Nicolas Simmers, Manushi Vyas, Venkat Sundaram, Anette Bolger, Jennifer Davies, Esther Garrod, Victoria Garvey, Rachel Manley, Zuzana Probier, Angela Pye, Zoka Milan, Gudrun Kunst, Daveena Meeks, Anna Broderick, Kevin O'Reilly, Juliana Pereira, Bernd Oliver Rose, Leanne Howard, Estefania Treus, Teodora Orasanu, Russell Conyers, Katie Dorr, Ellie Farcas, Olesya Francis, Kelly Hubbard, Rachel Newton, Sarah Shephardson, Catherine Wyatt, David Golden, Amy Ackerley, Laura Adams, Jennifer Assimakopoulos, Miriam Davey, Maddie Lawrence, Rebecca Seaman, Michala Shah, Heather Callaghan, Kailash Bhatia, Mohamed Abdelmotieleb, Victor Bill, Ayman Edarous, Samuel Ikenga, Rose Jama, Kezia Philipose, Manu Sudevan, Brendan Sloan, Sarah Buckley, Anna Littlejohns, Amy Major, Lauren Tye, Manoj Wickramasinghe, Katie Wilson, Richard Stewart, Teena Babu, Louise Mew, Alistair Sawyerr, Sharon Baxter-Dore, Nowfal Rahman, Joanne Rothwell, Helen T-Michael, Shiny Sivanandan, Kirsty Allen, Daniele Arcoria, Roberta De Pretto, Gbemisola Jenfa, Zoey Horne, Zainab Mavani, Graeme Mclintock, Emmah Nelly, Ionela Sinanovic, Natalie Temple, Josephine Williams, Anand Jayaraman, Joshua Craig, Hayley Mckie, Tracy Smith, Gail Waddell, Trish Tsuro, Khaled Ahmed, Alya Amin, Kimberley Netherton, Izuchukwu Nwalusi, Bryony Saint, Kinga Szymiczek, Reschreiter Henrik, Leanne Bartlett, Yasmin De'Ath, Charlotte Humphrey, Emma Langridge, Rebecca Miln, Tomasz Torlinski, Tony Whitehouse, Ian Ewington, Phillip Howells, Randeep Mullhi, Amit Sharma, Hazel Smith, Carla Speziale, Julian Giles, Joel Lockwood, Henrik Reschreiter, Chloe Bascombe, Claire Osey, Debbie Branney, Tiller Heather, Javen Ramsami, Sally Pitts, Annamaria Wilce, Natalie Agius, Lindsay Rogers, Cheryl Lindsay, Claire Preedy, Luke Hayward, Thomas Clark, Kevin Windsor, Kizzy Baines, Ben Dingle, Rebecca Wilcock, Hemal Bosamia, Toby Lewis, Ingeborg Welters, Richard Ramsaran, Aleem Morenikeji, Annie Smith, Maria Arra Carlota Canada, Claire Davies, Dan Watkin, Jon Machin, Katherine Hodson, Maria Lopez, Luke Shearer, Nick Sinanan, Maria Norris, Rebecca Vickers, David Shaw, Victoria Waugh, Karen Williams, Hayaka Amada, Alexander Sell, Tuyen Anthony, Anamaria Gerea, Shamil Tanna, Angus Tulloch, Panagiota Alexopoulou, Naomi Boyer, Paula Carvelli, Benedikt Creagh-Brown, Olivia Dow, Fouad El-Hibri, Syeda Haider, James Hilton, Hannah Mackay, Wisdom Mbama, Natalia Michalak, Maskell Nick, Kanji Rafiq, Donna-May Sanga, Nasser Syed, Jerik Verula, Waldtraud Wutte, Pierson Richard, Butler Jack, Elena Anastasescu, Tania Mellor, Wallbridge Thomas, Emma Ingall, Kim Jemmett, Vicki Priestly, James Rand, Maxime Rigaudy, Reanne Solly, Sarah Stirrup, Heather Weston, Wayne Evans, Chloe Perry, Karen Pilson, Peringathara Biju, Jones Claudette, Keeling Ellie, Jones James, Brodie Teresa, Magaya Valarie, Andrew Woodgate, Kylie Ashby, Pauline Aspa, Kelly Barrett, Lauren Blunt, Sean Caunter, Emily Flavell, Peter Fletcher, Angie Foulds, Ashleigh Fynn, Beth Mcelroy, Bryony Reed, Fleur Rogers, Andrea Ford, Emma Bartlett, David Golden, Amy Ackerley, Laura Adams, Jennifer Assimakopoulos, Miriam Davey, Madeleine Lawrence, Rebecca Seaman, Michala Shah, Heather Callaghan, Bernd Oliver Rose, Jacob Burr, Rosie Reece-Anthony, Georgia Richmond, Kay Spikes, Eleanor Stranger, Danaja Zolger, Vera Gotz, Ben Wooldridge, Bridget Campbell, Penny Parsons, Camilla Stagg, Dominika Dabrowska, Omnia Askar, Priyakam Chowdhury, Jamie Gonzales, Swarna Jeyabraba, Angelyn Sangalang, Amrinder Sayan, Surendini Thayaparan, Kaushik Bhowmick, Sally Humphreys, Nimal Mani, Sarah Pearcey, Shivacharan Patel Rudrappa, Zi Yi Tew, Lisa Jobes, John Harris, Rachel Hughes, Emma Mcivor, Rebecca Pope, Mary Roberts, Victoria Whitehead, Peter Alexander, Sheetal Crasta, Sofia Fiouni, Jane Shaw, Luke Ward, Simon Davies, Harriet Carter, Zoe Scott, Anisha Rahmath Varodan, Liudmila Asaul, Konstantin Balonov, Ana Arias, Leidy Rivas, Julio Pineda, Neal Fleming, Brittney Saverimuttu, Aubrey Yao, Meredith Miller, Anuradha Borle, Omokhaye Higo, Muthuraj Kanakaraj, Sylvia Daamen, Sophie Debouche, Slama Farsi, Pierre Harlet, Flavia Pirovano

**Affiliations:** 1 Department of Anaesthesia and Intensive Care, Haukeland University Hospital, Bergen, Norway; 2 Institute of Epidemiology and Health Care, University College London, London, UK; 3 Department of Anesthesiology, Weill Cornell Medicine, New York, NY, USA; 4 Department of Anesthesiology and Pain Medicine, University of Toronto, Toronto, ON, Canada; 5 European Society of Anaesthesiology and Intensive Care, Brussels, Belgium; 6 Centre for Perioperative Medicine, University College London, London, UK; 7 Intensive Care Unit, Royal Surrey County Hospital, Guildford, UK; 8 School of Medicine, University of Surrey, Guildford, UK

**Keywords:** haemodynamic, noradrenaline, perioperative medicine, postoperative hypotension, vasoconstrictors, vasoplegia, vasopressors

## Abstract

**Background:**

Hypotension after major noncardiac surgery is associated with increased morbidity, mortality, and costs, and is often treated with postoperative vasopressor infusions. The frequency of administration in the postoperative period is unknown.

**Methods:**

This international prospective cohort study was conducted between October 2020 and October 2023. At each hospital, adults undergoing noncardiac surgery were enrolled into two cohorts: all consecutive patients for 1 week (Cohort A) and an additional sample of up to 30 consecutive patients administered postoperative vasopressor infusions within 1 yr (Cohort B). The primary outcome (Cohort A) was the incidence of postoperative vasopressor infusions, defined as any continuous infusion of vasopressors. Secondary outcomes included in-hospital mortality, organ dysfunction, length of hospital stay, and complications associated with postoperative vasopressor infusions (both cohorts).

**Results:**

In total, 25 675 participants were enrolled from 228 hospitals across 42 countries. In Cohort A, 770/19 768 (3.9%) participants received postoperative vasopressor infusions, with vasopressor use ranging between 0% and 18% across hospitals (median odds ratio: 2.30 [credible interval 1.96–2.73]). This variability did not alter after adjustment for case-mix and procedural characteristics. For both cohorts, postoperative vasopressor infusions were associated with higher (15.5%) in-hospital mortality, higher rates of organ failure, and longer hospital stay.

**Conclusions:**

Administration of postoperative vasopressors after noncardiac surgery varied across hospitals and was associated with worse outcomes. Variable practice across hospitals could not be explained by differences in case-mix.

**Clinical trial registration:**

https://clinicaltrials.gov/study/NCT03805230, ESAIC tracking ID: ESAIC_CTN_SQUEEZE.

**Editor’s key points**

• The frequency of hypotension after major noncardiac surgery requiring postoperative vasopressor support is unknown.
• This international prospective cohort study established the incidence of postoperative vasopressor infusions.
• Of 19 768 participants, 770 (3.9%) received postoperative vasopressor infusions, but there was wide variability between hospitals.
• Postoperative vasopressor use was associated with higher rates of organ failure, complications, and death.
• Variable practice across hospitals could not be explained by differences in case-mix.

Hypotension after surgery is common and associated with increased morbidity and mortality.^1^ Hypotension may be mediated by a decrease in preload, afterload, or both, or by impaired cardiac contractility with concomitant vasodilation.^2^ Common causes of hypotension may include preoperative and intraoperative medications, neuraxial anaesthesia, or systemic inflammation.^3^ Postoperative vasopressor infusions are used if the clinician perceives that vasodilation is significantly contributing to hypotension.

Postoperative hypotension may be prolonged, is often unrecognised, and might be more relevant to patient outcome than intraoperative hypotension.^4^ Although there are many studies of intraoperative hypotension,^4^ there are no large epidemiological studies on the use of postoperative vasopressor infusions after major noncardiac surgery. A previous estimate of the incidence of postoperative vasopressor infusions was 2%, although this was based on highly uncertain evidence.^5^^,^^6^ Furthermore, although there appears to be wide practice variability in the use of postoperative vasopressor infusions,^7^ there remains a lack of high-quality epidemiological data in unselected populations.^8^

Given the limited data on this topic, our goal was to describe the epidemiology of postoperative vasopressor infusions, to explore variation in practice, and identify areas of uncertainty for future research and guidelines. Our primary aim was to estimate the proportion of noncardiac surgery patients who received postoperative vasopressor infusions and document the variability of vasopressor use between hospitals. Our secondary aims were to record patient and perioperative characteristics, in addition to adverse outcomes, associated with postoperative vasopressor infusions.

## Methods

### Study design

The Squeeze study was an international, multicentre, prospective, observational cohort study of postoperative vasopressor use. The recruitment of participating hospitals was directed by national coordinators, intensive care societies (European Society of Anaesthesiology and Intensive Care, European Society of Intensive Care Medicine) and through direct contact by the steering committee members (Supplementary material). The study was approved by national and local ethics committees and performed in accordance with the Declaration of Helsinki. Consent procedures were guided by national regulations and either consisted of informed consent (79 hospitals, 35%) or a waiver of consent (149 hospitals, 65%). The protocol and statistical analysis plan have been previously published,^9^ and this study is reported in accordance with the STROBE guidelines.^10^

### Study protocol

Participants were enrolled into two cohorts: Cohort A included all adult patients admitted over 7 consecutive days who met the inclusion criteria (Supplementary Methods 1), intending to represent all major surgeries that occurred in hospitals excluding cardiac, transplant, obstetric, and day case operations. To increase the size of the sample of those who received postoperative vasopressor infusions, each hospital collected Cohort B, which included up to 30 additional consecutive participants who met the same inclusion criteria and received postoperative vasopressor infusions (Supplementary Methods 2). For the purpose of this study, postoperative vasopressor infusion was defined as the postoperative continuous i.v. infusion of a drug with a predominant vasoconstrictor effect (vasopressor). The definition excluded patients receiving intraoperative vasopressor infusions that ended within 1 h of surgery and those starting vasopressors more than 24 h post-surgery (Supplementary Methods 3).

### Data collection

Data were collected at participating hospitals between October 2020 and October 2023, with each site choosing their own start date within that window. Information on each patient included data on the entire hospital stay from surgery to discharge or death. Core data included patients’ health status, regular medications, preoperative vital signs, type of surgery, anaesthetic details, and postoperative complications and outcomes. For patients receiving a postoperative vasopressor infusion, additional data were extracted from the chart on assessments before starting vasopressor support, blood pressure targets, vasopressor use, organ support duration, and SARS-CoV-2 status. Data confidentiality was maintained by using a secure management system (OpenClinica v. 3.17. Copyright; OpenClinica LLC and collaborators, Waltham, MA, USA). Hospitals collected data from the medical record and entered it into an electronic case report form (Supplementary Methods 4) managed by the European Society of Anaesthesiology and Intensive Care research office, which mandated key information entry and included error checking. A manual of procedures that included definitions of all variables was provided to investigators (Supplementary Methods 5). Data quality and completeness were continuously monitored (Supplementary Methods 6).

### Primary outcome

The primary outcome was the incidence of postoperative vasopressor use in Cohort A.

### Secondary outcomes

Secondary outcomes were assessed in both cohorts including mortality (death before discharge, censored at day 30), organ dysfunction (Supplementary Methods), and length of hospital stay.

### Statistical analyses

The variation of postoperative vasopressor infusions across hospitals and countries was modelled using Bayesian mixed-effects logistic regression with random intercepts for hospitals and countries (Model 1). Modelled estimates provide a more stable, reliable, and interpretable view of the data by reducing noise, accounting for regression to the mean, and incorporating uncertainty. Noninformative or weakly informative priors were used throughout. Estimates of the random effect variances were transformed into median odds ratios.^11^ To account for regression to the mean, hospital- and country-specific estimates of rates of postoperative vasopressor infusion use were derived using best linear unbiased estimators. For exploratory purposes, countries were also identified by income status extracted from the 2023 World Bank Classification System.^12^

To determine whether characteristics of the patient and surgical procedure explained the between-hospital and between-country variation, three models were examined (Supplementary Methods including Supplementary Table 1). To explore participant outcomes associated with postoperative vasopressor infusion use, data from Cohorts A and B were combined. To gauge potential selection bias associated with enrolment in Cohort B, participants in Cohort B (all postoperative vasopressor infusion recipients) were compared with participants receiving postoperative vasopressor infusions in Cohort A (Supplementary Table 7). Associations between postoperative vasopressor infusion use and outcomes in all participants from both cohorts were modelled using Bayesian multilevel logistic regression, with random intercepts for hospital and country and adjusting for preoperative variables. Bayesian multilevel quantile regression was used to model length of stay. A binary indicator for Cohort (A or B) was added to these models to adjust for any residual selection bias.

To explore the relationship between exposure to vasopressors (both during and after surgery), participants from both cohorts were divided into five group: (1) no vasopressors, (2) intraoperative vasopressors only (bolus or infusion), (3) postoperative boluses and enteral vasopressors (but no infusions), (4) short-term postoperative vasopressor infusion (1–2 days after surgery), (5) and prolonged postoperative vasopressor infusion (≥3 days). The latter three groups included participants who may also have received intraoperative vasopressors. Percentages of adverse outcomes were descriptively compared, as was the distribution of length of stay, across these five groups. All analyses were conducted using R software, version 4.4.1 (R Foundation for Statistical Computing, Vienna, Austria).^13^^,^^14^

## Results

### Participants

Between October 2020 and October 2023, 25 675 participants were recruited from 228 hospitals in 42 countries, with 19 768 recruited in Cohort A (Table 1; Supplementary Tables 1 and 2).

**Table 1:** Baseline variables. Preoperative and perioperative characteristics of patients by study cohort and postoperative vasopressor infusion use (in Cohort A). Sample sizes vary by variable as a result of missing values. All percentages are reported as column percentages. IQR, interquartile range. COPD, chronic obstructive pulmonary disease.

Variable	Cohort A (*N*=19 768)						Cohort B (*N*=5907)	
	Total, *n*	%	No postoperative vasopressor infusion, *n*	%	Postoperative vasopressor infusion, *n*	%	Total, *n*	%
Age (yr)								
<50	6281	31.8	6175	32.5	106	13.8	861	14.6
50–69	7455	37.7	7131	37.5	324	42.1	2286	38.7
≥70	6032	30.5	5692	30.0	340	44.2	2760	46.7
Sex								
Female	9730	49.4	9428	49.8	302	39.3	2461	41.9
Male	9975	50.6	9508	50.2	467	60.7	3406	58.1
Medical history								
Coronary artery	1878	9.5	1727	9.1	151	19.6	1037	17.6
Cerebrovascular	1335	6.8	1225	6.4	110	19.6	592	10.0
Peripheral vascular	1325	6.7	1223	6.4	102	14.3	738	12.5
Arterial fibrillation	1387	7.0	1270	6.7	117	13.2	867	14.7
Heart failure	1069	5.4	986	5.2	83	15.2	707	12.0
Hypertension	7881	39.9	7468	39.3	413	10.8	3097	52.5
Diabetes mellitus								
Insulin dependent	990	5.0	934	4.9	56	7.3	503	8.5
Non-insulin dependent	2152	10.9	2034	10.7	118	15.3	909	15.4
Chronic liver	520	2.6	475	2.5	45	5.8	325	5.5
Chronic respiratory								
COPD	1243	6.3	1152	6.1	91	11.8	745	12.6
Other	1371	6.9	1312	6.9	59	7.7	432	7.3
Steroids	692	3.5	657	3.5	35	4.5	273	4.6
Any antihypertensive	7553	38.2	7131	37.5	422	54.8	3127	52.9
ASA physical status								
1	3720	18.9	3692	19.5	28	3.6	262	4.4
2	9064	45.9	8852	46.7	212	27.6	1424	24.1
3	5994	30.4	5632	29.7	362	47.1	2632	44.6
4	900	4.6	757	4.0	143	18.6	1393	23.6
5	53	0.3	30	0.2	23	3.0	190	3.2
Surgical procedure								
Breast	598	3.0	594	3.1	4	0.5	21	0.4
Gynaecological	1383	7.0	1354	7.1	29	3.8	196	3.3
Head and neck	1920	9.7	1870	9.8	50	6.5	246	4.2
Hepatobiliary	979	5.0	920	4.8	59	7.7	457	7.7
Kidney/urological	2194	11.1	2129	11.2	65	8.4	436	7.4
Lower gastrointestinal	2781	14.1	2610	13.7	171	22.2	1853	31.4
Orthopaedic	4953	25.1	4853	25.5	100	13.0	711	12.0
Plastic/cutaneous	902	4.6	886	4.7	16	2.1	120	2.0
Upper gastrointestinal	1271	6.4	1172	6.2	99	12.9	729	12.3
Neurological/spinal	1154	5.8	1093	5.8	61	7.9	281	4.8
Vascular	920	4.7	830	4.4	90	11.7	592	10.0
Other	711	3.6	685	3.6	26	3.4	264	4.5
Severity								
Minor	2553	12.9	2536	13.4	17	2.2	102	1.7
Intermediate	9857	49.9	9678	51.0	179	23.2	938	15.9
Major	7354	37.2	6780	35.7	574	74.5	4866	82.4
Urgency								
Not urgent	13 306	67.4	12 917	68.0	389	50.6	2380	40.3
Urgent	6449	32.6	6069	32.0	380	49.4	3524	59.7
Airway								
Tracheal tube	12 980	65.9	12 273	64.8	707	91.9	5415	91.9
Supraglottic	2566	13.0	2553	13.5	13	1.7	94	1.6
O_2_ facemask or nasal cannula	4156	21.1	4107	21.7	49	6.4	386	6.5
Blood loss (ml)								
<250	15 794	80.6	15 434	81.9	360	47.4	2749	47.2
251–1000	3359	17.1	3092	16.4	267	35.2	2024	34.7
1001–3000	402	2.1	294	1.6	108	14.2	828	14.2
>3000	43	0.2	19	0.1	24	3.2	224	3.8
Duration of operation (min)								
<120	6955	35.3	6869	36.3	86	11.2	628	10.7
120–239	7985	40.6	7786	41.1	199	26.0	1706	29.1
≥240	4748	24.1	4268	22.6	480	62.7	3522	60.1

### Primary outcome: postoperative use of vasopressors

**Fig 1:**
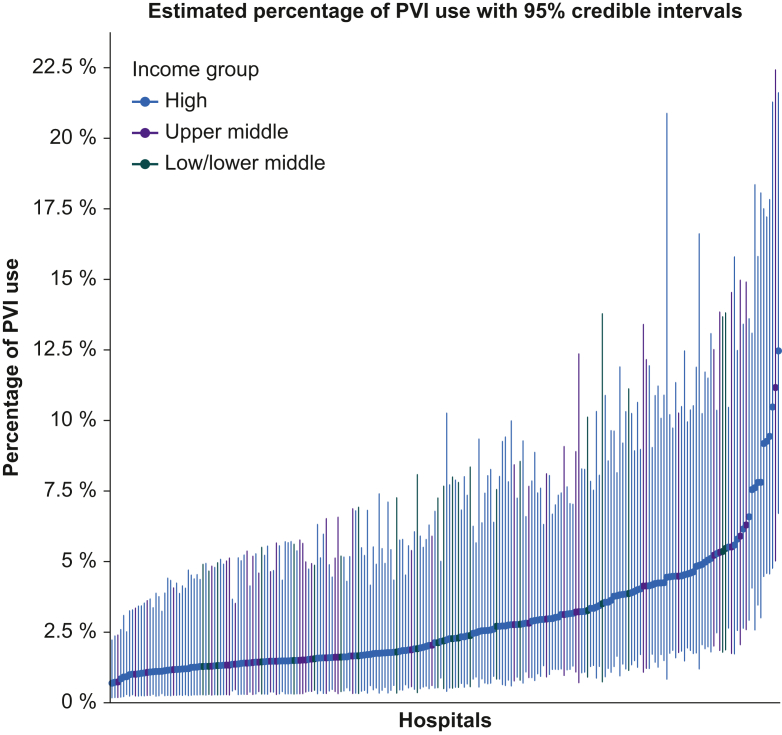
Model of postoperative vasopressor infusion (PVI) use without adjustment for case-mix. Shown is the estimated percentage of PVI use in 228 participating hospitals with 95% credible intervals. The three different colours indicate the income group of each country where the hospital was located, defined by the World Bank (2023).

Of the 19 768 participants in Cohort A, 770 (3.9%) received a postoperative vasopressor infusion (Supplementary Table 3). Substantial variation in the use of postoperative vasopressor infusion was found between hospitals and countries, ranging 0.7–12.5% across hospitals (Fig. 1; Supplementary Figs 1–3).

### Secondary outcomes

#### Patient characteristics associated with postoperative vasopressor infusion use

Using our unadjusted model, the estimated median odds ratio for the between-hospital variation was 2.30 (95% credible interval [CrI]: 1.96–2.73), which remained similar after adjustment for case-mix and procedural characteristics (2.30 [95% CrI: 1.91–2.85]). Results were similar for the between-country variation (Supplementary Table 4). Higher ASA grade, severity and urgency of surgery, and lower preoperative mean arterial pressure were associated with exposure to postoperative vasopressor infusions (Supplementary Table 5). Participants who received upper and lower gastrointestinal, and vascular surgery had higher rates of postoperative vasopressor infusion, compared with participants receiving other types of procedures. No specific aspect of previous medical history was associated with postoperative vasopressor infusion use.

Including both preoperative and intraoperative variables, four characteristics were associated with higher rates of postoperative vasopressor infusion: duration of surgery longer than 4 h, larger volume of blood loss (increasing odds ratio with increasing volume of blood loss), use of vasoactive drugs before or during surgery, and amount of i.v. fluid used (any colloid, any blood transfusion, if crystalloid >1.5 L). Epidural analgesia was also associated with the highest rate of postoperative vasopressor infusion use (Supplementary Table 6).

#### Outcomes associated with postoperative vasopressor infusion use

**Fig 2:**
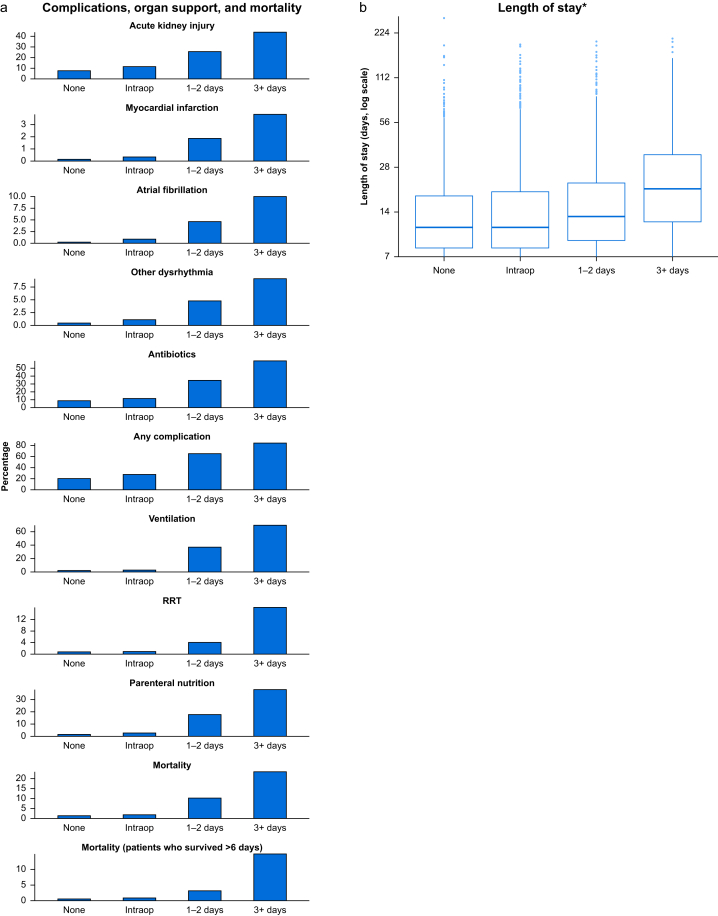
(a) Comparison of clinical complications, antibiotics, and mortality between patients grouped by receipt of vasopressors. (b) Comparison of length of stay between patients grouped by receipt of vasopressors: none (*N*=9570), intraoperative only (*N*=8867), postoperative vasopressor infusion (PVI) with 1–2 days (*N*=3992), prolonged PVI with >2 days (*N*=2685). Actual sample sizes differ by outcome as a result of missing outcome values (see Supplementary Table 1h for details). Note that 561 patients who received postoperative boluses/enteral vasopressors, but no PVI, are excluded from this figure (owing to small sample size in this category). ∗Length of stay: patients who were discharged before day 7 are excluded. Intraop, intraoperative; RRT, renal replacement therapy. Full results are displayed in Supplementary Table 9.

The 30-day in-hospital mortality rate in Cohort A was 2.1%. Participants who received postoperative vasopressor infusions had mortality rates of 12.5% in Cohort A, 15.9% in Cohort B, and 15.5% overall (Supplementary Table 7). From both cohorts (*n*=25 675), we found an association between postoperative vasopressor infusions with adverse outcomes and length of stay, after adjusting for preoperative covariates (Table 2; Supplementary Table 8). Increased exposure to vasopressors over the perioperative period was associated with higher rates of adverse outcomes (Fig. 2a) and longer hospital stay (Fig. 2b; Supplementary Table 9).

**Table 2:** Association between postoperative vasopressor infusion use and patient outcomes. Estimates of the association between postoperative vasopressor infusion use and patient outcomes are presented, adjusted for country, hospital, and preoperative case-mix. Estimates from mixed-effects logistic regression or mixed-effects quantile regression (length of stay). Models included patients from Cohorts A and B, and are adjusted for selection bias into Cohort B. Complete cases analysis (no imputation of missing values). Sample sizes differ as a result of missing outcome values. Adj. OR, adjusted odds ratio; CrI, credible interval. In-hospital mortality is censored at 30 days. Adj OR, adjusted odds ratio for (postoperative vasopressor infusion/no postoperative vasopressor infusion). Adjusted. Median difference: adjusted median difference between length of stay for postoperative vasopressor infusion *vs* no postoperative vasopressor infusion.

Outcome	Cohort A: total (*N*=19 768)		Cohort A: no postoperative vasopressor infusion (*N*=18 998)		Cohort A: postoperative vasopressor infusion (*N*=770)		Cohort B (*N*=5907)		Estimates from Bayesian regression models		
	*n*	%	*n*	%	*n*	%	*n*	%	N∗	Adjusted OR	95% CrI
Ventilation	822	4.2	527	2.8	295	38.3	3032	51.4	18257	24.42	18.38–32.49
Myocardial infarction	63	0.3	50	0.3	13	1.7	165	2.8	18256	3.92	1.68–8.63
Atrial fibrillation	156	0.8	119	0.6	37	4.8	414	7.0	18255	3.91	2.32–6.43
Other dysrhythmia	207	1.1	154	0.8	53	6.9	380	6.4	18255	4.87	3.17–7.45
Renal replacement therapy	208	1.1	163	0.9	45	5.8	546	9.2	18256	3.10	1.89–4.96
Parenteral nutrition	619	3.1	445	2.4	174	22.6	1554	26.3	18255	5.42	4.07–7.21
Antibiotics	2245	11.5	1954	10.4	291	38.4	2649	45.2	18173	3.40	2.74–4.20
Any complications	5245	26.6	4695	24.8	550	71.4	4320	73.3	18244	5.23	4.21–6.53
Acute kidney injury	988	11.3	815	10.1	173	28.6	1650	33.6	10247	2.78	2.16–3.58
30-day mortality	410	2.1	314	1.7	96	12.5	929	15.9	18197	3.82	2.68–5.42

	**Median**	**IQR**	**Median**	**IQR**	**Median**	**IQR**	**Median**	**IQR**	**N∗**	**Adjusted median difference**	**95% CrI**

Length of stay (days)	3	1–6	3	1–6	10	6–20	12	7–23	17169	4.453	3.829–5.090

**Fig 3:**
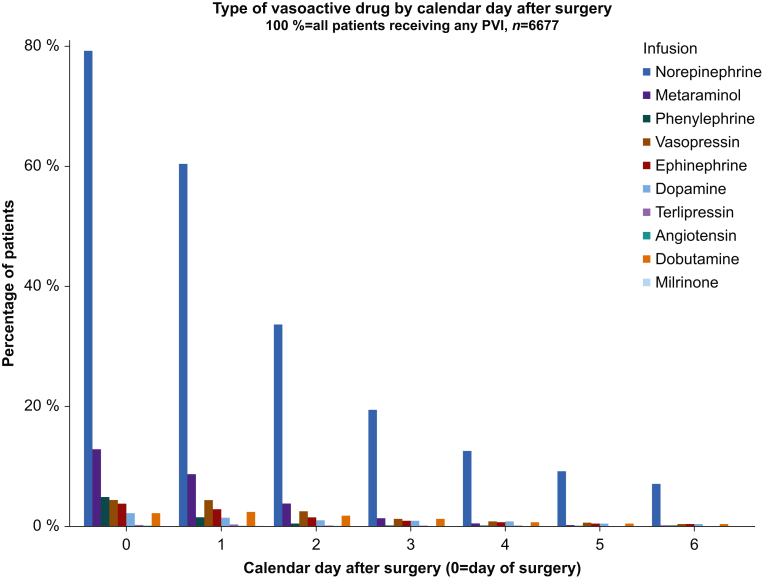
Type of vasoactive infusion (vasopressor or inodilator) given by day post-surgery (*n*=6677).

Norepinephrine was the most common vasopressor used (Fig. 3; Supplementary Table 10). Clinical evaluation alone was the most frequent assessment method before initiation of postoperative vasopressor infusion. Use of cardiac output monitors, echocardiography, or both to guide treatment were rarely reported (Supplementary Table 11). Blood pressure targets were recorded for 60% of patients who received postoperative vasopressor infusions on the day of their operation, with 52–59% on subsequent postoperative days. Among these participants, a higher mean arterial blood pressure target (>65 mm Hg) was associated with the prolonged use of postoperative vasopressor infusions (Supplementary Table 12).

## Discussion

In this prospective international study of patients who underwent noncardiac surgery, approximately 4% of the patients received postoperative vasopressor infusions. We found a considerable variation between hospitals and similar variation between countries, but this was not explained by differences in case-mix. We also found strong associations between postoperative vasopressor infusions and postoperative complications, length of stay, and mortality. The use of postoperative vasopressor infusions is common after surgery—a particularly significant finding given that surgical procedures are among the most frequently performed medical interventions globally, with >300 million operations conducted annually^15^—and interventions designed to optimise postoperative vasopressor infusion use could improve consistency of care and could have large consequences for postoperative outcomes and resource use.

Our findings are consistent with recently reported variability in the use of vasopressors after cardiac surgery, with the admitting hospital a greater determinant than patient characteristics.^16^ However, although postoperative vasoplegia after cardiac surgery is a well-described phenomena, postoperative hypotension after noncardiac surgery is less discussed or explored. Persistent postoperative hypotension despite volume optimisation is a form of shock that has no specific descriptor. Although infection may contribute in some cases, leading, specifically, to septic shock, 41% of patients who received prolonged vasopressor infusion (≥3 days) did not receive antibiotics, indicating that clinicians did not consider infection relevant. Factors associated with increased vasopressor use likely fall into three main categories: (1) tissue injury and inflammatory response (longer surgery, greater blood loss); (2) predisposition owing to illness severity or poor physiological reserve (urgent surgery, impaired physical status); and (3) vasodilatory interventions (epidural analgesia).

We demonstrated that postoperative vasopressor infusion is strongly associated with complications and poor outcomes after surgery. Our data cannot establish causation, and although causal inference methods could help, the relationship is likely more complex than any direct cause-and-effect, involving patient responses to surgical/anaesthetic interventions, resulting pathophysiological changes, and clinical management decisions. Vasopressors are just one potential mediator of harm, and confounding by indication may occur when complications necessitate both postoperative vasopressor infusion and lead to adverse outcomes. The key question remains whether alternative hypotension management approaches could improve patient outcomes.

Noradrenaline was the most common vasopressor, consistent with the literature.^8^ Reported use of objective measures (cardiac output monitoring or echocardiography) to guide the use of vasopressors was rare, suggesting a disconnect between the reality of clinical practice and common expert recommendations. Hypotension causes harm,^1^ but optimal management remains uncertain with contradictory evidence regarding fluid strategies (restrictive *vs* liberal),^17^, ^18^, ^19^ potential harm from inodilator-based fluid optimisation,^20^^,^^21^ and possible vasopressor toxicity.^22^, ^23^, ^24^ Vasopressor selection may be crucial; during a norepinephrine shortage, alternative vasopressor (phenylephrine) use in patients with septic shock was associated with a 3.4% absolute mortality increase.^25^ Potential interventions for perioperative optimisation have been well studied,^21^ but postoperative hypotension management after noncardiac surgery lacks rigorous trials.

The strengths of our study include the large, prospectively acquired sample size, diverse surgical procedures, and participation from 42 countries across different economic settings. The research team maintained data quality through well-designed electronic case reports and central team oversight with error checking. We minimised selection bias by recruiting consecutive patients (1 week for Cohort A, 1 yr for Cohort B) and using consent waivers where possible (65% of hospitals). A published statistical analysis plan prevented selective reporting.^9^ The limitations of the study largely stem from its observational nature, limiting the conclusions that can be drawn, particularly around causation, and its pragmatic, budget-constrained international design. Research duties relied on uncompensated national coordinators and local teams during a pandemic.

Our parsimonious dataset with minimal mandatory fields created information gaps in areas such as blood pressure targets. Cohort B patients appeared slightly sicker than Cohort A patients, with higher mortality, suggesting potential recruitment bias. Although we provided definitions of outcomes such as organ failure, we were unable to strictly adjudicate these data. Reporting of some of these outcomes may have varied witch may have introduced assessment bias. The COVID pandemic unavoidably affected recruitment and case-mix by first limiting, and then increasing, elective surgeries. Finally, we cannot confirm if participating hospitals were representative of their countries’ practices. Sites were self-selected and this could plausibly have introduced bias with patient cohorts not being representative of the broader population, similarly patient selection may not have been truly unbiased. The small sample sizes from many countries limit conclusions about nationwide practice patterns.

The current postoperative hypotension treatment paradigms lack evidence. Our data demonstrating the variability in use of postoperative vasopressor infusions can be used as an impetus for robust intervention studies evaluating different approaches. However, future studies should anticipate heterogeneity of treatment effects across different causes of postoperative hypotension. Failing to account for this heterogeneity may lead to inappropriate trial conclusions that obscure benefits in specific patient subgroups. Options for studies include different approaches to ensuring euvolaemia before starting vasopressors (potentially including cardiac output monitoring for stroke volume optimisation), vasopressor choices such as a comparison of catecholamine vasopressors *vs* non-catecholamine vasopressors, the use of predictive enrichment potentially guided by point-of-care biomarkers such as renin,^26^ and different blood pressure/flow targets. Haemodynamic optimisation strategies that span both intraoperative and postoperative periods could be evaluated as comparisons within a perioperative platform trial. Most recent sepsis research priorities^27^ apply equally to the area of postoperative hypotension management.

In summary, this large global study of vasopressor use in the postoperative period found notable differences in use across hospitals and countries. The use of postoperative vasopressor infusions was strongly associated with complications and poorer outcomes.

## Funding

European Society of Anaesthesiology and Intensive Care (ESAIC). The society provided the trial network and the infrastructure for data collection and data maintenance.

## Authors’ contributions

Conceptualisation: IJ, BCB, HW, LF, RM

Data curation: IJ, BCB, PH, SD, PM

Funding acquisition: IJ, BCB, HW, LF, RM

Investigation: IJ, BCB, PH, SD, HW, LF, RM, PM

Methodology: IJ, BCB, HW, LF, RM, PM

Project administration: IJ, BCB, PH, SD

Software: PH, SD

Statistical analysis: PM

Validation: IJ, BCB, HW, LF, RM, PM

Visualisation: IJ, BCB, HW, LF, RM, PM

Writing of the original draft: IJ, BCB, HW, LF, RM, PM

Review and editing: IJ, BCB, PH, SD, HW, LF, RM, PM

All authors had full access to all the data in the study and had final responsibility for the decision to submit for publication.

## Declarations of interest

IJ and BCB received a research grant from the European Society of Anaesthesiology and Intensive Care (ESAIC) to conduct this study with access to the ESAIC Clinical Trial Network. PM received payments from ESAIC via unrestricted research grant, to pay for research time spent on the project. SD and PH are employed as research staff at ESAIC for running the ESAIC Clinical Trial Network. HW, RM and LF have declared no conflict of interests.
