# Supplementary material for: Vasopressor use after noncardiac surgery: an international observational study
Source: Br J Anaesth. 2025 Jul 11;135(6):1609–17. doi: 10.1016/j.bja.2025.07.034 (PMC12799376; doi:10.1016/j.bja.2025.07.034)
Supplement: Supplementary file 1 — Multimedia component [file mmc1.docx]

**Vasopressor use after non-cardiac surgery: an international observational study.**

**Supplementary Materials**

Table of contents

[**List of collaborators** 2](#_Toc202970160)

[**Endorsements of Specialist and National Anesthesia Associations** 33](#_Toc202970161)

[**Methods** 34](#_Toc202970162)

[**Method S1: In- and exclusion criteria** 34](#_Toc202970163)

[**Method S2: Flow chart for patient recruitment** 35](#_Toc202970164)

[**Method S3: Definition of Postoperative Vasopressor Infusion (PVI)** 36](#_Toc202970165)

[**Method S4: Case Report form** 37](#_Toc202970166)

[**Method S5: Definitions of variables and instructions to investigators** 45](#_Toc202970167)

[**Method S6: Squeeze analysis data set selection** 54](#_Toc202970168)

[**Method S7: Statistical data analysis: methodology** 55](#_Toc202970169)

[**Figures** 58](#_Toc202970170)

[**Figure S1: Observed percentage of PVI by number of patients per hospital.** 58](#_Toc202970171)

[**Figure S2: Observed percentage of PVI use by number of patients in country** 59](#_Toc202970172)

[**Figure S3: Estimated percentage of PVI use in countries with ≥500 patients and ≥6 participating hospitals.** 60](#_Toc202970173)

[**Tables** 61](#_Toc202970174)

[**Table S1: Full Baseline description of the sample including data of missingness** 61](#_Toc202970175)

[**Table S2: Hospital characteristics** 69](#_Toc202970176)

[**Table S3: Overview of Squeeze data** 70](#_Toc202970177)

[**Table S4: Estimated median odds ratios in variation of PVI use** 71](#_Toc202970178)

[**Table S5: Pre-operative predictors of PVI: full model results** 72](#_Toc202970179)

[**Table S6: Pre- and intra-operative predictors of PVI use (full model results)** 74](#_Toc202970180)

[**Table S7: Distributions of outcome measures by Cohort (A or B) and PVI use** 77](#_Toc202970181)

[**Table S8: Full results of outcome models** 78](#_Toc202970182)

[**Table S9: Outcomes by length of vasopressor use (full data for Figure 2)** 89](#_Toc202970183)

[**Table S10** **Type of vasoactive infusion given by day post-surgery (full data for Figure 3)** 91](#_Toc202970184)

[**Table S11: Assessment of postoperative vasopressor infusion need** 92](#_Toc202970185)

[**Table S12: PVI use by MAP target** 93](#_Toc202970186)

# **List of collaborators**

Local investigators and national coordinators (National coordinators in red). Listed in alphabetical order by country and then by center.

| **First name and middle initial(s)** | **Last Name** | **Institution** | **City** | **Country** |
| --- | --- | --- | --- | --- |
| Meriem | Abodun | CHU Saadna Mohamed Abdenour | Sétif | Algeria |
| Souad | Bouaoud | CHU Saadna Mohamed Abdenour | Sétif | Algeria |
| Kamel | Bouchenak | CHU Saadna Mohamed Abdenour | Sétif | Algeria |
| Hind | Saada | CHU Saadna Mohamed Abdenour | Sétif | Algeria |
| Amine | Naili | EPH Sidi Ghiles | Sidi Ghiles | Algeria |
| Shruti | Chitnis | Fiona Stanley Hospital | Murdoch | Australia |
| Marlena | Bartmanska | Fiona Stanley Hospital | Murdoch | Australia |
| Lip-Yong | Choo | Fiona Stanley Hospital | Murdoch | Australia |
| Jolene | Lim | Fiona Stanley Hospital | Murdoch | Australia |
| Estelle | Meirau | Fiona Stanley Hospital | Murdoch | Australia |
| Rhys | Powell | Fiona Stanley Hospital | Murdoch | Australia |
| Erica | Remedios | Fiona Stanley Hospital | Murdoch | Australia |
| Jam | Sadullah | Fiona Stanley Hospital | Murdoch | Australia |
| Alex | Shivarev | Fiona Stanley Hospital | Murdoch | Australia |
| Archana | Shrivathsa | Fiona Stanley Hospital | Murdoch | Australia |
| Marissa | Woodburn | Logan Hospital – Metro South Health Queensland Australia | Meadowbrook | Australia |
| Andrew | Hughes | Logan Hospital – Metro South Health Queensland Australia | Meadowbrook | Australia |
| Benjamin | King-Koi | Logan Hospital – Metro South Health Queensland Australia | Meadowbrook | Australia |
| Anil | Mall | Logan Hospital – Metro South Health Queensland Australia | Meadowbrook | Australia |
| Tharindu | Vithanage | Logan Hospital – Metro South Health Queensland Australia | Meadowbrook | Australia |
| Baraniselvan | Ramalingam | Nepean Hospital, NSW, Australia | Kingswood,NSW | Australia |
| Malcolm Ronald James | Bannerman | Nepean Hospital, NSW, Australia | Kingswood,NSW | Australia |
| Clare Margaret | Shiner | Nepean Hospital, NSW, Australia | Kingswood,NSW | Australia |
| Trylon Matthew | Tsang | Nepean Hospital, NSW, Australia | Kingswood,NSW | Australia |
| David | Highton | Princess Alexandra Hospital, Brisbane Australia | Woolloongabba | Australia |
| Steven | Ayotte | Princess Alexandra Hospital, Brisbane Australia | Woolloongabba | Australia |
| Allison | Kearney | Princess Alexandra Hospital, Brisbane Australia | Woolloongabba | Australia |
| Edward | Thornely | Princess Alexandra Hospital, Brisbane Australia | Woolloongabba | Australia |
| Susanna | Van Haeringen | Princess Alexandra Hospital, Brisbane Australia | Woolloongabba | Australia |
| Amos | Moody | Queen Elizabeth II Jubilee Hospital | Brisbane | Australia |
| Daniel | Kim | Queen Elizabeth II Jubilee Hospital | Brisbane | Australia |
| Claire | Rose | Queen Elizabeth II Jubilee Hospital | Brisbane | Australia |
| Mahmoud | Ugool | Queen Elizabeth II Jubilee Hospital | Brisbane | Australia |
| Will | Zore | Queen Elizabeth II Jubilee Hospital | Brisbane | Australia |
| Andrew | Toner | Royal Perth Hospital, Australia | Perth | Australia |
| Patricia | Anagnostides | Royal Perth Hospital, Australia | Perth | Australia |
| Jodie | Jamieson | Royal Perth Hospital, Australia | Perth | Australia |
| Hilary | Leeson | Royal Perth Hospital, Australia | Perth | Australia |
| Susan | March | Royal Perth Hospital, Australia | Perth | Australia |
| Ronithung | Ovung | Royal Perth Hospital, Australia | Perth | Australia |
| Alessandra | Parini | Royal Perth Hospital, Australia | Perth | Australia |
| Toby | Shipway | Royal Perth Hospital, Australia | Perth | Australia |
| Wai Phen Arthur | Teo | Royal Perth Hospital, Australia | Perth | Australia |
| Huw | Wilkins | Royal Perth Hospital, Australia | Perth | Australia |
| Kahina | Wotton-Hamrioui | Royal Perth Hospital, Australia | Perth | Australia |
| Jodie | Jamieson | Sir Charles Gairdner Hospital | Perth | Australia |
| Sarah | Liew | Sir Charles Gairdner Hospital | Perth | Australia |
| Ashleigh | Cargill | Sir Charles Gairdner Hospital | Perth | Australia |
| Dale | Currigan | Sir Charles Gairdner Hospital | Perth | Australia |
| Edward | Gomm | Sir Charles Gairdner Hospital | Perth | Australia |
| Calvin | Lo | Sir Charles Gairdner Hospital | Perth | Australia |
| Peri | Mickle | Sir Charles Gairdner Hospital | Perth | Australia |
| Marli | Smit | Sir Charles Gairdner Hospital | Perth | Australia |
| Simon | Bradbeer | St Vincent’s Hospital Melbourne | Melbourne | Australia |
| Paul | Köglberger | Klinikum Wels-Grieskirchen Gmbh | Wels | Austria |
| Thomas | Geitmann | Klinikum Wels-Grieskirchen Gmbh | Wels | Austria |
| Laurenz | Hell | Klinikum Wels-Grieskirchen Gmbh | Wels | Austria |
| Johann | Knotzer | Klinikum Wels-Grieskirchen Gmbh | Wels | Austria |
| Dimitar | Tonev | University Hospital "Tsaritsa Yoanna - ISUL" | Sofia | Bulgaria |
| Tanislav | Ilchev | University Hospital "Tsaritsa Yoanna - ISUL" | Sofia | Bulgaria |
| Dimitrinka | Todorova | University Hospital "Tsaritsa Yoanna - ISUL" | Sofia | Bulgaria |
| Karim | Ladha | Saint Michael's Hospital | Toronto | Canada |
| Ciara | Hanley | Saint Michael's Hospital | Toronto | Canada |
| Gabriella | Mattina | Saint Michael's Hospital | Toronto | Canada |
| Janneth | Pazmino-Canizares | Saint Michael's Hospital | Toronto | Canada |
| Bijan | Teja | Saint Michael's Hospital | Toronto | Canada |
| Matteo | Parotto | Toronto General Hospital | Toronto | Canada |
| Samareh | Ajami | Toronto General Hospital | Toronto | Canada |
| Humara | Poonawala | Toronto General Hospital | Toronto | Canada |
| Carlos Jose | Perez Rivera | Fundacion Cardioinfantil | Bogota | Colombia |
| Laura | Ramirez | Fundacion Cardioinfantil | Bogota | Colombia |
| Juan P. | Garcia-Mendez | Fundacion Cardioinfantil | Bogota | Colombia |
| Sharon | Idarraga | Fundacion Cardioinfantil | Bogota | Colombia |
| Ileana | Lulic | Clinical Hospital Merkur | Zagreg | Croatia |
| Gorana | Fingler | Clinical Hospital Merkur | Zagreg | Croatia |
| Jadranka | Pavicic Saric | Clinical Hospital Merkur | Zagreg | Croatia |
| Jakov | Jozić | University Hospital Sveti Duh | Zagreg | Croatia |
| Višnja | Nesek Adam | University Hospital Sveti Duh | Zagreg | Croatia |
| Tatjana | Goranović | University Hospital Sveti Duh | Zagreg | Croatia |
| Marija | Josipović | University Hospital Sveti Duh | Zagreg | Croatia |
| Ida | Kožul | University Hospital Sveti Duh | Zagreg | Croatia |
| Tina | Tomić Mahečić | University Hospital Centre Zagreb | Zagreg | Croatia |
| Leonora | Bračun | University Hospital Centre Zagreb | Zagreg | Croatia |
| Josip | Kovačević | University Hospital Centre Zagreb | Zagreg | Croatia |
| Katarina | Lojna | University Hospital Centre Zagreb | Zagreg | Croatia |
| Anton | Šarčević | University Hospital Centre Zagreb | Zagreg | Croatia |
| Marko | Tripković | University Hospital Centre Zagreb | Zagreg | Croatia |
| Karlo | Uroda | University Hospital Centre Zagreb | Zagreg | Croatia |
| Olav Lilleholt | Schjørring | Aalborg University Hospital | Aalborg | Denmark |
| Steen Kåre | Fagerberg | Aalborg University Hospital | Aalborg | Denmark |
| Birgitte | Brandsborg | Aarhus University Hospital | Aarhus N | Denmark |
| Zidryne | Karaliunaite | Aarhus University Hospital | Aarhus N | Denmark |
| Jens Aage | Kølsen-Petersen | Aarhus University Hospital | Aarhus N | Denmark |
| Christian | Melchior Olesen | Aarhus University Hospital | Aarhus N | Denmark |
| Mikkel Andreas | Strømgaard Andersen | Aarhus University Hospital | Aarhus N | Denmark |
| Henrik | Wolsted | Bispebjerg Og Frederiksberg Hospital | Copenhagen | Denmark |
| Aleksander | Fjeld Haugstvedt | Bispebjerg Og Frederiksberg Hospital | Copenhagen | Denmark |
| Stefan | Gärtner | Bispebjerg Og Frederiksberg Hospital | Copenhagen | Denmark |
| Christine | Hangaard Hansen | Bispebjerg Og Frederiksberg Hospital | Copenhagen | Denmark |
| Mirjana | Cihoric | Copenhagen University Hospital Hvidovre | Copenhagen | Denmark |
| Nicolai Bang | Foss | Copenhagen University Hospital Hvidovre | Copenhagen | Denmark |
| Amalie | Rosendahl | Copenhagen University Hospital Herlev | Copenhagen | Denmark |
| Laurits S. | Kromberg | Copenhagen University Hospital Herlev | Copenhagen | Denmark |
| Marina A. | Nielsen | Copenhagen University Hospital Herlev | Copenhagen | Denmark |
| Bjarne O. | Nielsen | Copenhagen University Hospital Herlev | Copenhagen | Denmark |
| Morten | Vester-Andersen | Copenhagen University Hospital Herlev | Copenhagen | Denmark |
| Rasmus Philip | Nielsen | Gødstrup Hospital | Herning | Denmark |
| Katrine Maul | Andersen | Gødstrup Hospital | Herning | Denmark |
| Mark | Billmann | Gødstrup Hospital | Herning | Denmark |
| Lars H | Lundstrøm | Nordsjællands Hospital | Hillerød | Denmark |
| Gine | Glargaard | Nordsjællands Hospital | Hillerød | Denmark |
| Christine N | Svendsen | Nordsjællands Hospital | Hillerød | Denmark |
| Michael | Bøndergaard | Regional Hospital Randers | Randers | Denmark |
| Jacob | Steinmetz | Rigshospitalet | Copenhagen | Denmark |
| Felicia | Dinesen | Rigshospitalet | Copenhagen | Denmark |
| Liva Thoft | Jensen | Rigshospitalet | Copenhagen | Denmark |
| Lars Simon | Rasmussen | Rigshospitalet | Copenhagen | Denmark |
| L. Andreas H. | Burén | The Regional Hospital In Horsens, Dept. Of Anaesthesia | Horsen | Denmark |
| Yomna E. | Dean | Alexandria Main University Hospital | Alexandria | Egypt |
| Elsakka | Abdelrahman | Alexandria Main University Hospital | Alexandria | Egypt |
| S. Rozan | Samah | Alexandria Main University Hospital | Alexandria | Egypt |
| Rozza | Hebatullah | Alexandria Main University Hospital | Alexandria | Egypt |
| Sabry | Ahmed | Alexandria Main University Hospital | Alexandria | Egypt |
| Shehata | Sameh | Alexandria Main University Hospital | Alexandria | Egypt |
| Shehata | Mostafa | Alexandria Main University Hospital | Alexandria | Egypt |
| Talat | Nesreen | Alexandria Main University Hospital | Alexandria | Egypt |
| Dina | Ramadan | Alexandria Main University Hospital | Alexandria | Egypt |
| Mohamed | Shemies | Alexandria Main University Hospital | Alexandria | Egypt |
| Yousef | Tanas | Alexandria Main University Hospital | Alexandria | Egypt |
| Ahmed | Abbas | Assiut University Hospital | Assiut | Egypt |
| Mostafa | Abbas | Assiut University Hospital | Assiut | Egypt |
| Gena | Elassall | Assiut University Hospital | Assiut | Egypt |
| Saied | Elsawy | Assiut University Hospital | Assiut | Egypt |
| Ramy | Hassan | Assiut University Hospital | Assiut | Egypt |
| Magdy | Mahdy | Assiut University Hospital | Assiut | Egypt |
| Fatma | Monib | Assiut University Hospital | Assiut | Egypt |
| Abdelrahman | Ramdan | Assiut University Hospital | Assiut | Egypt |
| Mahmoud | Saad | Assiut University Hospital | Assiut | Egypt |
| Khaled | Abdelwahab | Mansoura Oncology Center - Mansoura Universty | Mansoura | Egypt |
| Ahmed | Eid | Mansoura Oncology Center - Mansoura Universty | Mansoura | Egypt |
| Omar | Hamdy | Mansoura Oncology Center - Mansoura Universty | Mansoura | Egypt |
| Eman | Mansour | Mansoura Oncology Center - Mansoura Universty | Mansoura | Egypt |
| Moataz Maher | Emara | Mansoura University Gastrointestinal Surgery Center – Mansoura University Faculty Of Medicine | Mansoura | Egypt |
| Mohamed | Bonna | Mansoura University Gastrointestinal Surgery Center – Mansoura University Faculty Of Medicine | Mansoura | Egypt |
| Maiseloon | Mogahed | Mansoura University Gastrointestinal Surgery Center – Mansoura University Faculty Of Medicine | Mansoura | Egypt |
| Hamza | Asmaa | Menoufia University Hospital | Shebin Elkom | Egypt |
| Salma | Elnoamany | Menoufia University Hospital | Shebin Elkom | Egypt |
| Zeinab | Ismail | Menoufia University Hospital | Shebin Elkom | Egypt |
| Mohamed | Sameh | Menoufia University Hospital | Shebin Elkom | Egypt |
| Eman Ibrahim | El-Desoki Mahmoud | National Hepatology & Tropical Medicine Research Institute | Cairo | Egypt |
| Ahmed | Hegazi | National Hepatology & Tropical Medicine Research Institute | Cairo | Egypt |
| Ahmed | Samy | National Hepatology & Tropical Medicine Research Institute | Cairo | Egypt |
| Aiman | Al-Touny | Anesthesia And Intensive Care Department, Faculty Of Medicine, Suez Canal University | Ismaïlia | Egypt |
| Shimaa | Al-Touny | Anesthesia And Intensive Care Department, Faculty Of Medicine, Suez Canal University | Ismaïlia | Egypt |
| Eman | Teema | Anesthesia And Intensive Care Department, Faculty Of Medicine, Suez Canal University | Ismaïlia | Egypt |
| Edmundo | Pereira De Souza Neto | Centre Hospitalier De Montauban | Montauban | France |
| Kevin | Arandel | Centre Hospitalier De Montauban | Montauban | France |
| Remi | Bouquerel | Centre Hospitalier De Montauban | Montauban | France |
| Benjamin | Le Gaillard | Centre Hospitalier De Montauban | Montauban | France |
| Christophe | Pelletier | Centre Hospitalier De Montauban | Montauban | France |
| Antoine | Strzelecki | Centre Hospitalier De Montauban | Montauban | France |
| Emmanuel | Boselli | Centre Hospitalier Pierre Oudot | Bourgoin-Jallieu | France |
| Nicolas | Chardon | Centre Hospitalier Pierre Oudot | Bourgoin-Jallieu | France |
| Pierre | Rodriguez | Centre Hospitalier Pierre Oudot | Bourgoin-Jallieu | France |
| Guillaume | Besch | Centre Hospitalier Universitaire De Besancon | Besancon | France |
| Julien | Villeneuve | Centre Hospitalier Universitaire De Besancon | Besancon | France |
| Grégoire | Wallon | Centre Léon Bérard | Lyon | France |
| Mathilde | Lefevre | Centre Léon Bérard | Lyon | France |
| Pierre-Grégoire | Guinot | CHU Dijon | Dijon | France |
| Belaid | Bouhemad | CHU Dijon | Dijon | France |
| Maxime | Nguyen | CHU Dijon | Dijon | France |
| Guillaume | Raveau | CHU Dijon | Dijon | France |
| Gilles | Lebuffe | CHU Lille | Lille | France |
| Hélène | Beloeil | CHU Rennes | Rennes | France |
| Ludovic | Meuret | CHU Rennes | Rennes | France |
| Bounes | Fanny | CHU Toulouse Rangueil | Toulouse | France |
| Nicolas | Ducrocq | Clinique Du Millénaire - Montpellier - France | Montpellier | France |
| Philippe | Guerci | Institut Lorrain Du Cœur Et Des Vaisseaux - University Hospital Of Nancy | Nancy | France |
| Fanny | Crouton | Institut Lorrain Du Cœur Et Des Vaisseaux - University Hospital Of Nancy | Nancy | France |
| Stephanie | Chevalier | La Sagesse, Rennes | Rennes | France |
| Marc | Anger | La Sagesse, Rennes | Rennes | France |
| Marc | Danguy Des Deserts | Military Hospital Clermont-Tonnerre | Brest | France |
| Philippe | Aries | Military Hospital Clermont-Tonnerre | Brest | France |
| Nicolas | Herzog | Military Hospital Clermont-Tonnerre | Brest | France |
| Johan | Schmitt | Military Hospital Clermont-Tonnerre | Brest | France |
| Xavier | Tete | Military Hospital Clermont-Tonnerre | Brest | France |
| Sascha | Treskatsch | Charité - Universitätsmedizin Berlin | Berlin | Germany |
| Golschan | Asgarpur | Charité - Universitätsmedizin Berlin | Berlin | Germany |
| Tobias | Schäzl | Charité - Universitätsmedizin Berlin | Berlin | Germany |
| Theodor | Kempe | Charité - Universitätsmedizin Berlin | Berlin | Germany |
| Philipp | Brandhorst | Charité - Universitätsmedizin Berlin | Berlin | Germany |
| Henriette | Hegermann | Charité - Universitätsmedizin Berlin | Berlin | Germany |
| Oliver | Hölsken | Charité - Universitätsmedizin Berlin | Berlin | Germany |
| Bernadette | Kleikamp | Charité - Universitätsmedizin Berlin | Berlin | Germany |
| Sophie | Reimers | Charité - Universitätsmedizin Berlin | Berlin | Germany |
| Lars | Bergmann | Universitätsklinikum Knappschaftskrankenhaus Bochum | Bochum | Germany |
| Andreas | Mania | Universitätsklinikum Knappschaftskrankenhaus Bochum | Bochum | Germany |
| Christoph | Sponholz | Jena University Hospital | Jena | Germany |
| Amir | Ali Akbari | Justus-Liebig-University Giessen | Gießen | Germany |
| Moritz | Herzberg | Justus-Liebig-University Giessen | Gießen | Germany |
| Ann-Catrin | Paul | Justus-Liebig-University Giessen | Gießen | Germany |
| Götz | Schmidt | Justus-Liebig-University Giessen | Gießen | Germany |
| Emmanuel | Schneck | Justus-Liebig-University Giessen | Gießen | Germany |
| Christian | Koch | Justus-Liebig-University Giessen | Gießen | Germany |
| Marit | Habicher | Justus-Liebig-University Giessen | Gießen | Germany |
| Michael | Sander | Justus-Liebig-University Giessen | Gießen | Germany |
| Heinrich | Klingler | Klinikum Oldenburg AÖR | Oldenburg | Germany |
| Mareike | Diekmann | Klinikum Oldenburg AÖR | Oldenburg | Germany |
| Sebastian | Schmid | Universitätsklinikum Ulm | Ulm | Germany |
| Raimund | Huf | Universitätsklinikum Ulm | Ulm | Germany |
| Benedikt | Schick | Universitätsklinikum Ulm | Ulm | Germany |
| Julia | Wallqvist | University Hospital RWTH Aachen | Aachen | Germany |
| Kowark | Ana | University Hospital RWTH Aachen | Aachen | Germany |
| Linda | Grüßer | University Hospital RWTH Aachen | Aachen | Germany |
| Rolf | Rossaint | University Hospital RWTH Aachen | Aachen | Germany |
| Hanna | Schröder | University Hospital RWTH Aachen | Aachen | Germany |
| Sebastian | Ziemann | University Hospital RWTH Aachen | Aachen | Germany |
| Daniel | Reuter | University Medicine Rostock | Rostock | Germany |
| Annika | Haas | University Medicine Rostock | Rostock | Germany |
| Bernd | Saugel | University Medical Center Hamburg-Eppendorf | Hamburg | Germany |
| Tom | Daubenfeld | University Medical Center Hamburg-Eppendorf | Hamburg | Germany |
| Moritz | Flick | University Medical Center Hamburg-Eppendorf | Hamburg | Germany |
| Alina | Kröker | University Medical Center Hamburg-Eppendorf | Hamburg | Germany |
| Lorenz | Rosenau | University Medical Center Hamburg-Eppendorf | Hamburg | Germany |
| Christina | Vokuhl | University Medical Center Hamburg-Eppendorf | Hamburg | Germany |
| Mirja | Wegge | University Medical Center Hamburg-Eppendorf | Hamburg | Germany |
| Luisa | Weskamm | University Medical Center Hamburg-Eppendorf | Hamburg | Germany |
| Kassiani | Theodoraki | Arataieion University Hospital, Athens, Greece | Athens | Greece |
| Sofia | Apostolidou | Arataieion University Hospital, Athens, Greece | Athens | Greece |
| George | Gkiokas | Arataieion University Hospital, Athens, Greece | Athens | Greece |
| Konstantinos | Stamatis | Arataieion University Hospital, Athens, Greece | Athens | Greece |
| Chrysoula | Stachtari | General Hospital Of Thessaloniki Georgios Papanikolaou. | Thessaloniki | Greece |
| Meltem | Perente | General Hospital Of Thessaloniki Georgios Papanikolaou. | Thessaloniki | Greece |
| Georgios | Pistiolas | General Hospital Of Thessaloniki Georgios Papanikolaou. | Thessaloniki | Greece |
| Charalampos | Martinos | Naval And Veterans Hopistal Of Athens | Athens | Greece |
| Theodoros | Aslanidis | Agios Pavlos General Hospital Of Thessaloniki | Thessaloniki | Greece |
| Eirini | Sidiropoulou | Agios Pavlos General Hospital Of Thessaloniki | Thessaloniki | Greece |
| Anna | Efthymiou | Saint Savvas Hospital | Athens | Greece |
| Ghrysanthi | Sklavou | Saint Savvas Hospital | Athens | Greece |
| Nikolaos | Barbetakis | Theagenio Cancer Hospital | Thessaloniki | Greece |
| Apostolos | Gogakos | Theagenio Cancer Hospital | Thessaloniki | Greece |
| Achilleas | Lazopoulos | Theagenio Cancer Hospital | Thessaloniki | Greece |
| Eleni | Mavroudi | Theagenio Cancer Hospital | Thessaloniki | Greece |
| Dimitrios | Paliouras | Theagenio Cancer Hospital | Thessaloniki | Greece |
| Thomas | Rallis | Theagenio Cancer Hospital | Thessaloniki | Greece |
| Evangelia | Samara | Tzaneio General Hospital Piraeus | Piraeus | Greece |
| Ioanna | Iatrelli | Tzaneio General Hospital Piraeus | Piraeus | Greece |
| Eleni | Panagiotou | Tzaneio General Hospital Piraeus | Piraeus | Greece |
| Eumorfia | Kondili | University Hospital Of Heraklion Crete | Heraklion | Greece |
| Anthoula | Ntakoula | University Hospital Of Heraklion Crete | Heraklion | Greece |
| Eleftherios | Papadakis | University Hospital Of Heraklion Crete | Heraklion | Greece |
| Konstantinos | Sorokos | University Hospital Of Heraklion Crete | Heraklion | Greece |
| Martin I | Sigurdsson | Landspitali University Hospital | Reykjavik | Iceland |
| Helgi | Egilsson | Landspitali University Hospital | Reykjavik | Iceland |
| Piyush | Ranjan | All India Institute Of Medical Sciences, New Delhi, India | New Delhi | India |
| Puneet | Khanna | All India Institute Of Medical Sciences, New Delhi, India | New Delhi | India |
| Arun | Kumar | All India Institute Of Medical Sciences, New Delhi, India | New Delhi | India |
| Ashu Sara | Mathai | Believers Church Medical College Hospital | Thiruvalla | India |
| Gincy Ann | Lukachan | Believers Church Medical College Hospital | Thiruvalla | India |
| Radhika | Nair | Believers Church Medical College Hospital | Thiruvalla | India |
| Kalpana | Balakrishnan | Cancer Institute (Women's India Association) | Chennai | India |
| Punitha | Chockalingam | Cancer Institute (Women's India Association) | Chennai | India |
| Shah | Bhagyesh | Cims hospital | Ahmedabad | India |
| Edward Johnson | Joseph | Kanyakumari Govt Medical College, Tamil Nadu | Nagercoil | India |
| Veena | Gopal | Nanjappa Multispeciality Hospital | Shimoga,karnataka | India |
| Arjun | Bhagavath K R | Nanjappa Multispeciality Hospital | Shimoga,karnataka | India |
| Shivakumar | Channabasappa | Subbaiah Institute Of Medical Sciences | Shivamogga | India |
| Pooja | Shah | Subbaiah Institute Of Medical Sciences | Shivamogga | India |
| Najah | Hadi | Al-Sadr Medical City Teaching Hospital In Najaf | Najaf | Iraq |
| Ali Najeh | Al-Awwady | Al-Sadr Medical City Teaching Hospital In Najaf | Najaf | Iraq |
| Maytham Aqeel | Al-Juaifari | Al-Sadr Medical City Teaching Hospital In Najaf | Najaf | Iraq |
| Angelo | Giacomucci | Azienda Ospedaliera Di Perugia | Perugia | Italy |
| Francesco | Brunelli | Azienda Ospedaliera Di Perugia | Perugia | Italy |
| Elisa | Scarpone | Azienda Ospedaliera Di Perugia | Perugia | Italy |
| Elena Giovanna | Bignami | Azienda Ospedaliero-Universitaria Di Parma - University Of Parma | Parma | Italy |
| Valentina | Bellini | Azienda Ospedaliero-Universitaria Di Parma - University Of Parma | Parma | Italy |
| Andrea | Bonetti | Azienda Ospedaliero-Universitaria Di Parma - University Of Parma | Parma | Italy |
| Jessica | Colla | Azienda Ospedaliero-Universitaria Di Parma - University Of Parma | Parma | Italy |
| Savino | Spadaro | Azienda Ospedaliera Universitaria Di Ferrara | Ferrara | Italy |
| Giacomo | Baldisserotto | Azienda Ospedaliera Universitaria Di Ferrara | Ferrara | Italy |
| Paolo | Priani | Azienda Ospedaliera Universitaria Di Ferrara | Ferrara | Italy |
| Margherita | Sella | Azienda Ospedaliera Universitaria Di Ferrara | Ferrara | Italy |
| Andrea | Russo | Fondazione Policlinico A. Gemelli IRCSS | Rome | Italy |
| Laura | Cascarano | Fondazione Policlinico A. Gemelli IRCSS | Rome | Italy |
| Bruno | Romanò | Fondazione Policlinico A. Gemelli IRCSS | Rome | Italy |
| Giulia | Torregiani | IFO Regina Elena | Rome | Italy |
| Maurizio | Cecconi | IRCCS Humanitas Research Hospital | Milan | Italy |
| Massimiliano | Greco | IRCCS Humanitas Research Hospital | Milan | Italy |
| Nicolò | Martinetti | IRCCS Humanitas Research Hospital | Milan | Italy |
| Sergio | Palma | IRCCS Humanitas Research Hospital | Milan | Italy |
| Andrea | Pradella | IRCCS Humanitas Research Hospital | Milan | Italy |
| Rosella | Nicoletti | Ospedale Madonna Delle Grazie | Matera | Italy |
| Barbara | Bacer | Ospedale Maggiore Carlo Alberto Pizzardi | Bologna | Italy |
| Martina | Guarnera | Ospedale Maggiore Carlo Alberto Pizzardi | Bologna | Italy |
| Michela | Lotierzo | Ospedale Maggiore Carlo Alberto Pizzardi | Bologna | Italy |
| Sara | Miori | Ospedale Santa Chiara | Trento | Italy |
| Sergio | Lassola | Ospedale Santa Chiara | Trento | Italy |
| Andrea | Sanna | Ospedale Santa Chiara | Trento | Italy |
| Iacopo | Cappellini | Ospedale Santo Stefano | Prato | Italy |
| Filippo | Becherucci | Ospedale Santo Stefano | Prato | Italy |
| Lucia | Zamidei | Ospedale Santo Stefano | Prato | Italy |
| Guglielmo | Consales | Ospedale Santo Stefano | Prato | Italy |
| Lorenzo | Tutino | Ospedale Santo Stefano | Prato | Italy |
| Luigi | Vetrugno | Ospedale Universitario “Santa Maria Della Misericordia” Di Udine | Udine | Italy |
| Gloria | Marson | Ospedale Universitario “Santa Maria Della Misericordia” Di Udine | Udine | Italy |
| Gianluca | Zani | Santa Maria Delle Croci Hospital, Ravenna | Ravenna | Italy |
| Giulia | Felloni | Santa Maria Delle Croci Hospital, Ravenna | Ravenna | Italy |
| Maurizio | Fusari | Santa Maria Delle Croci Hospital, Ravenna | Ravenna | Italy |
| Claudio | Gecele | Santa Maria Delle Croci Hospital, Ravenna | Ravenna | Italy |
| Massimo | Terenzoni | Santa Maria Delle Croci Hospital, Ravenna | Ravenna | Italy |
| Andrea | Cortegiani | University Hospital Policlinico P. Giaccone | Palermo | Italy |
| Giulia | Catalisano | University Hospital Policlinico P. Giaccone | Palermo | Italy |
| Tatiana | Catania Cucchiara | University Hospital Policlinico P. Giaccone | Palermo | Italy |
| Dario Calogero | Fricano | University Hospital Policlinico P. Giaccone | Palermo | Italy |
| Giulia | Ingoglia | University Hospital Policlinico P. Giaccone | Palermo | Italy |
| Mariachiara | Ippolito | University Hospital Policlinico P. Giaccone | Palermo | Italy |
| Claudia | Marino | University Hospital Policlinico P. Giaccone | Palermo | Italy |
| Gabriele | Presti | University Hospital Policlinico P. Giaccone | Palermo | Italy |
| Lucia | Mirabella | University Of Foggia | Foggia | Italy |
| Antonio | De Candia | University Of Foggia | Foggia | Italy |
| Nicole | Pepe | University Of Foggia | Foggia | Italy |
| Kiyoyasu | Kurahashi | International University of Health and Welfare (IUHW), School of Medicine | Narita | Japan |
| Munehito | Uchiyama | International University Of Health And Welfare Narita Hospital | Narita | Japan |
| Hiroshi | Morimatsu | Okayama University Hospital | Okayama | Japan |
| Kosuke | Kuroda | Okayama University Hospital | Okayama | Japan |
| Kaori | Yamashita | Okayama University Hospital | Okayama | Japan |
| Tatsuya | Kida | Yokosuka Kyosai Hospital | Yokosuka | Japan |
| Tomohide | Takei | Yokosuka Kyosai Hospital | Yokosuka | Japan |
| Sohaib | Al-Omary | Princess Basma Teaching Hospital | Irbid | Jordan |
| Lara | Alnajjar Lara | Princess Basma Teaching Hospital | Irbid | Jordan |
| Majjd | Alnajjar Lara | Princess Basma Teaching Hospital | Irbid | Jordan |
| Amro | Abuleil | Royal medical services, Amman | Amman | Jordan |
| Antigona | Hasani | American Hospital Kosovo & Faculty Of Medicine, University Of Prishtina | Pristina | Kosovo |
| Marin | Almahroush | Abu-Salim Trauma Hospital | Tripoli | Libya |
| Marya | Bensalem | Abu-Salim Trauma Hospital | Tripoli | Libya |
| Mawadaa | Alttir | Abu-Salim Trauma Hospital | Tripoli | Libya |
| Muhammed | Elhadi | Faculty Medicine University of Tripoli | Tripoli | Libya |
| Akram | Alkseek | Gharyan Central Hospital | Gharyan | Libya |
| Hibah Bileid | Bakeer | Gharyan Central Hospital | Gharyan | Libya |
| Eman | Abdulwahed | Tripoli Central Hospital | Tripoli | Libya |
| Entisar | Alshareea | Tripoli Central Hospital | Tripoli | Libya |
| Reem | Ghmagh | Tripoli Central Hospital | Tripoli | Libya |
| Doaa | Gidiem | Tripoli Central Hospital | Tripoli | Libya |
| Enas | Soula | Tripoli Central Hospital | Tripoli | Libya |
| Mohd Zulfakar | Mazlan | Universiti Sains Malaysia | Kota Bharu | Malaysia |
| Sanihah | Che Omar | Universiti Sains Malaysia | Kota Bharu | Malaysia |
| Mohamad Hasyizan | Hassan | Universiti Sains Malaysia | Kota Bharu | Malaysia |
| Shamsul Kamalrujan | Hassan | Universiti Sains Malaysia | Kota Bharu | Malaysia |
| Huda | Zainal Abidin | Universiti Sains Malaysia | Kota Bharu | Malaysia |
| Ion | Chesov | Chisinau City Hospital No.1 | Chisinau | Moldova |
| Mihai | Tiple | Chisinau City Hospital No.1 | Chisinau | Moldova |
| Natalia | Zadiraca | Chisinau City Hospital No.1 | Chisinau | Moldova |
| Diana | Boleac | Institute Of Emergency Medicine | Chisinau | Moldova |
| Doina | Oglinda | Institute Of Emergency Medicine | Chisinau | Moldova |
| Abdelghafour | El Koundi | Military Teaching Hospital Mohammed V | Rabat | Morocco |
| Hicham | Balkhi | Military Teaching Hospital Mohammed V | Rabat | Morocco |
| Mustapha | Bensghir | Military Teaching Hospital Mohammed V | Rabat | Morocco |
| Noureddine | Kartite | Military Teaching Hospital Mohammed V | Rabat | Morocco |
| Abdelilah | Ghannam | National Institute Of Oncology Of Rabat" - "Mohammed V University In Rabat - National Institute Of Oncology Of Rabat | Rabat | Morocco |
| Othman | Belarabi | National Institute Of Oncology Of Rabat" - "Mohammed V University In Rabat - National Institute Of Oncology Of Rabat | Rabat | Morocco |
| Zakaria | Belkhadir | National Institute Of Oncology Of Rabat" - "Mohammed V University In Rabat - National Institute Of Oncology Of Rabat | Rabat | Morocco |
| Brahim | El Ahmadi | National Institute Of Oncology Of Rabat" - "Mohammed V University In Rabat - National Institute Of Oncology Of Rabat | Rabat | Morocco |
| Elisavet | Karkala | Akershus Universitetssykehus | Nordbyhagen | Norway |
| Maria Christina | Ravn | Akershus Universitetssykehus | Nordbyhagen | Norway |
| Oda Uhlin | Husebekk | Alesund Sjukehus | Alesund | Norway |
| Renate | Johnsen | Alesund Sjukehus | Alesund | Norway |
| Ib | Jammer | Haukeland University Hospital, Bergen | Bergen | Norway |
| Vegard | Lundevall | Haukeland University Hospital, Bergen | Bergen | Norway |
| Wiszt | Radovan | Innlandet Hospital Trust | Elverum | Norway |
| Andreas | Haugerud | Innlandet Hospital Trust | Elverum | Norway |
| Ine Karoline | Stenersen | Innlandet Hospital Trust | Elverum | Norway |
| Agnete | Prydz | Østfold Hospital Kalnes | Grålum | Norway |
| David Frederic | Knutsen | Østfold Hospital Kalnes | Grålum | Norway |
| Heidi Marthea | Ohnstad | Østfold Hospital Kalnes | Grålum | Norway |
| Roy B. | Olsen | Sorlandet Hospital Arendal, Norway | Arendal | Norway |
| Elise Runde | Krogstad | Sorlandet Hospital Arendal, Norway | Arendal | Norway |
| Anna | Sigurdardottir | Sorlandet Hospital Arendal, Norway | Arendal | Norway |
| Krzych | Łukasz | Central Clinical Centre, Medical University Of Silesia | Katowice | Poland |
| Michal | Szewczyk | Central Clinical Centre, Medical University Of Silesia | Katowice | Poland |
| Cristina | Granja | Centro Hospitalar do Algarve, Faro | Faro | Portugal |
| Catarina | Dourado | Centro Hospitalar E Universitário De Coimbra (CHUC) | Coimbra | Portugal |
| Nidia | Gonçalves | Centro Hospitalar E Universitário De Coimbra (CHUC) | Coimbra | Portugal |
| Francisco | Matias | Centro Hospitalar E Universitário De Coimbra (CHUC) | Coimbra | Portugal |
| Ana | Raimundo | Centro Hospitalar E Universitário De Coimbra (CHUC) | Coimbra | Portugal |
| Rui Pedro | Cunha | Centro Hospitalar Lisboa Ocidental, Lisbon | Lisbon | Portugal |
| Miguel | Tavares | Centro Hospitalar Universitário Do Porto - Hospital Santo António | Porto | Portugal |
| Alexandre | Pinto | Centro Hospitalar Universitário Do Porto - Hospital Santo António | Porto | Portugal |
| Cristina | Torrão | Centro Hospitalar Universitário Do Porto - Hospital Santo António | Porto | Portugal |
| Lnês | Amaral | Centro Hospitalar Universitário Do Porto - Hospital Santo António | Porto | Portugal |
| Ana Rita | Costa | Centro Hospitalar Universitário Do Porto - Hospital Santo António | Porto | Portugal |
| Ricardo | Marinho | Centro Hospitalar Universitário Do Porto - Hospital Santo António | Porto | Portugal |
| Miguel | Ricardo | Centro Hospitalar Universitário Do Porto - Hospital Santo António | Porto | Portugal |
| César | Vidal | Centro Hospitalar Universitário Do Porto - Hospital Santo António | Porto | Portugal |
| Alice | Santos | Centro Hospitalar Universitario Sao Joao | Porto | Portugal |
| Julia | Mendonça | Centro Hospitalar Universitario Sao Joao | Porto | Portugal |
| Daniela | Xara | Centro Hospitalar Universitario Sao Joao | Porto | Portugal |
| Raul | Neto | Centro Hospitalar Vila Nova Gaia/ Espinho | Vila Nova de Gaia | Portugal |
| João Tiago | Rodrigues | Centro Hospitalar Vila Nova Gaia/ Espinho | Vila Nova de Gaia | Portugal |
| Ricardo | Amaral | Unidade Local De Saúde De Trás-Os-Montes E Alto Douro, Portugal. | Vila Real | Portugal |
| Diogo | Oliveira | Unidade Local De Saúde De Trás-Os-Montes E Alto Douro, Portugal. | Vila Real | Portugal |
| José | Sampaio | Unidade Local De Saúde De Trás-Os-Montes E Alto Douro, Portugal. | Vila Real | Portugal |
| Francisca | Cardoso | Hospital Santa Luzia, ULSAM – Viana Do Castelo | Viana do castelo | Portugal |
| José | Caldeiro | Hospital Santa Luzia, ULSAM – Viana Do Castelo | Viana do castelo | Portugal |
| Rogério | Corga | Hospital Santa Luzia, ULSAM – Viana Do Castelo | Viana do castelo | Portugal |
| Edite | Mendes | Hospital Santa Luzia, ULSAM – Viana Do Castelo | Viana do castelo | Portugal |
| Pedro | Moura | Hospital Santa Luzia, ULSAM – Viana Do Castelo | Viana do castelo | Portugal |
| Rita | Passos | Hospital Santa Luzia, ULSAM – Viana Do Castelo | Viana do castelo | Portugal |
| Francisco | Silva | Hospital Santa Luzia, ULSAM – Viana Do Castelo | Viana do castelo | Portugal |
| Sofia | Trovisco | Instituto Português De Oncologia Do Porto | Porto | Portugal |
| Inês | Fonseca | Instituto Português De Oncologia Do Porto | Porto | Portugal |
| Décio | Pereira | Instituto Português De Oncologia Do Porto | Porto | Portugal |
| Lina | Miranda | Instituto Português De Oncologia Do Porto | Porto | Portugal |
| Muhammad Shakeel | Riaz | Hamad Medical Corporation | Doha | Qatar |
| Hamed | Elgendy | Hamad Medical Corporation | Doha | Qatar |
| Hashaam | Ghafoor | Hamad Medical Corporation | Doha | Qatar |
| Mohammed | Haji | Hamad Medical Corporation | Doha | Qatar |
| Vipin | Kumari | Hamad Medical Corporation | Doha | Qatar |
| Lakshmi | Ramanathan | Hamad Medical Corporation | Doha | Qatar |
| Jassim | Rauf | Hamad Medical Corporation | Doha | Qatar |
| Nissar | Shaikh | Hamad Medical Corporation | Doha | Qatar |
| Abdul Gafoor | Tharayil | Hamad Medical Corporation | Doha | Qatar |
| Marija | Toleska | University Clinical Center "Mother Teresa" Skopje; University "Ss. Cyril And Methodius" Skopje, Macedonia | Skopje | Republic Of North Macedonia |
| Aleksandar | Dimitrovski | University Clinical Center "Mother Teresa" Skopje; University "Ss. Cyril And Methodius" Skopje, Macedonia | Skopje | Republic Of North Macedonia |
| Filip | Naumovski | University Clinical Center "Mother Teresa" Skopje; University "Ss. Cyril And Methodius" Skopje, Macedonia | Skopje | Republic Of North Macedonia |
| Angela | Trposka | University Clinical Center "Mother Teresa" Skopje; University "Ss. Cyril And Methodius" Skopje, Macedonia | Skopje | Republic Of North Macedonia |
| Ioana Marina | Grintescu | Clinical Emergency Hospital Of Bucharest | Bucharest | Romania |
| Cristian | Cobilinschi | Clinical Emergency Hospital Of Bucharest | Bucharest | Romania |
| Ana-Maria | Cotae | Clinical Emergency Hospital Of Bucharest | Bucharest | Romania |
| Liliana | Mirea | Clinical Emergency Hospital Of Bucharest | Bucharest | Romania |
| Raluca | Ungureanu | Clinical Emergency Hospital Of Bucharest | Bucharest | Romania |
| Liana | Valeanu | Emergency Institute For Cardiovascular Diseases CC Iliescu | Bucharest | Romania |
| Bianca | Morosanu | Emergency Institute For Cardiovascular Diseases CC Iliescu | Bucharest | Romania |
| Serban | Bubenek-Turtoni | Emergency Institute For Cardiovascular Diseases CC Iliescu | Bucharest | Romania |
| Cornel | Robu | Emergency Institute For Cardiovascular Diseases CC Iliescu | Bucharest | Romania |
| Alida | Moise | Prof. Dr. Gerota Hospital | Bucharest | Romania |
| Carmen | Balescu | Prof. Dr. Gerota Hospital | Bucharest | Romania |
| Catalin Traian | Guran | Prof. Dr. Gerota Hospital | Bucharest | Romania |
| Madalina | Herman | Prof. Dr. Gerota Hospital | Bucharest | Romania |
| Alexander | Kulikov | Burdenko National Medical Research Centre Of Neurosurgery, Moscow | Moscow | Russia |
| Igor | Zabolotskikh | Kuban State Medical University With Clinical Facility "Territorial Hospital #2" | Krasnodar | Russia |
| Dmitriy | Fedunets | Kuban State Medical University With Clinical Facility "Territorial Hospital #2" | Krasnodar | Russia |
| Nikita | Trembach | Kuban State Medical University With Clinical Facility "Territorial Hospital #2" | Krasnodar | Russia |
| Valerii | Subbotin | Moscow Clinical Scientific Center Na Loginov | Moscow | Russia |
| Ilyas | Izmailov | Moscow Clinical Scientific Center Na Loginov | Moscow | Russia |
| Maria | Miroshnichenko | Moscow Clinical Scientific Center Na Loginov | Moscow | Russia |
| Ekaterina | Orlova | Moscow Clinical Scientific Center Na Loginov | Moscow | Russia |
| Elizaveta | Serdobintseva | Moscow Clinical Scientific Center Na Loginov | Moscow | Russia |
| Mikhail | Kirov | Northern State Medical University | Arkhangelsk | Russia |
| Aleksey | Avidzba | Northern State Medical University | Arkhangelsk | Russia |
| Vsevolod | Kuzkov | Northern State Medical University | Arkhangelsk | Russia |
| Anton | Nikonov | Northern State Medical University | Arkhangelsk | Russia |
| Sergey | Astrakov | Novosibirsk State University With Clinical Facility City Clinical Hospital #25 | Novosibirsk | Russia |
| Elena | Neporada | Novosibirsk State University With Clinical Facility City Clinical Hospital #25 | Novosibirsk | Russia |
| Victoria | Khoronenko | P.A. Herzen Moscow Cancer Research Institute | Moscow | Russia |
| Vladislav | Karpeikin | P.A. Herzen Moscow Cancer Research Institute | Moscow | Russia |
| Anna | Malanova | P.A. Herzen Moscow Cancer Research Institute | Moscow | Russia |
| Pavel | Suvorin | P.A. Herzen Moscow Cancer Research Institute | Moscow | Russia |
| July | Zaharenkova | P.A. Herzen Moscow Cancer Research Institute | Moscow | Russia |
| Sergey | Efremov | Saint Petersburg State University Hospital | Saint-Petersburg | Russia |
| Oleg | Kuleshov | Saint Petersburg State University Hospital | Saint-Petersburg | Russia |
| Alexey | Kulikov | Saint Petersburg State University Hospital | Saint-Petersburg | Russia |
| Elizaveta | Leonova | Saint Petersburg State University Hospital | Saint-Petersburg | Russia |
| Olivera | Marinkovic | CHC Bezaniska Kosa | Belgrade | Serbia |
| Ana | Sekulic | CHC Bezaniska Kosa | Belgrade | Serbia |
| Ivan | Palibrk | Clinic For Digestive Surgery-The First Surgical Clinic, University Clinical Center Serbia | Belgrade | Serbia |
| Marija | Djukanovic | Clinic For Digestive Surgery-The First Surgical Clinic, University Clinical Center Serbia | Belgrade | Serbia |
| Svetlana | Sreckovic | Clinic For Orthopedics Surgery And Traumatology, University Clinical Center Of Serbia | Belgrade | Serbia |
| Radmila | Klacar | Clinic For Orthopedics Surgery And Traumatology, University Clinical Center Of Serbia | Belgrade | Serbia |
| Dragana | Vracevic | Clinic For Orthopedics Surgery And Traumatology, University Clinical Center Of Serbia | Belgrade | Serbia |
| Miodrag | Milenovic | Emergency Center, University Clinical Center Of Serbia; Faculty Of Medicine, University Of Belgrade | Belgrade | Serbia |
| Aleksandra | Nikolic | Emergency Center, University Clinical Center Of Serbia; Faculty Of Medicine, University Of Belgrade | Belgrade | Serbia |
| Marija | Rajkovic | Emergency Center, University Clinical Center Of Serbia; Faculty Of Medicine, University Of Belgrade | Belgrade | Serbia |
| Dragana | Lončar Stojiljković | Institute For Cardiovascular Diseases Belgrade | Belgrade | Serbia |
| Nikola | Djukanović | Institute For Cardiovascular Diseases Belgrade | Belgrade | Serbia |
| Biljana | Novaković | Institute For Cardiovascular Diseases Belgrade | Belgrade | Serbia |
| Maja | Stojanovic | University Clinical Center "Zvezdara" | Belgrade | Serbia |
| Milan | Markovic | University Clinical Center "Zvezdara" | Belgrade | Serbia |
| Slobodan | Popovic | University Clinical Center "Zvezdara" | Belgrade | Serbia |
| Janez | Dolinar | General Hospital Novo Mesto | Novo Mesto | Slovenia |
| Sandra | Blagojević Štembergar | General Hospital Novo Mesto | Novo Mesto | Slovenia |
| Goran | Kurnik | General Hospital Novo Mesto | Novo Mesto | Slovenia |
| Peter | Poredos | University Medical Centre Ljubljana | Ljubljana | Slovenia |
| Vanja | Oven | University Medical Centre Ljubljana | Ljubljana | Slovenia |
| Andreja | Möller Petrun | University Medical Centre Maribor | Maribor | Slovenia |
| Bojana | Drobnjak | University Medical Centre Maribor | Maribor | Slovenia |
| Maša | Furman | University Medical Centre Maribor | Maribor | Slovenia |
| Marko | Lokar | University Medical Centre Maribor | Maribor | Slovenia |
| Jernej | Novak | University Medical Centre Maribor | Maribor | Slovenia |
| Katarina Katja | Primožič | University Medical Centre Maribor | Maribor | Slovenia |
| Palesa | Motshabi Chakane | Charlotte Maxeke Johannesburg Academic Hospital (CMJAH) | Parktown, Johannesburg | South Africa |
| Sithandiwe | Dingezweni | Charlotte Maxeke Johannesburg Academic Hospital (CMJAH) | Parktown, Johannesburg | South Africa |
| Leballo | Gontse | Charlotte Maxeke Johannesburg Academic Hospital (CMJAH) | Parktown, Johannesburg | South Africa |
| Zainub | Jooma | Charlotte Maxeke Johannesburg Academic Hospital (CMJAH) | Parktown, Johannesburg | South Africa |
| Hlamatsi | Moutlana | Charlotte Maxeke Johannesburg Academic Hospital (CMJAH) | Parktown, Johannesburg | South Africa |
| LUNGANGA TOMS | LUSHIKU | Chris Hani Baragwanath Academic Hospital | Soweto, Johannesburg | South Africa |
| GRACE | MANJOORAN | Chris Hani Baragwanath Academic Hospital | Soweto, Johannesburg | South Africa |
| PALESA | MOGANE | Chris Hani Baragwanath Academic Hospital | Soweto, Johannesburg | South Africa |
| MATHABE | SEHLAPELO | Chris Hani Baragwanath Academic Hospital | Soweto, Johannesburg | South Africa |
| Sean | Chetty | Stellenbosch University, Cape Town | Cape Town | South Africa |
| Stephen | Venter | Tygerberg Hospital | Cape Town | South Africa |
| Triesie | Lotz | Tygerberg Hospital | Cape Town | South Africa |
| Pablo | Monedero | Clínica Universidad De Navarra | Pamplona | Spain |
| Carmen | Cara-Gilabert | Clínica Universidad De Navarra | Pamplona | Spain |
| Angela | Escribano-Arranz | Clínica Universidad De Navarra | Pamplona | Spain |
| Marta | Luque-Peláez | Clínica Universidad De Navarra | Pamplona | Spain |
| Pablo | Montero-López | Clínica Universidad De Navarra | Pamplona | Spain |
| Inigo | Iñigo Rubio-Baines | Clínica Universidad De Navarra | Pamplona | Spain |
| Carmen | Sala-Trull | Clínica Universidad De Navarra | Pamplona | Spain |
| Ana María | García Sánchez | Complejo Asistencial De Zamora | Zamora | Spain |
| Cristina | Blanco Dorado | Complejo Asistencial De Zamora | Zamora | Spain |
| Angela | Casquero Murciego | Complejo Asistencial De Zamora | Zamora | Spain |
| Francisco | García Lázaro | Complejo Asistencial De Zamora | Zamora | Spain |
| Yaiza | Molero Diez | Complejo Asistencial De Zamora | Zamora | Spain |
| F Javier | García-Miguel | Complejo Hospitalario Segovia | Segovia | Spain |
| Estefania | Chamorro Garci | Complejo Hospitalario Segovia | Segovia | Spain |
| Rosalia | Navarro-Perez | Hospital Clinico San Carlos | Madrid | Spain |
| Luis | Santé | Hospital Clinico San Carlos | Madrid | Spain |
| Andrea | Gutiérrez | Hospital Clínico Universitario Valencia | Valencia | Spain |
| Marta | Luzón | Hospital Clínico Universitario Valencia | Valencia | Spain |
| Rosalba | Martinez | Hospital Clínico Universitario Valencia | Valencia | Spain |
| Eduardo | Passariello | Hospital Clínico Universitario Valencia | Valencia | Spain |
| Ana | Ruiz | Hospital Clínico Universitario Valencia | Valencia | Spain |
| Ferran | Serralta | Hospital Clínico Universitario Valencia | Valencia | Spain |
| Jaume | Valero | Hospital Clínico Universitario Valencia | Valencia | Spain |
| Susana | Altaba Tena | Hospital General Universitario De Castellón | Castellón de la Plana | Spain |
| Maria Lidon | Mateu Campos | Hospital General Universitario De Castellón | Castellón de la Plana | Spain |
| Luisa | Cueva Castro | Hospital Sant Pau | Barcelona | Spain |
| Albert | Bainac Albadalejo | Hospital Sant Pau | Barcelona | Spain |
| Astrid | Batalla Gonzalez | Hospital Sant Pau | Barcelona | Spain |
| Cecilia | Diez García | Hospital Sant Pau | Barcelona | Spain |
| Marta | Giné Servén | Hospital Sant Pau | Barcelona | Spain |
| Laura | Pardo Pinzón | Hospital Sant Pau | Barcelona | Spain |
| Hector | Villanueva Sanchez | Hospital Sant Pau | Barcelona | Spain |
| Ángel | Becerra-Bolaños | Hospital Universitario De Gran Canaria Doctor Negrin | Las Palmas de Gran Canaria | Spain |
| Antonio | Arencibia-Almeida | Hospital Universitario De Gran Canaria Doctor Negrin | Las Palmas de Gran Canaria | Spain |
| Gema | Hernanz-Rodríguez | Hospital Universitario De Gran Canaria Doctor Negrin | Las Palmas de Gran Canaria | Spain |
| Virginia | Muiño-Palomar | Hospital Universitario De Gran Canaria Doctor Negrin | Las Palmas de Gran Canaria | Spain |
| Nazario | Ojeda-Betancor | Hospital Universitario De Gran Canaria Doctor Negrin | Las Palmas de Gran Canaria | Spain |
| Aurelio | Rodríguez-Pérez | Hospital Universitario De Gran Canaria Doctor Negrin | Las Palmas de Gran Canaria | Spain |
| José Ignacio | García-Sánchez | Hospital Universitario Fundación Alcorcón | Alcorcon | Spain |
| Tamara | Brunete | Hospital Universitario Fundación Alcorcón | Alcorcon | Spain |
| David | Delgado | Hospital Universitario Fundación Alcorcón | Alcorcon | Spain |
| Pablo | Redondo | Hospital Universitario Fundación Alcorcón | Alcorcon | Spain |
| Viviana | Varón | Hospital Universitario Fundación Alcorcón | Alcorcon | Spain |
| Diana | Zamudio | Hospital Universitario Fundación Alcorcón | Alcorcon | Spain |
| Javi | Ripolles | Infanta Leonor Univesrity Hospital - Madrid | Madrid | Spain |
| Susana | González-Suárez | Vall D´Hebron University Hospital | Barcelona | Spain |
| Elena Regla | Gómez-González | Vall D´Hebron University Hospital | Barcelona | Spain |
| María Del Carmen | Iribarren Mateos | Vall D´Hebron University Hospital | Barcelona | Spain |
| Hytham K. S. | Hamid | East Nile Hospital | Khartoum | Sudan |
| Alaa | Musa | East Nile Hospital | Khartoum | Sudan |
| Elfayadh | Saidahmed | East Nile Hospital | Khartoum | Sudan |
| Ahmed | Mohamed Ibrahim Mohamed | Gadarif Teaching Hospital | Gadarif | Sudan |
| Muntasir | Abdelsakhi | Ibn-Sina Specialized Teaching Hospital | Khartoum | Sudan |
| Walaa | Abdelrouf Ibrahim | Ibn-Sina Specialized Teaching Hospital | Khartoum | Sudan |
| Abdulrhman | Khaity | Ibn-Sina Specialized Teaching Hospital | Khartoum | Sudan |
| Michelle | Chew | Department Of Anaesthesiology And Intensive Care Medicine, University Hospital, Linköping | Linköping | Sweden |
| Helen | Didriksson | Department Of Anaesthesiology And Intensive Care Medicine, University Hospital, Linköping | Linköping | Sweden |
| Carina | Jonsson | Department Of Anaesthesiology And Intensive Care Medicine, University Hospital, Linköping | Linköping | Sweden |
| Thorir S. | Sigmundsson | Karolinska University Hospital - Solna | Stockholm | Sweden |
| Anna | Granström | Karolinska University Hospital - Solna | Stockholm | Sweden |
| Malin | Jonsson Fagerlund | Karolinska Institutet and Karolinska University Hospital - Solna | Stockholm | Sweden |
| Anna | Schening | Karolinska University Hospital - Solna | Stockholm | Sweden |
| Arman | Valadkhani | Karolinska University Hospital - Solna | Stockholm | Sweden |
| Christina | Blixt | Karolinska University Hospital Huddinge | Stockholm | Sweden |
| Malin | Hansson | Karolinska University Hospital Huddinge | Stockholm | Sweden |
| Kristina | Kilsand | Karolinska University Hospital Huddinge | Stockholm | Sweden |
| Åke | Norberg | Karolinska University Hospital Huddinge | Stockholm | Sweden |
| Eva | Strandberg | Karolinska University Hospital Huddinge | Stockholm | Sweden |
| Egidijus | Semenas | Uppsala University Hospital | Uppsala | Sweden |
| Lina | Jonikaite | Uppsala University Hospital | Uppsala | Sweden |
| Alexander | Dullenkopf | Spital Thurgau Frauenfeld | Frauenfeld | Switzerland |
| Ivan | Chau | Spital Thurgau Frauenfeld | Frauenfeld | Switzerland |
| Lina | Petersen | Spital Thurgau Frauenfeld | Frauenfeld | Switzerland |
| Benedikt | Preckel | Amsterdam UMC, Location AMC | Amsterdam | The Netherlands |
| Ali | Kaplan | Amsterdam UMC, Location AMC | Amsterdam | The Netherlands |
| Jimmy | Schenk | Amsterdam UMC, Location AMC | Amsterdam | The Netherlands |
| Denise Petra | Veelo | Amsterdam UMC, Location AMC | Amsterdam | The Netherlands |
| Felix | Van Lier | Erasmus MC | Rotterdam | The Netherlands |
| Rene | Van Bruchem | Erasmus MC | Rotterdam | The Netherlands |
| Seppe SHA | Koopman | Maasstad Hospital | Rotterdam | The Netherlands |
| Toine | Van Den Ende | Maasstad Hospital | Rotterdam | The Netherlands |
| Hans D. | De Boer | Martini Hospital Groningen | Groningen | The Netherlands |
| Henriëtte | Smid-Nanninga | Martini Hospital Groningen | Groningen | The Netherlands |
| Paul A. | Van Beest | Medical Center Leeuwarden | Leeuwarden | The Netherlands |
| Eric E.C. | De Waal | University Medical Center Utrecht | Utrecht | The Netherlands |
| Thomas W.L. | Scheeren | University Medical Centre Groningen | Groningen | The Netherlands |
| Ilonka N. | De Keijzer | University Medical Centre Groningen | Groningen | The Netherlands |
| Constanze | Brucker | Wilhelmina Hospital Assen | Assen | The Netherlands |
| Sandra | Brookman | Wilhelmina Hospital Assen | Assen | The Netherlands |
| Inge J.E. | Paas | Wilhelmina Hospital Assen | Assen | The Netherlands |
| Lerzan | Dogan | Acibadem Altunizade Hospital | Istanbul | Turkey |
| Hazal | Yazgec | Acibadem Altunizade Hospital | Istanbul | Turkey |
| Cigdem | Yildirim Guclu | Ankara University Faculty Of Medicine | Ankara | Turkey |
| Sanem | Cakar Turhan | Ankara University Faculty Of Medicine | Ankara | Turkey |
| Basak Ceyda | Meco | Ankara University Faculty Of Medicine | Ankara | Turkey |
| Ali | Alagoz | University Of Health Sciences, Ankara Atatürk Sanatorium Training And Research Hospital | Ankara | Turkey |
| Hilal | Sazak | University Of Health Sciences, Ankara Atatürk Sanatorium Training And Research Hospital | Ankara | Turkey |
| Arzu | Yıldırım Ar | Fatih Sultan Mehmet Health Application Research Center, University Of Health Sciences | Istanbul | Turkey |
| Öznur | Demiroluk | Fatih Sultan Mehmet Health Application Research Center, University Of Health Sciences | Istanbul | Turkey |
| Yıldız | Yiğit | Fatih Sultan Mehmet Health Application Research Center, University Of Health Sciences | Istanbul | Turkey |
| Osman | Ekinci | Haydarpasa Numune Training And Research Hospital | Istanbul | Turkey |
| Serap | Adana Kavlak | Haydarpasa Numune Training And Research Hospital | Istanbul | Turkey |
| Seymanur | Altintas Filizoglu | Haydarpasa Numune Training And Research Hospital | Istanbul | Turkey |
| Günseli | Orhun | Istanbul Faculty Of Medicine, Istanbul University | Istanbul | Turkey |
| Mert | Canbaz | Istanbul Faculty Of Medicine, Istanbul University | Istanbul | Turkey |
| Kemal Tolga | Saracoglu | Kartal Dr Lutfi Kirdar Training And Research Hospital | Istanbul | Turkey |
| Elif | Akova Deniz | Kartal Dr Lutfi Kirdar Training And Research Hospital | Istanbul | Turkey |
| Banu | Eler Cevik | Kartal Dr Lutfi Kirdar Training And Research Hospital | Istanbul | Turkey |
| Ayca Sultan | Sahin | University Of Health Sciences, Kanuni Sultan Suleyman Education And Training Hospital' | Istanbul | Turkey |
| Ebru | Kaya | University Of Health Sciences, Kanuni Sultan Suleyman Education And Training Hospital'' | Istanbul | Turkey |
| Hande | Gurbuz | University Of Health Sciences, Bursa Yuksek Ihtisas Training And Research Hospital | Bursa | Turkey |
| Derya | Karasu | University Of Health Sciences, Bursa Yuksek Ihtisas Training And Research Hospital | Bursa | Turkey |
| Seyda Efsun | Ozgunay | University Of Health Sciences, Bursa Yuksek Ihtisas Training And Research Hospital | Bursa | Turkey |
| Eren Fatma | Akcil | University Of Istanbul - Cerrahpasa | Istanbul | Turkey |
| Ozlem | Korkmaz Dilmen | University Of Istanbul - Cerrahpasa | Istanbul | Turkey |
| Yusuf | Tunali | University Of Istanbul - Cerrahpasa | Istanbul | Turkey |
| Kerem | Erkalp | Bagcilar Training And Educational Hospital | Istanbul | Turkiye |
| Ali | Ozalp | Bagcilar Training And Educational Hospital | Istanbul | Turkiye |
| Mehmet Salih | Sevdi | Bagcilar Training And Educational Hospital | Istanbul | Turkiye |
| Maryna | Freigofer | Dnipro Regional Cancer Center | Dnipro | Ukraine |
| Olena | Khomenko | O.O. Shalimov National Scientific Center Of Surgery And Transplantation | Kyiv | Ukraine |
| Pavlo | Hurin | O.O. Shalimov National Scientific Center Of Surgery And Transplantation | Kyiv | Ukraine |
| Dmytro | Dmytriiev | Vinnitsa National Medical University And Vinnitsa Regional Endocrinology Center | Vinnitsa | Ukraine |
| Eugenii | Lysak | Vinnitsa National Medical University And Vinnitsa Regional Endocrinology Center | Vinnitsa | Ukraine |
| Tamsin | Gregory | Airedale NHS Foundation Trust | Keighley | United Kingdom |
| Shaw | Alison | Airedale NHS Foundation Trust | Keighley | United Kingdom |
| Ratcliffe | Anita | Airedale NHS Foundation Trust | Keighley | United Kingdom |
| Hairsine | Brigid | Airedale NHS Foundation Trust | Keighley | United Kingdom |
| Adam | Farrar | Airedale NHS Foundation Trust | Keighley | United Kingdom |
| Samson A. | Williams | Airedale NHS Foundation Trust | Keighley | United Kingdom |
| Joyce | Yeung | Birmingham Heartlands Hospital | Birmingham | United Kingdom |
| Syed | Abid | Birmingham Heartlands Hospital | Birmingham | United Kingdom |
| Adetoro | Akintunde | Birmingham Heartlands Hospital | Birmingham | United Kingdom |
| Roshni | Bahri | Birmingham Heartlands Hospital | Birmingham | United Kingdom |
| Marta | Burak | Birmingham Heartlands Hospital | Birmingham | United Kingdom |
| Libby | Dias | Birmingham Heartlands Hospital | Birmingham | United Kingdom |
| Yash | Dinesh | Birmingham Heartlands Hospital | Birmingham | United Kingdom |
| Iman | Farah | Birmingham Heartlands Hospital | Birmingham | United Kingdom |
| Ciara | Gibson | Birmingham Heartlands Hospital | Birmingham | United Kingdom |
| Joanne | Gresty | Birmingham Heartlands Hospital | Birmingham | United Kingdom |
| Fiona | Harris | Birmingham Heartlands Hospital | Birmingham | United Kingdom |
| Alex | Jones | Birmingham Heartlands Hospital | Birmingham | United Kingdom |
| Chuck | Lam | Birmingham Heartlands Hospital | Birmingham | United Kingdom |
| William | Mciver | Birmingham Heartlands Hospital | Birmingham | United Kingdom |
| Teresa | Melody | Birmingham Heartlands Hospital | Birmingham | United Kingdom |
| Ninoshka | Merchant | Birmingham Heartlands Hospital | Birmingham | United Kingdom |
| Safwaan | Patel | Birmingham Heartlands Hospital | Birmingham | United Kingdom |
| Gursharan | Virdee | Birmingham Heartlands Hospital | Birmingham | United Kingdom |
| Bryan | Wong | Birmingham Heartlands Hospital | Birmingham | United Kingdom |
| Jessica | Davis | Bolton Hospital NHS Foundation Trust | Farnworth | United Kingdom |
| Jordan | Alfonso | Bolton Hospital NHS Foundation Trust | Farnworth | United Kingdom |
| Mohamed | Elbahnasy | Bolton Hospital NHS Foundation Trust | Farnworth | United Kingdom |
| Monica | Trivedi | Cambridge University Hospitals Trust | Cambridge | United Kingdom |
| Efthymia Maria | Kapasouri | Cambridge University Hospitals Trust | Cambridge | United Kingdom |
| Galina | Maneva | Cambridge University Hospitals Trust | Cambridge | United Kingdom |
| Peta | Masters | Cambridge University Hospitals Trust | Cambridge | United Kingdom |
| Malgorzata | Opalinska | Cambridge University Hospitals Trust | Cambridge | United Kingdom |
| Luke | Winslow | Countess Of Chester NHS Foundation Trust | Chester | United Kingdom |
| Bamford | Peter | Countess Of Chester NHS Foundation Trust | Chester | United Kingdom |
| Prince | Judith | Countess Of Chester NHS Foundation Trust | Chester | United Kingdom |
| Faulkner | Maria | Countess Of Chester NHS Foundation Trust | Chester | United Kingdom |
| Ivison | Alison | Countess Of Chester NHS Foundation Trust | Chester | United Kingdom |
| Barham | Elin | Countess Of Chester NHS Foundation Trust | Chester | United Kingdom |
| Barton | Matthew | Countess Of Chester NHS Foundation Trust | Chester | United Kingdom |
| Hadlett | Max | Countess Of Chester NHS Foundation Trust | Chester | United Kingdom |
| Russell | Nicki | Countess Of Chester NHS Foundation Trust | Chester | United Kingdom |
| Verghese | Prashant | Countess Of Chester NHS Foundation Trust | Chester | United Kingdom |
| Karunaratne | Nicholas | Countess Of Chester NHS Foundation Trust | Chester | United Kingdom |
| Murphy | Thomas | Countess Of Chester NHS Foundation Trust | Chester | United Kingdom |
| Sundar | Ashok | Croydon University Hospital | Thornton Heath | United Kingdom |
| Christopher | Black | Croydon University Hospital | Thornton Heath | United Kingdom |
| Zakaulla | Belagodu | Dartford And Gravesham NHS Trust | Dartford | United Kingdom |
| Ryan | Coe | Dartford And Gravesham NHS Trust | Dartford | United Kingdom |
| Katy | Collins | Dartford And Gravesham NHS Trust | Dartford | United Kingdom |
| Tracy | Edmunds | Dartford And Gravesham NHS Trust | Dartford | United Kingdom |
| Charlotte | Kamundi | Dartford And Gravesham NHS Trust | Dartford | United Kingdom |
| Prasanna | Patlola | Dartford And Gravesham NHS Trust | Dartford | United Kingdom |
| Laura | Johnson | Dartford And Gravesham NHS Trust | Dartford | United Kingdom |
| Naomi | Oakley | Dartford And Gravesham NHS Trust | Dartford | United Kingdom |
| Olumide | Olufuwa | Dartford And Gravesham NHS Trust | Dartford | United Kingdom |
| Luciana | Rusu | Dartford And Gravesham NHS Trust | Dartford | United Kingdom |
| Juleen | Fasham | Derriford Hospital | Plymouth | United Kingdom |
| Amit | Das | Derriford Hospital | Plymouth | United Kingdom |
| Anna | Ratcliffe | Derriford Hospital | Plymouth | United Kingdom |
| Ben | Parish | Derriford Hospital | Plymouth | United Kingdom |
| Freeman | Lizzie | Derriford Hospital | Plymouth | United Kingdom |
| Gary | Minto | Derriford Hospital | Plymouth | United Kingdom |
| Gunarathna | Perumbadage | Derriford Hospital | Plymouth | United Kingdom |
| Jessica | Sinclair | Derriford Hospital | Plymouth | United Kingdom |
| Lucy | Guile | Derriford Hospital | Plymouth | United Kingdom |
| Matthew | Baldwin | Derriford Hospital | Plymouth | United Kingdom |
| Stephanie | Pauling | Derriford Hospital | Plymouth | United Kingdom |
| Wael | Alhalabi | Derriford Hospital | Plymouth | United Kingdom |
| Moustafa | Shebl Zahra | East Kent Hospitals University NHS Foundation Trust (EKHUFT) Margate Kent | Canterbury | United Kingdom |
| Eva | Beranova | East Kent Hospitals University NHS Foundation Trust (EKHUFT) Margate Kent | Canterbury | United Kingdom |
| Tracy | Hazelton | East Kent Hospitals University NHS Foundation Trust (EKHUFT) Margate Kent | Canterbury | United Kingdom |
| Alicia | Knight | East Kent Hospitals University NHS Foundation Trust (EKHUFT) Margate Kent | Canterbury | United Kingdom |
| Trudy | Parfrey | East Kent Hospitals University NHS Foundation Trust (EKHUFT) Margate Kent | Canterbury | United Kingdom |
| Jhanielle | Quindoyos | East Kent Hospitals University NHS Foundation Trust (EKHUFT) Margate Kent | Canterbury | United Kingdom |
| Hazel | Ramos | East Kent Hospitals University NHS Foundation Trust (EKHUFT) Margate Kent | Canterbury | United Kingdom |
| Gabriella | Tutt | East Kent Hospitals University NHS Foundation Trust (EKHUFT) Margate Kent | Canterbury | United Kingdom |
| Joanne | Deery | East Kent Hospitals University NHS Foundation Trust (EKHUFT) Margate Kent | Canterbury | United Kingdom |
| Himanshu | Arora | East Kent Hospitals University NHS Foundation Trust (EKHUFT) Margate Kent | Canterbury | United Kingdom |
| David | Freeman | East Lancashire Hospitals NHS Trust | Blackburn | United Kingdom |
| Qasim Tayyib | Ahmed | East Lancashire Hospitals NHS Trust | Blackburn | United Kingdom |
| Alexander | Gurnee | East Lancashire Hospitals NHS Trust | Blackburn | United Kingdom |
| Rachel | Harding | East Lancashire Hospitals NHS Trust | Blackburn | United Kingdom |
| Tom | Mckernan | East Lancashire Hospitals NHS Trust | Blackburn | United Kingdom |
| Aayesha | Kazi | East Lancashire Hospitals NHS Trust | Blackburn | United Kingdom |
| Nicholas | Truman | East Lancashire Hospitals NHS Trust | Blackburn | United Kingdom |
| Stephen | Lewis | Frimley Park Hospital | Frimley | United Kingdom |
| Eid | Ahmed | Frimley Park Hospital | Frimley | United Kingdom |
| Baiju | Barath | Frimley Park Hospital | Frimley | United Kingdom |
| Bernardo | Solomon | Frimley Park Hospital | Frimley | United Kingdom |
| Hau Lam Clara | Fong | Frimley Park Hospital | Frimley | United Kingdom |
| Stevenson | Joe | Frimley Park Hospital | Frimley | United Kingdom |
| Katarzyna Anna | Marasinska | Frimley Park Hospital | Frimley | United Kingdom |
| Abelarde | Kaye | Frimley Park Hospital | Frimley | United Kingdom |
| Essuman | Lorinda | Frimley Park Hospital | Frimley | United Kingdom |
| Whitmarsh | Thomas | Frimley Park Hospital | Frimley | United Kingdom |
| Jack | Tooze | Frimley Park Hospital | Frimley | United Kingdom |
| Bland | Yvonne | Frimley Park Hospital | Frimley | United Kingdom |
| Andrew | Lowes | Gateshead Health NHS Foundation Trust (Queen Elizabeth Hospital) | Queen | United Kingdom |
| Mohamed | Abdelsalam | Gateshead Health NHS Foundation Trust (Queen Elizabeth Hospital) | Queen | United Kingdom |
| Jon | Braviner | Gateshead Health NHS Foundation Trust (Queen Elizabeth Hospital) | Queen | United Kingdom |
| Rachael | Lucas | Gateshead Health NHS Foundation Trust (Queen Elizabeth Hospital) | Queen | United Kingdom |
| Jenny | Ritzema | Gateshead Health NHS Foundation Trust (Queen Elizabeth Hospital) | Queen | United Kingdom |
| Nicolas | Simmers | Gateshead Health NHS Foundation Trust (Queen Elizabeth Hospital) | Queen | United Kingdom |
| Manushi | Vyas | Gateshead Health NHS Foundation Trust (Queen Elizabeth Hospital) | Queen | United Kingdom |
| Venkat | Sundaram | Glan Clwyd Hospital | Bodelwyddan | United Kingdom |
| Anette | Bolger | Glan Clwyd Hospital | Bodelwyddan | United Kingdom |
| Jennifer | Davies | Glan Clwyd Hospital | Bodelwyddan | United Kingdom |
| Esther | Garrod | Glan Clwyd Hospital | Bodelwyddan | United Kingdom |
| Victoria | Garvey | Glan Clwyd Hospital | Bodelwyddan | United Kingdom |
| Rachel | Manley | Glan Clwyd Hospital | Bodelwyddan | United Kingdom |
| Zuzana | Probier | Glan Clwyd Hospital | Bodelwyddan | United Kingdom |
| Angela | Pye | Glan Clwyd Hospital | Bodelwyddan | United Kingdom |
| Zoka | Milan | King's College Hospital | London | United Kingdom |
| Gudrun | Kunst | King's College Hospital | London | United Kingdom |
| Daveena | Meeks | King's College Hospital | London | United Kingdom |
| Anna | Broderick | King's College Hospital | London | United Kingdom |
| Kevin | O'Reilly | King's College Hospital | London | United Kingdom |
| Juliana | Pereira | King's College Hospital | London | United Kingdom |
| Bernd Oliver | Rose | Lewisham & Greenwich NHS Trust - Queen Elizabeth Hospital | London | United Kingdom |
| Leanne | Howard | Lewisham & Greenwich NHS Trust - Queen Elizabeth Hospital | London | United Kingdom |
| Estefania | Treus | Lewisham & Greenwich NHS Trust - Queen Elizabeth Hospital | London | United Kingdom |
| Teodora | Orasanu | Lincoln County Hospital | Lincoln | United Kingdom |
| Russell | Conyers | Lincoln County Hospital | Lincoln | United Kingdom |
| Katie | Dorr | Lincoln County Hospital | Lincoln | United Kingdom |
| Ellie | Farcas | Lincoln County Hospital | Lincoln | United Kingdom |
| Olesya | Francis | Lincoln County Hospital | Lincoln | United Kingdom |
| Kelly | Hubbard | Lincoln County Hospital | Lincoln | United Kingdom |
| Rachel | Newton | Lincoln County Hospital | Lincoln | United Kingdom |
| Sarah | Shephardson | Lincoln County Hospital | Lincoln | United Kingdom |
| Catherine | Wyatt | Lincoln County Hospital | Lincoln | United Kingdom |
| David | Golden | Maidstone Hospital | Maidstone | United Kingdom |
| Amy | Ackerley | Maidstone Hospital | Maidstone | United Kingdom |
| Laura | Adams | Maidstone Hospital | Maidstone | United Kingdom |
| Jennifer | Assimakopoulos | Maidstone Hospital | Maidstone | United Kingdom |
| Miriam | Davey | Maidstone Hospital | Maidstone | United Kingdom |
| Maddie | Lawrence | Maidstone Hospital | Maidstone | United Kingdom |
| Rebecca | Seaman | Maidstone Hospital | Maidstone | United Kingdom |
| Michala | Shah | Maidstone Hospital | Maidstone | United Kingdom |
| Heather | Callaghan | Maidstone Hospital | Maidstone | United Kingdom |
| Kailash | Bhatia | Manchester Royal Infirmary | Manchester | United Kingdom |
| Mohamed | Abdelmotieleb | Manchester Royal Infirmary | Manchester | United Kingdom |
| Victor | Bill | Manchester Royal Infirmary | Manchester | United Kingdom |
| Ayman | Edarous | Manchester Royal Infirmary | Manchester | United Kingdom |
| Samuel | Ikenga | Manchester Royal Infirmary | Manchester | United Kingdom |
| Rose | Jama | Manchester Royal Infirmary | Manchester | United Kingdom |
| Kezia | Philipose | Manchester Royal Infirmary | Manchester | United Kingdom |
| Manu | Sudevan | Manchester Royal Infirmary | Manchester | United Kingdom |
| Brendan | Sloan | Mid Yorkshire NHS Trust | Wakefield | United Kingdom |
| Sarah | Buckley | Mid Yorkshire NHS Trust | Wakefield | United Kingdom |
| Anna | Littlejohns | Mid Yorkshire NHS Trust | Wakefield | United Kingdom |
| Amy | Major | Mid Yorkshire NHS Trust | Wakefield | United Kingdom |
| Lauren | Tye | Mid Yorkshire NHS Trust | Wakefield | United Kingdom |
| Manoj | Wickramasinghe | Mid Yorkshire NHS Trust | Wakefield | United Kingdom |
| Katie | Wilson | Mid Yorkshire NHS Trust | Wakefield | United Kingdom |
| Richard | Stewart | Milton Keynes University Hospital | Milton Keynes | United Kingdom |
| Teena | Babu | Milton Keynes University Hospital | Milton Keynes | United Kingdom |
| Louise | Mew | Milton Keynes University Hospital | Milton Keynes | United Kingdom |
| Alistair | Sawyerr | North Manchester General Hospital | Manchester | United Kingdom |
| Sharon | Baxter-Dore | North Manchester General Hospital | Manchester | United Kingdom |
| Nowfal | Rahman | North Manchester General Hospital | Manchester | United Kingdom |
| Joanne | Rothwell | North Manchester General Hospital | Manchester | United Kingdom |
| Helen | T-Michael | North Manchester General Hospital | Manchester | United Kingdom |
| Shiny | Sivanandan | North West Anglia NHS Foundation Trust | Peterborough | United Kingdom |
| Kirsty | Allen | North West Anglia NHS Foundation Trust | Peterborough | United Kingdom |
| Daniele | Arcoria | North West Anglia NHS Foundation Trust | Peterborough | United Kingdom |
| Roberta | De Pretto | North West Anglia NHS Foundation Trust | Peterborough | United Kingdom |
| Gbemisola | Jenfa | North West Anglia NHS Foundation Trust | Peterborough | United Kingdom |
| Zoey | Horne | North West Anglia NHS Foundation Trust | Peterborough | United Kingdom |
| Zainab | Mavani | North West Anglia NHS Foundation Trust | Peterborough | United Kingdom |
| Graeme | Mclintock | North West Anglia NHS Foundation Trust | Peterborough | United Kingdom |
| Emmah | Nelly | North West Anglia NHS Foundation Trust | Peterborough | United Kingdom |
| Ionela | Sinanovic | North West Anglia NHS Foundation Trust | Peterborough | United Kingdom |
| Natalie | Temple | North West Anglia NHS Foundation Trust | Peterborough | United Kingdom |
| Josephine | Williams | North West Anglia NHS Foundation Trust | Peterborough | United Kingdom |
| Anand | Jayaraman | Northumbria Healthcare NHS Foundation Trust | Newcastle | United Kingdom |
| Joshua | Craig | Northumbria Healthcare NHS Foundation Trust | Newcastle | United Kingdom |
| Hayley | Mckie | Northumbria Healthcare NHS Foundation Trust | Newcastle | United Kingdom |
| Tracy | Smith | Northumbria Healthcare NHS Foundation Trust | Newcastle | United Kingdom |
| Gail | Waddell | Northumbria Healthcare NHS Foundation Trust | Newcastle | United Kingdom |
| Trish | Tsuro | Pilgrim Hospital | Boston | United Kingdom |
| Khaled | Ahmed | Pilgrim Hospital | Boston | United Kingdom |
| Alya | Amin | Pilgrim Hospital | Boston | United Kingdom |
| Kimberley | Netherton | Pilgrim Hospital | Boston | United Kingdom |
| Izuchukwu | Nwalusi | Pilgrim Hospital | Boston | United Kingdom |
| Bryony | Saint | Pilgrim Hospital | Boston | United Kingdom |
| Kinga | Szymiczek | Pilgrim Hospital | Boston | United Kingdom |
| Reschreiter | Henrik | Poole Hospital (University Hospitals Dorset) | Poole | United Kingdom |
| Leanne | Bartlett | Poole Hospital (University Hospitals Dorset) | Poole | United Kingdom |
| Yasmin | De'Ath | Poole Hospital (University Hospitals Dorset) | Poole | United Kingdom |
| Charlotte | Humphrey | Poole Hospital (University Hospitals Dorset) | Poole | United Kingdom |
| Emma | Langridge | Poole Hospital (University Hospitals Dorset) | Poole | United Kingdom |
| Rebecca | Miln | Poole Hospital (University Hospitals Dorset) | Poole | United Kingdom |
| Tomasz | Torlinski | QEHB University Hospitals Birmingham NHS FT | Birmingham | United Kingdom |
| Tony | Whitehouse | QEHB University Hospitals Birmingham NHS FT | Birmingham | United Kingdom |
| Ian | Ewington | Queen Elizabeth Hospital Birmingham | Birmingham | United Kingdom |
| Phillip | Howells | Queen Elizabeth Hospital Birmingham | Birmingham | United Kingdom |
| Randeep | Mullhi | Queen Elizabeth Hospital Birmingham | Birmingham | United Kingdom |
| Amit | Sharma | Queen Elizabeth Hospital Birmingham | Birmingham | United Kingdom |
| Hazel | Smith | Queen Elizabeth Hospital Birmingham | Birmingham | United Kingdom |
| Carla | Speziale | Queen Elizabeth Hospital Birmingham | Birmingham | United Kingdom |
| Julian | Giles | Queen Victoria Hospital NHS Foundation Trust | E Grinstead | United Kingdom |
| Joel | Lockwood | Queen Victoria Hospital NHS Foundation Trust | E Grinstead | United Kingdom |
| Henrik | Reschreiter | Royal Bournemouth Hospital (University Hospitals Dorset) | Bournemouth | United Kingdom |
| Chloe | Bascombe | Royal Bournemouth Hospital (University Hospitals Dorset) | Bournemouth | United Kingdom |
| Claire | Osey | Royal Bournemouth Hospital (University Hospitals Dorset) | Bournemouth | United Kingdom |
| Debbie | Branney | Royal Bournemouth Hospital (University Hospitals Dorset) | Bournemouth | United Kingdom |
| Tiller | Heather | Royal Bournemouth Hospital (University Hospitals Dorset) | Bournemouth | United Kingdom |
| Javen | Ramsami | Royal Bournemouth Hospital (University Hospitals Dorset) | Bournemouth | United Kingdom |
| Sally | Pitts | Royal Bournemouth Hospital (University Hospitals Dorset) | Bournemouth | United Kingdom |
| Annamaria | Wilce | Royal Bournemouth Hospital (University Hospitals Dorset) | Bournemouth | United Kingdom |
| Natalie | Agius | Royal Bournemouth Hospital (University Hospitals Dorset) | Bournemouth | United Kingdom |
| Lindsay | Rogers | Royal Bournemouth Hospital (University Hospitals Dorset) | Bournemouth | United Kingdom |
| Cheryl | Lindsay | Royal Bournemouth Hospital (University Hospitals Dorset) | Bournemouth | United Kingdom |
| Claire | Preedy | Royal Cornwall Hospital NHS Trust | Truro | United Kingdom |
| Luke | Hayward | Royal Cornwall Hospital NHS Trust | Truro | United Kingdom |
| Thomas | Clark | Royal Devon & Exeter Hospital | Exeter | United Kingdom |
| Kevin | Windsor | Royal Devon & Exeter Hospital | Exeter | United Kingdom |
| Kizzy | Baines | Royal Devon & Exeter Hospital | Exeter | United Kingdom |
| Ben | Dingle | Royal Devon & Exeter Hospital | Exeter | United Kingdom |
| Rebecca | Wilcock | Royal Devon & Exeter Hospital | Exeter | United Kingdom |
| Hemal | Bosamia | Royal Devon & Exeter Hospital | Exeter | United Kingdom |
| Toby | Lewis | Royal Devon & Exeter Hospital | Exeter | United Kingdom |
| Ingeborg | Welters | University of Liverpool | Liverpool | United Kingdom |
| Richard | Ramsaran | Royal Liverpool And Broadgreen University Hospital Trusts | Liverpool | United Kingdom |
| Aleem | Morenikeji | Royal Liverpool And Broadgreen University Hospital Trusts | Liverpool | United Kingdom |
| Annie | [Smith](mailto:Anniesmith@doctors.org.uk) | Royal Liverpool And Broadgreen University Hospital Trusts | Liverpool | United Kingdom |
| Maria Arra Carlota | Canada | Royal Liverpool And Broadgreen University Hospital Trusts | Liverpool | United Kingdom |
| Claire | Davies | Royal Liverpool And Broadgreen University Hospital Trusts | Liverpool | United Kingdom |
| Dan | Watkin | Royal Liverpool And Broadgreen University Hospital Trusts | Liverpool | United Kingdom |
| Jon | Machin | Royal Liverpool And Broadgreen University Hospital Trusts | Liverpool | United Kingdom |
| Katherine | [Hodson](mailto:Katherine.Hodson@liverpoolft.nhs.uk) | Royal Liverpool And Broadgreen University Hospital Trusts | Liverpool | United Kingdom |
| Maria | Lopez | Royal Liverpool And Broadgreen University Hospital Trusts | Liverpool | United Kingdom |
| Luke | Shearer | Royal Liverpool And Broadgreen University Hospital Trusts | Liverpool | United Kingdom |
| Nick | Sinanan | Royal Liverpool And Broadgreen University Hospital Trusts | Liverpool | United Kingdom |
| Maria | Norris | Royal Liverpool And Broadgreen University Hospital Trusts | Liverpool | United Kingdom |
| Rebecca | Vickers | Royal Liverpool And Broadgreen University Hospital Trusts | Liverpool | United Kingdom |
| David | Shaw | Royal Liverpool And Broadgreen University Hospital Trusts | Liverpool | United Kingdom |
| Victoria | Waugh | Royal Liverpool And Broadgreen University Hospital Trusts | Liverpool | United Kingdom |
| Karen | Williams | Royal Liverpool And Broadgreen University Hospital Trusts | Liverpool | United Kingdom |
| Hayaka | Amada | Royal Liverpool And Broadgreen University Hospital Trusts | Liverpool | United Kingdom |
| Alexander | Sell | Royal National Orthopedic Hospital | Stanmore | United Kingdom |
| Tuyen | Anthony | Royal National Orthopedic Hospital | Stanmore | United Kingdom |
| Anamaria | Gerea | Royal National Orthopedic Hospital | Stanmore | United Kingdom |
| Shamil | Tanna | Royal National Orthopedic Hospital | Stanmore | United Kingdom |
| Angus | Tulloch | Royal National Orthopedic Hospital | Stanmore | United Kingdom |
| Panagiota | Alexopoulou | Royal Surrey County Hospital NHS Foundation Trust | Guildford | United Kingdom |
| Naomi | Boyer | Royal Surrey County Hospital NHS Foundation Trust | Guildford | United Kingdom |
| Paula | Carvelli | Royal Surrey County Hospital NHS Foundation Trust | Guildford | United Kingdom |
| Benedikt | Creagh-Brown | Royal Surrey County Hospital NHS Foundation Trust | Guildford | United Kingdom |
| Olivia | Dow | Royal Surrey County Hospital NHS Foundation Trust | Guildford | United Kingdom |
| Fouad | El-Hibri | Royal Surrey County Hospital NHS Foundation Trust | Guildford | United Kingdom |
| Syeda | Haider | Royal Surrey County Hospital NHS Foundation Trust | Guildford | United Kingdom |
| James | Hilton | Royal Surrey County Hospital NHS Foundation Trust | Guildford | United Kingdom |
| Hannah | Mackay | Royal Surrey County Hospital NHS Foundation Trust | Guildford | United Kingdom |
| Wisdom | Mbama | Royal Surrey County Hospital NHS Foundation Trust | Guildford | United Kingdom |
| Natalia | Michalak | Royal Surrey County Hospital NHS Foundation Trust | Guildford | United Kingdom |
| Maskell | Nick | Royal Surrey County Hospital NHS Foundation Trust | Guildford | United Kingdom |
| Kanji | Rafiq | Royal Surrey County Hospital NHS Foundation Trust | Guildford | United Kingdom |
| Donna-May | Sanga | Royal Surrey County Hospital NHS Foundation Trust | Guildford | United Kingdom |
| Nasser | Syed | Royal Surrey County Hospital NHS Foundation Trust | Guildford | United Kingdom |
| Jerik | Verula | Royal Surrey County Hospital NHS Foundation Trust | Guildford | United Kingdom |
| Waldtraud | Wutte | Royal Surrey County Hospital NHS Foundation Trust | Guildford | United Kingdom |
| Pierson | Richard | Russells Hall Hospital | Dudley | United Kingdom |
| Butler | Jack | Russells Hall Hospital | Dudley | United Kingdom |
| Elena | Anastasescu | Russells Hall Hospital | Dudley | United Kingdom |
| Tania | Mellor | Russells Hall Hospital | Dudley | United Kingdom |
| Wallbridge | Thomas | Russells Hall Hospital | Dudley | United Kingdom |
| Emma | Ingall | The William Harvey Hospital | Ashford | United Kingdom |
| Kim | Jemmett | The William Harvey Hospital | Ashford | United Kingdom |
| Vicki | Priestly | The William Harvey Hospital | Ashford | United Kingdom |
| James | Rand | The William Harvey Hospital | Ashford | United Kingdom |
| Maxime | Rigaudy | The William Harvey Hospital | Ashford | United Kingdom |
| Reanne | Solly | The William Harvey Hospital | Ashford | United Kingdom |
| Sarah | Stirrup | The William Harvey Hospital | Ashford | United Kingdom |
| Heather | Weston | The William Harvey Hospital | Ashford | United Kingdom |
| Wayne | Evans | The Robert Jones And Agnes Hunt NHS Foundation Trust | Oswestry, Shropshire | United Kingdom |
| Chloe | Perry | The Robert Jones And Agnes Hunt NHS Foundation Trust | Oswestry, Shropshire | United Kingdom |
| Karen | Pilson | The Robert Jones And Agnes Hunt NHS Foundation Trust | Oswestry, Shropshire | United Kingdom |
| Peringathara | Biju | The Royal Orthopaedic Hospital Birmingham | Birmingham | United Kingdom |
| Jones | Claudette | The Royal Orthopaedic Hospital Birmingham | Birmingham | United Kingdom |
| Keeling | Ellie | The Royal Orthopaedic Hospital Birmingham | Birmingham | United Kingdom |
| Jones | James | The Royal Orthopaedic Hospital Birmingham | Birmingham | United Kingdom |
| Brodie | Teresa | The Royal Orthopaedic Hospital Birmingham | Birmingham | United Kingdom |
| Magaya | Valarie | The Royal Orthopaedic Hospital Birmingham | Birmingham | United Kingdom |
| Andrew | Woodgate | Torbay And South Devon NHS Foundation Trust | Torquay | United Kingdom |
| Kylie | Ashby | Torbay And South Devon NHS Foundation Trust | Torquay | United Kingdom |
| Pauline | Aspa | Torbay And South Devon NHS Foundation Trust | Torquay | United Kingdom |
| Kelly | Barrett | Torbay And South Devon NHS Foundation Trust | Torquay | United Kingdom |
| Lauren | Blunt | Torbay And South Devon NHS Foundation Trust | Torquay | United Kingdom |
| Sean | Caunter | Torbay And South Devon NHS Foundation Trust | Torquay | United Kingdom |
| Emily | Flavell | Torbay And South Devon NHS Foundation Trust | Torquay | United Kingdom |
| Peter | Fletcher | Torbay And South Devon NHS Foundation Trust | Torquay | United Kingdom |
| Angie | Foulds | Torbay And South Devon NHS Foundation Trust | Torquay | United Kingdom |
| Ashleigh | Fynn | Torbay And South Devon NHS Foundation Trust | Torquay | United Kingdom |
| Beth | Mcelroy | Torbay And South Devon NHS Foundation Trust | Torquay | United Kingdom |
| Bryony | Reed | Torbay And South Devon NHS Foundation Trust | Torquay | United Kingdom |
| Fleur | Rogers | Torbay And South Devon NHS Foundation Trust | Torquay | United Kingdom |
| Andrea | Ford | Torbay And South Devon NHS Foundation Trust | Torquay | United Kingdom |
| Emma | Bartlett | Torbay And South Devon NHS Foundation Trust | Torquay | United Kingdom |
| David | Golden | Tunbridge Wells Hospital | Tunbridge Wells | United Kingdom |
| Amy | Ackerley | Tunbridge Wells Hospital | Tunbridge Wells | United Kingdom |
| Laura | Adams | Tunbridge Wells Hospital | Tunbridge Wells | United Kingdom |
| Jennifer | Assimakopoulos | Tunbridge Wells Hospital | Tunbridge Wells | United Kingdom |
| Miriam | Davey | Tunbridge Wells Hospital | Tunbridge Wells | United Kingdom |
| Madeleine | Lawrence | Tunbridge Wells Hospital | Tunbridge Wells | United Kingdom |
| Rebecca | Seaman | Tunbridge Wells Hospital | Tunbridge Wells | United Kingdom |
| Michala | Shah | Tunbridge Wells Hospital | Tunbridge Wells | United Kingdom |
| Heather | Callaghan | Tunbridge Wells Hospital | Tunbridge Wells | United Kingdom |
| Bernd Oliver | Rose | University Hospital Lewisham | London | United Kingdom |
| Jacob | Burr | University Hospital Lewisham | London | United Kingdom |
| Rosie | Reece-Anthony | University Hospital Lewisham | London | United Kingdom |
| Georgia | Richmond | University Hospital Lewisham | London | United Kingdom |
| Kay | Spikes | University Hospital Lewisham | London | United Kingdom |
| Eleanor | Stranger | University Hospital Lewisham | London | United Kingdom |
| Danaja | Zolger | University Hospital Lewisham | London | United Kingdom |
| Vera | Gotz | University Hospitals Of Morecambe Bay NHS Foundation Trust, | Lancaster | United Kingdom |
| Ben | Wooldridge | Warwick Hospital | Warwick | United Kingdom |
| Bridget | Campbell | Warwick Hospital | Warwick | United Kingdom |
| Penny | Parsons | Warwick Hospital | Warwick | United Kingdom |
| Camilla | Stagg | Warwick Hospital | Warwick | United Kingdom |
| Dominika | Dabrowska | West Middlesex University Hospital | London | United Kingdom |
| Omnia | Askar | West Middlesex University Hospital | London | United Kingdom |
| Priyakam | Chowdhury | West Middlesex University Hospital | London | United Kingdom |
| Jamie | Gonzales | West Middlesex University Hospital | London | United Kingdom |
| Swarna | Jeyabraba | West Middlesex University Hospital | London | United Kingdom |
| Angelyn | Sangalang | West Middlesex University Hospital | London | United Kingdom |
| Amrinder | Sayan | West Middlesex University Hospital | London | United Kingdom |
| Surendini | Thayaparan | West Middlesex University Hospital | London | United Kingdom |
| Kaushik | Bhowmick | West Suffolk NHS Foundation Trust | Bury St Edmunds | United Kingdom |
| Sally | Humphreys | West Suffolk NHS Foundation Trust | Bury St Edmunds | United Kingdom |
| Nimal | Mani | West Suffolk NHS Foundation Trust | Bury St Edmunds | United Kingdom |
| Sarah | Pearcey | West Suffolk NHS Foundation Trust | Bury St Edmunds | United Kingdom |
| Shivacharan Patel | Rudrappa | West Suffolk NHS Foundation Trust | Bury St Edmunds | United Kingdom |
| Zi Yi | Tew | West Suffolk NHS Foundation Trust | Bury St Edmunds | United Kingdom |
| Lisa | Jobes | Wrexham Maelor Hospital (BCUHB) | Wrexham | United Kingdom |
| John | Harris | Wrexham Maelor Hospital (BCUHB) | Wrexham | United Kingdom |
| Rachel | Hughes | Wrexham Maelor Hospital (BCUHB) | Wrexham | United Kingdom |
| Emma | Mcivor | Wrexham Maelor Hospital (BCUHB) | Wrexham | United Kingdom |
| Rebecca | Pope | Wrexham Maelor Hospital (BCUHB) | Wrexham | United Kingdom |
| Mary | Roberts | Wrexham Maelor Hospital (BCUHB) | Wrexham | United Kingdom |
| Victoria | Whitehead | Wrexham Maelor Hospital (BCUHB) | Wrexham | United Kingdom |
| Peter | Alexander | Wythenshawe Hospital | Manchester | United Kingdom |
| Sheetal | Crasta | Wythenshawe Hospital | Manchester | United Kingdom |
| Sofia | Fiouni | Wythenshawe Hospital | Manchester | United Kingdom |
| Jane | Shaw | Wythenshawe Hospital | Manchester | United Kingdom |
| Luke | Ward | Wythenshawe Hospital | Manchester | United Kingdom |
| Simon | Davies | York And Scarborough Teaching Hospitial NHS Foundation Trust | Scarborough | United Kingdom |
| Harriet | Carter | York And Scarborough Teaching Hospitial NHS Foundation Trust | Scarborough | United Kingdom |
| Zoe | Scott | York And Scarborough Teaching Hospitial NHS Foundation Trust | Scarborough | United Kingdom |
| Anisha Rahmath | Varodan | Buffalo General Medical Center | Buffalo | United States Of America |
| Liudmila | Asaul | Buffalo General Medical Center | Buffalo | United States Of America |
| Konstantin | Balonov | Tufts Medical Center | Boston | United States Of America |
| Ana | Arias | University Of California Davis | Davis | United States Of America |
| Leidy | Rivas | University Of California Davis | Davis | United States Of America |
| Julio | Pineda | University Of California Davis | Davis | United States Of America |
| Neal | Fleming | University Of California Davis | Davis | United States Of America |
| Brittney | Saverimuttu | University Of California Davis | Davis | United States Of America |
| Aubrey | Yao | University Of California Davis | Davis | United States Of America |
| Meredith | Miller | VA Boston Health Care System | Boston | United States Of America |
| Anuradha | Borle | Washington University In St Louis | Washington | United States Of America |
| Omokhaye | Higo | Washington University In St Louis | Washington | Washington |
| Muthuraj | Kanakaraj | Washington University In St Louis | Washington | Washington |
| **Additional participating institution:** | | | | |
|  | | Complejo Hospitalario Universitario Insular Materno Infantil Las Palmas de Gran Canaria | Las Palmas de Gran Canaria | Spain |
|  | | University of Campania "L.Vanvitelli" | Naples | Italy |
|  | | Sunshine Coast University Hospital | Birtinya | Australia |
|  | | Al Bashir Hospital | Amman | Jordan |
| **ESAIC Management Team** | | | | |
| Sylvia | Daamen | European Society of Anaesthesiology and Intensive Care | Brussels | Belgium |
| Sophie | Debouche | European Society of Anaesthesiology and Intensive Care | Brussels | Belgium |
| Slama | Farsi | European Society of Anaesthesiology and Intensive Care | Brussels | Belgium |
| Pierre | Harlet | European Society of Anaesthesiology and Intensive Care | Brussels | Belgium |
| Flavia | Pirovano | European Society of Anaesthesiology and Intensive Care | Brussels | Belgium |

# **Endorsements of Specialist and National Anesthesia Associations**

Australian and New Zealand College of Anaesthetists (ANZCA)

Association of Anesthesiologists and Intensivists of Serbia

Association of Anaesthesiologists of Malta

Association of Anaesthesiologists-Reanimatologists of Latvia

Belgian Society of Anesthesia and Resuscitation (BSAR)

Brazilian Society of Anesthesiology (SBA)

Czech Society of Anaesthesiology and Intensive Care Medicine (CSARIM)

Dutch Society of Anaesthesiology (NVA)

European Society of Anaesthesiology and Intensive Care (ESAIC)(Sponsor)

European Society of Intensive Care Medicine (ESICM)

French Society of anesthesia, critical care and perioperative medicine (SFAR)

German Society of Anaesthesiology and Intensive Care Medicine (DGAI)

Italian Society of Anaesthesia, Analgesia, Resuscitation and Intensive Care (SIAARTI)

Portuguese Society of Anaesthesiology (SPA)

Russian Federation National Society of Anesthesiologists and Reanimatologists (FAR)

Slovenian Society of Anaesthesiology and Intensive Care (SSAICM)

Slovak Society of Anesthesiology and Intensive Medicine

Spanish Society of Anaesthesia, Reanimation and Pain Management (SEDAR)

Society of Anaesthesia and Reanimatology of the Republic of Moldova

Ukrainian Society of Anaesthesiologists

Support:

American Society of Anaesthesiologists (ASA)

# **Methods**

## **Method S1: In- and exclusion criteria**

We recruited two cohorts of patients.

**Cohort A** include all patients admitted to participating hospitals during seven consecutive days with the following inclusion and exclusion criteria:

| Inclusion criteria | Exclusion criteria |
| --- | --- |
| 1. Undergoing surgery (may be planned or unplanned) 2. No plans for return home on the day of surgery. (No day case surgery) 3. Age ≥ 18 on day of surgery | 1. Cardiac surgery 2. Obstetric surgery 3. Transplant surgery 4. Preoperatively long-term infusions of vasoactive drugs, such as epoprostenol (prostacyclin) 5. Mechanical circulatory support: ventricular assist device, intra-aortic balloon pump, artificial heart or similar 6. Already been enrolled in SQUEEZE |

###

**Cohort B** include 30 sequential patients with **a single additional inclusion** criterion:

| Inclusion criteria | Exclusion criteria |
| --- | --- |
| 1. Postoperative Vasopressor Infusion (PVI) – as defined below. | 1. Already been enrolled in SQUEEZE |

## **Method S2: Flow chart for patient recruitment**


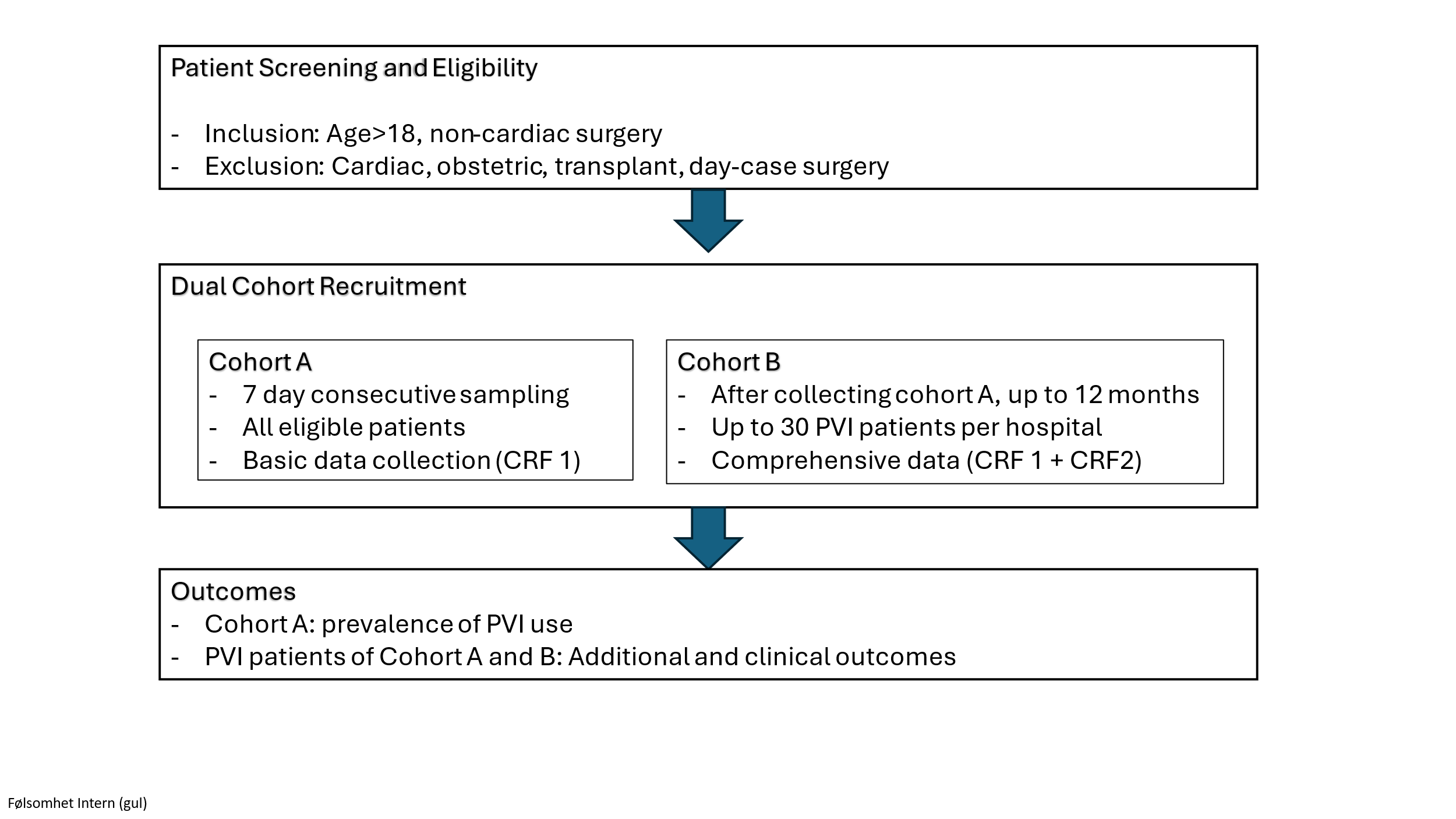


## **Method S3: Definition of Postoperative Vasopressor Infusion (PVI)**

Postoperative Vasopressor Infusion (PVI) is defined, for the purposes of this study, as the continuous intravenous infusion of a drug with a predominant vasoconstrictor effect (vasopressor). Therefore, repeated dosing of intravenous boluses is excluded, and infusion of a drug that is predominantly a positive inotrope (without concurrent vasopressor) is excluded. Additionally, we are not interested in vasopressor infusions that are used intra-operatively to counter the effect of general anaesthesia (or regional anaesthesia) and because this effect can take time to resolve, any infusion of vasopressor in the first hour following surgery is excluded – unless it continues after one hour following surgery. Infusions of vasopressor that are started more than 24 hours after the end of surgery is also excluded from this definition. Infusions of vasopressor that start before surgery will only be included if they also meet the above criteria.

**Classification of vasoactive drugs, grouped according to predominant action. We accept that many drugs have mixed actions.**

| Vasopressor | Not predominantly vasopressor |
| --- | --- |
| - Dopamine - Epinephrine (Adrenaline) - Metaraminol - Norepinephrine (Noradrenaline) - Phenylephrine - Vasopressin or Terlipressin - Akrinor® - Angiotensin II | - Atropine - Dobutamine - Ephedrine - Etilefrine - Glycopyrronnium - Nitrates - Milrinone |

## **Method S4: Case Report form**

| **CRF 1** | |  |  | |  | | |
| --- | --- | --- | --- | --- | --- | --- | --- |
| **0. Informed consent** | |  |  | |  | | |
| Is consent applicable in your centre ? *Mandatory unless the centre has an explicit and written exemption from IRB* | | ○ No    ○ Yes |  | | If yes date of consent: | DD-MMM-YY | |
| **1. Patient Information** | |  |  | |  | | |
| 1.1. Year of  Birth * |  | 1.2.  Weight * |  | | 1.3. Height  * |  | |
| 1.4. Clinical Frailty Scale * | | 1 Very fit |  | ⃝ | 6 Moderately Frail | | ⃝ |
|  |  | 2 Well |  | ⃝ | 7 Severely Frail | | ⃝ |
|  |  | 3 Managing | well | ⃝ | 8 Very severely Frail | | ⃝ |
|  |  | 4 Vulnerabl | e | ⃝ | 9 Terminally | | ⃝ |
|  |  | 5 Mildly Fra | il | ⃝ | 10 Don’t know | | ⃝ |

| **Previous medical history**  * |  |  | | | | |
| --- | --- | --- | --- | --- | --- | --- |
| 1.5. Coronary Artery Disease | ○ No |  | | ○ Yes | | |
| 1.6. Cerebrovascular Disease | ○ No |  | | ○ Yes | | |
| 1.7. Peripheral vascular Disease | ○ No |  | | ○ Yes | | |
| 1.8. Atrial fibrillation | ○ No |  | | ○ Yes | | |
| 1.9. Heart failure | ○ No |  | | ○ Yes | | |
| 1.10. Hypertension | ○ No |  | | ○ Yes | | |
| 1.11. Diabetes | ○ No | ○ Insulin dependent | | | ○ Non-insulin dependent | |
| 1.12. Chronic liver disease | ○ No |  | | ○ Yes | | |
| 1.13. Chronic respiratory disease | ○ No |  | ○ COPD | | | ○ Other |
| 1.14. Long-term steroid use | ○ No |  | | ○ Yes | | |

| **1.15. Regular medications** (tick all that apply, leave blank if not a regular medication) | | | |  |
| --- | --- | --- | --- | --- |
| ACE inhibitor | If  yes  → | ○ took day of surgery | ○ omitted day of surgery | ○ unknown |
| Alpha blocker | If  yes  → | ○ took day of surgery | ○ omitted day of surgery | ○ unknown |
| Angiotensin receptor blocker | If  yes  → | ○ took day of surgery | ○ omitted day of surgery | ○ unknown |
| Beta blocker | If  yes  → | ○ took day of surgery | ○ omitted day of surgery | ○ unknown |
| Calcium channel blocker | If  yes  → | ○ took day of surgery | ○ omitted day of surgery | ○ unknown |
| Diuretic | If  yes  → | ○ took day of surgery | ○ omitted day of surgery | ○ unknown |
| Regular NSAIDs | If  yes  → | ○ took day of surgery | ○ omitted day of surgery | ○ unknown |

| **Haemodynamics.** Leave blank if not available | | | |
| --- | --- | --- | --- |
| Measurements in the past 6 months or at least 12h prior to the operating room, at rest. | | | |
| 1.16. Systolic: | 1.17 Diastolic: | | 1.18 Heart rate: |
| Reading immediately prior to induction of anaesthesia: | | | |
| 1.19. Systolic: 1.20 Diastolic: 1.21 Heart rate: | | | |
| **Laboratory.** Leave blank if not available, indicate which unit | | | |
| 1.22. Creatinine: |  | mg/dl or µmol/L | |
| 1.23. Albumin |  | g/dL, g/L or µmol/L | |
| 1.24. Haemoglobin |  | g/dL, g/L or mmol/L | |

| 2. **Surgery** |  | |  | | | |
| --- | --- | --- | --- | --- | --- | --- |
| 2.1 Reason for surgery * | Infection | | ⃝ | | | |
|  | Cancer | | ⃝ | | | |
| 2.2 Surgical procedure  *  (select single most appropriate) | | Breast | | ⃝ | Orthopaedic | ⃝ |
|  |  | Gynaecological | | ⃝ | Plastics / Cutaneous | ⃝ |
|  |  | Head and neck | | ⃝ | Upper gastro-intestinal | ⃝ |
|  |  | Hepato-biliary | | ⃝ | Neurological/spinal | ⃝ |
|  |  | Kidney / urological | | ⃝ | Vascular | ⃝ |
|  |  | Lower gastro-intestinal | | ⃝ | Other | ⃝ |
| 2.3 Severity  * | | Minor | | ⃝ |  |  |
|  |  | Intermediate | | ⃝ |  |  |
|  |  | Major | | ⃝ |  |  |
| 2.4. ASA-PS:  * | | ASA 1: Healthy person | | | | ⃝ |
|  |  | ASA 2: Mild systemic disease. | | | | ⃝ |
|  |  | ASA 3: Severe systemic disease | | | | ⃝ |
|  |  | ASA 4 Severe systemic disease that is a constant threat to life. | | | | ⃝ |
|  |  | ASA 5 A moribund person who is not expected to survive without the operation. | | | | ⃝ |
| 2.5. Urgency  * | | Urgent  (includes emergency, expedited, urgent and immediate) | | | | ⃝ |
|  |  | Not urgent  (includes planned/elective) | | | | ⃝ |

|  | Fracture | ⃝ |
| --- | --- | --- |
|  | Bleeding | ⃝ |
|  | Other | ⃝ |

| **3. Operative** | | | | | |  |
| --- | --- | --- | --- | --- | --- | --- |
| 3.1. Date of anaesthesia induction * | **DD-MMM-YY** | | | | |  |
| 3.2. Time of anaesthesia induction * | **HH:MM** | | | | |  |
| 3.3. Date of end of surgery * | **DD-MMM-YY** | | | | |  |
| 3.4. Time of end of surgery * | **HH:MM** | | | | |  |
| 3.5. Estimated blood loss (mL) * | <250 ○ | 251-1000 ○ | 1001-3000 ○ | |  | >3000 ○ |
| 3.6 /3.7 Lowest intraoperative blood pressure (paired) * | Systolic: | | Diastolic: | | |  |
| 3.8. Anaesthesia: *  (Tick all that apply) | Volatile | | |  | |  |
|  | TIVA | | |  | |  |
|  | Sedation without securing airway | | |  | |  |
|  | Regional | | |  | |  |
|  | Spinal | | |  | |  |
|  | Epidural | | |  | |  |
| 3.9. Airway  * | Endotracheal tube | | | ⃝ | |  |
|  | Supraglottic airway | | | ⃝ | |  |
|  | O2 facemask or nasal cannula | | | ⃝ | |  |
| 3.10. Arterial line * | ○ No | | ○ Yes | | |  |
| 3.11. Central venous line * | ○ No | | ○ Yes | | |  |
| 3.12. Which Intra-operative vasoactive drugs    [Tick all that apply] | Atropine | | |  | |  |
|  | Akrinor ® (Cafedrin/Theodrenalin) | | |  | |  |
|  | Dobutamine | | |  | |  |
|  | Dopamine | | |  | |  |
|  | Ephedrine | | |  | |  |
|  | Epinephrine (Adrenaline) | | |  | |  |
|  | Glycopyrronnium | | |  | |  |
|  | Metaraminol | | |  | |  |

|  | | | Milrinone | | | | | | | | | |  |
| --- | --- | --- | --- | --- | --- | --- | --- | --- | --- | --- | --- | --- | --- |
|  |  |  | Nitrates | | | | | | | | | |  |
|  |  |  | Norepinephrine (Noradrenaline) | | | | | | | | | |  |
|  |  |  | Phenylephrine | | | | | | | | | |  |
|  |  |  | Vasopressin or Terlipressin | | | | | | | | | |  |
| 3.13. Was the patient receiving a vasopressor infusion prior to anaesthesia? * | | | | | | | | | | ○ No | | | ○ Yes |
| 3.14. Fluids and blood products received during surgery: | | | Crystalloid | | | | | | |  | | | (mL) |
|  |  |  | Colloid  (starch-gelofusine-albumin) | | | | | | |  | | | (mL) |
|  |  |  | Packed red blood cells | | | | | | |  | | | (mL) |
|  |  |  | Fresh frozen plasma | | | | | | |  | | | (mL) |
|  |  |  | Platelets | | | | | | |  | | | (mL) |
|  |  |  | Whole blood or autotransfusion | | | | | | |  | | | (mL) |
| **4. Post-operative**  Following the end of surgery (within 24h): | | | | | | | | | | |  | | |
| 4.1. Did the patient receive enteral vasopressors?  (i.e. MIDODRINE) * | | | | | | ○ No | | | | | ○ Yes | | |
| 4.2. Did the patient receive **boluses** of vasopressors? * | | | | | | ○ No | | | | | ○ Yes | | |
| 4.3. Did the patient receive an **infusion** of vasopressors? * | | | | | | ○ No (Stop here) | | | | | ○ Yes (Continue with  4.3.1 and 4.3.2) | | |
| 4.3.1. **If yes**, did the infusion continue or start after 1 hour from the end of surgery? | | | | | | ○ No | | | | | **○ Yes** | | |
| 4.3.2. **If Yes**, Did this infusion start within 24 hours from the end of surgery? | | | | | | ○ No | | | | | **○ Yes** | | |
| **If “yes” on 4.3.1 and .4.3.2, complete CRF2** | | | | | | | | | | |  | | |
| **5. Outcomes** | | | | | | | | | | | | | |
| 5.1. Ventilation:  * | ○ No | | ○ Invasive mechanical ventilation (IMV) | | | | | ○ Non Invasive Ventilation (NIV) | | | | | |
| 5.2. Acute Myocardial Infarction * | | | ○ No | | | | | ○ Yes | | | | | |
| 5.3. New onset atrial fibrillation * | | | ○ No | | | | | ○ Yes | | | | | |
| 5.4. New onset other dysrhythmia * | | | ○ No | | | | | ○ Yes | | | | | |
| 5.5. Renal: Highest creatinine  (within the first week) postoperatively | | | |  | | | | mg/dl or µmol/L indicate which unit | | | | | |
| 5.6. Renal replacement therapy * | | | ○ No | | | | | ○ Yes | | | | | |
| 5.7. Parenteral nutrition * | | | ○ No | | | | | ○ Yes | | | | | |
| 5.8. Antibiotics for a newly diagnosed infection * | ○ No | | | | ○ Yes (complete ) | | | | | | | ○ Unknown | |
|  | ○ Skin or soft tissue  ○ Respiratory  ○ Urinary | | | | | | ○ Abdominal  ○ Lines  ○ Other | | | | | | |
| 5.9. Accordion classification of surgical  complication * | ○ None | | | | | | | | | | | | |
|  | ○ Mild complication | | | | | | | | | | | | |
|  | ○ Moderate complication | | | | | | | | | | | | |
|  | ○ Severe complication | | | | | | | | | | | | |
|  | ○ Death |  | | | | | | |  | | | | |
| 5.10. Date of hospital discharge **or** date of intrahospital death: * | |  | | | | | | | DD-MMM-YY | | | | |
| 5.11. Stayed an inpatient for more than 30 days? * | | ○ No | | | | | | | ○ Yes | | | | |


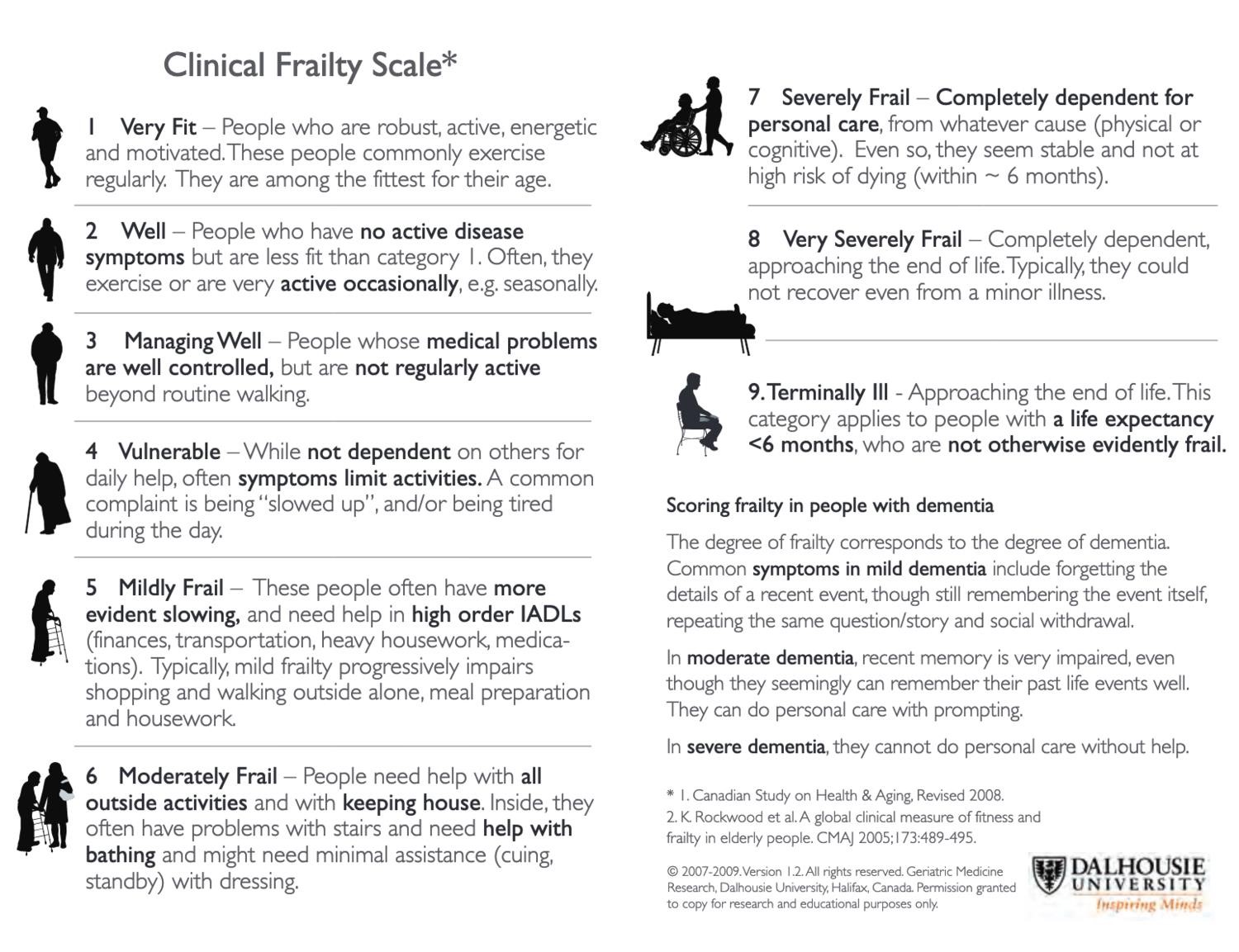


2.3. Severity of surgery:

Minor: Procedure < 30 minutes. Examples: arthroscopy without intervention, removal of cutaneous tumour, proctology procedures, biopsy or excision biopsy of small lesions, etc

Intermediate: Procedure performed in a dedicated operating room that may pose the risk of significant complications or tissue injury. Examples: laparoscopic cholecystectomy, arthroscopy with intervention, bilateral varicose vein removal, tonsillectomy, inguinal hernia repair, breast lump resection, haemorrhoidectomy, appendicectomy, partial thyroidectomy, cataract surgery, uvuloplasty, minimally invasive repair of vaginal prolapse, vaginal hysterectomy, fixation of mandibular fracture, etc

Major: Performed in a dedicated operating room and is expected to last more than 90 minutes. Examples: major gut resection, major joint replacement, mastectomy, extensive head and neck tumour resection, abdominal aortic aneurysm repair, major vascular bypass procedure, procedures involving free flap to repair tissue defect, amputation, total thyroidectomy, cystectomy, trans-urethral resection of prostate, resection of liver tumour, carotid endarterectomy, nephrectomy, total abdominal hysterectomy, spinal discectomy, etc

| At one hour after the completion of surgery, is the patient: | |  |
| --- | --- | --- |
| 6.2. Receiving continuous infusion of neuraxial anaesthesia/analgesia i.e. epidural infusion * | ○ No | ○ Yes |
| 6.3. Still receiving a sedative infusion * | ○ No | ○ Yes |
| 6.4. Still has an airway in place (endotracheal tube, tracheostomy or supraglottic airway) * | ○ No | ○ Yes |

| **CRF 2** |  |  |
| --- | --- | --- |
| **Postoperative vasopressor infusion** |  |  |
| 6.1. Did this patient have an **infusion** of vasopressors that was either started or continued at least 1 hour after surgery: * | ○ No    (If ‘No’ then please do not complete any further) | ○ Yes |

| 6.5. How was it assessed that this patient should receive a vasopressor infusion? * | | |
| --- | --- | --- |
|  | ○ Already receiving a vasopressor infusion and attempts to lower the infusion rate produced unacceptable hypotension, OR | |
|  | ○ It was decided that the patient would no longer benefit from further attempts to increase the cardiac output through administration of IV fluids and the blood pressure was unacceptably low. This was on the basis of: | |
|  |  | ○ A. Clinical assessment alone (vital signs-examination-lab results) |
|  |  | ○ B. Clinical assessment AND a measurement of preload responsiveness using cardiac output monitoring (or some direct surrogate of) |
|  |  | ○ C. Clinical assessment AND a measurement of preload responsiveness using echocardiography |
|  |  | ○ D. Clinical assessment AND a previously established maximum for IV fluid administration has been met i.e. 2L or 20ml/kg etc... |
|  |  | ○ E. other: |
|  |  |  |

| 7.1. SOFA score within 24 hours after surgery * [0-24] |  | (Use FAQ as required) To calculate SOFA score:  <https://clincalc.com/IcuMortality/SOFA.aspx> |
| --- | --- | --- |

| 7.2 - 7.8 MAP target (complete only if MAP is specified) | | | | |  |  |  |
| --- | --- | --- | --- | --- | --- | --- | --- |
|  | Day 0 | Day 1 | Day 2 | Day 3 | Day 4 | Day 5 | Day 6 |
| MAP |  |  |  |  |  |  |  |

| 7.10 – 7.16 **HIGHEST** blood pressure for each day (paired)  On Postoperative unit/ICU only. Leave blank if not available. | | | | |  |  |  |
| --- | --- | --- | --- | --- | --- | --- | --- |
|  | Day 0 | Day 1 | Day 2 | Day 3 | Day 4 | Day 5 | Day 6 |
| Systolic |  |  |  |  |  |  |  |
| Diastolic |  |  |  |  |  |  |  |

| 7.18 – 7.24 **LOWEST** blood pressure during the day (paired)  On Postoperative unit/ICU only. Leave blank if not available. | | | | |  |  |  |
| --- | --- | --- | --- | --- | --- | --- | --- |
|  | Day 0 | Day 1 | Day 2 | Day 3 | Day 4 | Day 5 | Day 6 |
| Systolic |  |  |  |  |  |  |  |
| Diastolic |  |  |  |  |  |  |  |

| 7.26 – 7.32 **Vasoactive drug infusion, tick if applicable** | | | | |  |  |  |
| --- | --- | --- | --- | --- | --- | --- | --- |
|  | Day 0 | Day 1 | Day 2 | Day 3 | Day 4 | Day 5 | Day 6 |
| Noradrenaline |  |  |  |  |  |  |  |
| Angiotensin II |  |  |  |  |  |  |  |
| Dobutamine |  |  |  |  |  |  |  |
| Dopamine |  |  |  |  |  |  |  |
| Epinephrine (Adrenaline) |  |  |  |  |  |  |  |
| Metaraminol |  |  |  |  |  |  |  |
| Milrinone |  |  |  |  |  |  |  |
| Phenylephrine |  |  |  |  |  |  |  |
| Terlipressin |  |  |  |  |  |  |  |
| Vasopressin |  |  |  |  |  |  |  |

| Total number of days: * | |
| --- | --- |
| 7.33. receipt of ventilation (invasive or NIV) |  |
| 7.34. receipt of vasopressor infusion |  |
| 7.35. receipt of parenteral nutrition |  |
| 7.36. receipt of renal replacement therapy |  |
| 7.37. duration of stay in ICU/postoperative unit |  |

| COVID | |  |  | |  |
| --- | --- | --- | --- | --- | --- |
| 7.38. Did the patient have any testing for SARS-CoV2? * | ○ No |  | ○ Yes  **If yes, answer below** | | ○ Unknown |
| 7.38.1. **If Yes -** did the patient test positive in the perioperative period? | | ○ No |  | ○ Yes |  |

## **Method S5: Definitions of variables and instructions to investigators**

1.1. Year of birth (mandatory)

It is not necessary for us to know a date of birth, just the year of birth.

1.2./1.3. Weight and Height (mandatory)

Estimated if necessary.

1.4. Clinical Frailty Scale

This is easiest to do after reading clinical notes and having a brief chat with the patient about their lives. It does not need to be done by a specialist like a geriatrician or occupational therapist. If it is impossible to find out this information then there is an option for “don’t know”.

1.5.-1.15. Previous medical history (mandatory)

A series of nine Yes/No questions about co-morbid conditions. We are not providing definitions these can be previously confirmed diagnoses or concluded from available data.

Chronic liver disease should include conditions characterised by impairment of liver function, or a significant predisposition to failure of liver function. Cirrhosis of any extent would be a ‘Yes’. A single hepatic metastasis would be ‘No’, whereas a large number of metastases without significant remaining liver would be a ‘Yes’.

COPD is Chronic Obstructive Pulmonary Disease and includes emphysema and chronic bronchitis.

Eight questions about chronic medication use, for seven of them if they are selected then a further question is asked to determine if the medication was taken on the day of surgery or not.

The medication questions are about drug classes. If you’re unsure of the class of the medications the patient is taking, then please google it! A low dose aspirin (75mg, for example) does not count as an NSAID. Inhaled corticosteroids do not count as long-term steroid use.

Ideally we’d like to know if the medication was taken on the day of surgery but if it’s impossible to know this then it can be indicated.

1.16.-1.21. Haemodynamics

1.16-1.18. Recent blood pressure and heart rate, if available. If not available, leave blank.

1.19.-1.21. Blood pressure and heart rate immediately before anaesthesia, should be present in most cases.

1.22.-1.24. Laboratory

Creatinine, Albumin and Haemoglobin concentration, if available. Use values closest to time of anaesthesia.

2.1.-2.2 Reason for surgery and category of surgical procedure.

Please choose the one that fits best.

We will analyse the data using the reason for surgery and the type of surgery. Please do not avoid selecting a reason or category in order to enter text into a free text box or discrepancy note. It makes a lot of work for the team and as we won’t be using the entered data it’s a waste of your time!

For example, for laparoscopic cholecystectomy being done for episodes of cholecystitis – please select “infection” and “upper GI surgery”. Do not select “other” and “other” in order to tell us that it was a laparoscopic cholecystectomy.

2.3. Severity of surgery (minor/intermediate/major).

Please choose the one that fits best and use common sense.

- - Minor: Procedure of less than 30 minutes duration performed in a dedicated operating room which would often involve extremities or body surface or brief diagnostic and therapeutic procedures. Examples include: arthroscopy without intervention, removal of small cutaneous tumour, diagnostic proctology procedures, biopsy or excision biopsy of small lesions, etc
  - Intermediate: More prolonged or complex procedure performed in a dedicated operating room that may pose the risk of significant complications or tissue injury. Examples include: laparoscopic cholecystectomy, arthroscopy with intervention, bilateral varicose vein removal, tonsillectomy, inguinal hernia repair, breast lump resection, haemorrhoidectomy, appendicectomy, partial thyroidectomy, cataract surgery, uvuloplasty, minimally invasive repair of vaginal prolapse, vaginal hysterectomy, tendon repair of hand, fixation of mandibular fracture, etc
  - Major: Any surgical procedure that requires anaesthesia, performed in a dedicated operating room and is expected to last more than 90 minutes. Examples include: major gut resection, major joint replacement, mastectomy, extensive head and neck tumour resection, abdominal aortic aneurysm repair, major vascular bypass procedure, procedures involving free flap to repair tissue defect, amputation, total thyroidectomy, cystectomy, trans-urethral resection of prostate, resection of liver tumour, carotid endarterectomy, nephrectomy, total abdominal hysterectomy, spinal discectomy, etc

2.4. ASA-PS

‘American Society of Anaesthesiology Physical status’ use the value attributed by the anaesthetist.

We do not include ASA VI in our list since organ donors are not included in SQUEEZE.


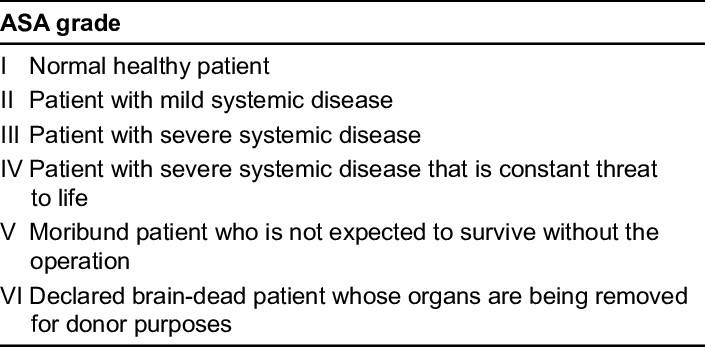


2.5. Urgency

Urgent (includes emergency, expedited, urgent and immediate)

Non-urgent (also known as planned or elective)

3.1.-3.2. Date and time of anaesthesia induction (mandatory)

3.3.-3.4. Date and time of end of the surgery (mandatory)

Different hospitals use different definitions for start and finish of anaesthesia and surgery and we will not force you to use one single definition – please use whatever your hospital uses. The date is necessary for the unusual occurrences of surgeries that span one day to the next.

3.5. Estimated blood loss (mandatory)

3.6. Lowest systolic and lowest diastolic (mandatory)

Values taken at the same time, selected based on the systolic. For example:

|  | 09:05 | 09:10 | 09:15 | 09:20 | 09:25 |
| --- | --- | --- | --- | --- | --- |
| Systolic (SBP) | 120 | 100 | 90 | 85 | 92 |
| Diastolic (DBP) | 80 | 70 | 60 | 65 | 70 |

The lowest SBP is 85, at this time the diastolic is 65 – so these are the values we want.

We do not want SBP 85 and DBP of 60 as these values were not taken at the same time.

3.8. Anaesthesia

More than one type can be selected. TIVA refers to total intravenous anaesthesia but this is not restricted to use of specific pumps or dosing systems.

We are specifically interested in maintenance of anaesthesia not induction. For example, if the induction of anaesthesia is volatile and then TIVA is used for maintenance – please just select TIVA. If induction is with IV and then volatile is used for maintenance – please just select volatile.

Details about the epidural including level of insertion, the height of the block and the drugs given are not required. Equally, details about any spinal are not required.

3.9. Airway

Please check the most appropriate one. More than one can be selected.

3.10. Arterial line

Is there a cannula / catheter in a peripheral artery for the purposes of monitoring?

3.11. Central Venous Line

Is there a cannula / catheter in a central vein? It may be newly sited or already present for monitoring or therapy.

Please exclude peripherally inserted central cannulae (PICC), midlines or long term central venous lines for dialysis, parenteral nutrition or chemotherapy unless they are being used perioperatively for vasopressors.

3.12. Intra-operative drugs via infusion or bolus

This is a list of vasoactive medications that the patient receives during surgery. The dosing is not recorded. Please check all that applies.

Please note that this only relates to drugs given INTRA-operatively.

3.13. Was the patient receiving a vasopressor infusion prior to anaesthesia?

3.14. Fluids and blood products received during surgery

For each of six types of fluids, please enter the volume in millilitres. If the records only indicate how many units of a product, please estimate the volumes based on your local experience.

1. Five questions about post-operative vasopressors:
   1. Yes/No question about post-operative receipt of enteral vasopressors.

4.1 Yes/No question about **post-operative** receipt of **boluses** of vasopressors (different to earlier question about intra-operative).

4.3 Yes/No question about **post-operative** receipt of **infusions** of vasopressors (different to earlier question about intra-operative).

- - 1. Yes/No question about if a post-operative infusion of vasopressors continued for more than 1 hour after the end of surgery.
    2. Yes/No question about if a post-operative infusion of vasopressors started within 24 hours of the end of surgery.

These questions are to determine if the patient in question fulfils the criteria for PVI, which would mean that additional questions (CRF2) need to be completed.


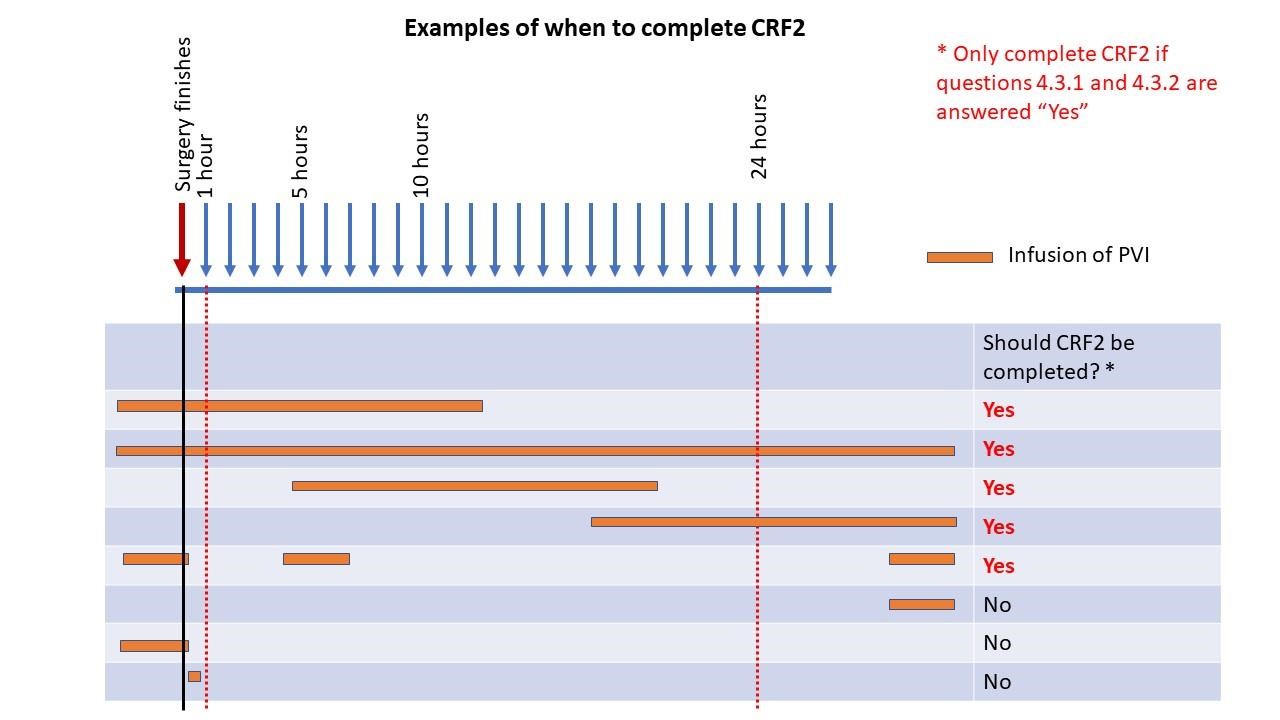


From the protocol:

**Definition**: Postoperative Vasopressor Infusion (PVI) is defined, for the purposes of this study, as the continuous intravenous infusion of a drug with a predominant vasoconstrictor effect (vasopressor). Therefore, repeated dosing of intravenous boluses is excluded, and infusion of a drug that is predominantly a positive inotrope (without concurrent vasopressor) is excluded. Additionally, we are not interested in vasopressor infusions that are used intra-operatively to counter the effect of general anaesthesia (or regional anaesthesia) and because this effect can take time to resolve, any infusion of vasopressor in the first hour following surgery is excluded – unless it continues after one hour following surgery. Infusions of vasopressor that are started more than 24 hours after the end of surgery is also excluded from this definition. Infusions of vasopressor that start before surgery will only be included if they also meet the above criteria.

5.1 . Intrahospital, post-operative complications

During the patient’s 30 days following the date of surgery:

• Ventilation: No, NIV, IMV.

If the patient received invasive mechanical ventilation (IMV, via endotracheal tube or tracheostomy) that started after the end of surgery, then please select this. If the patient *continued to receive* invasive mechanical ventilation that *started prior* to surgery, then please do NOT select this.

On the day of surgery there will often have been invasive mechanical ventilation and if that is completed (i.e. the patient was extubated) within 4 hours of the end of surgery then this would not count as a day of IMV. If IMV continues for more than 4 hours after the end of surgery then this should count as a day of postoperative IMV.

If the patient received non-invasive ventilation (NIV, including BiPAP and CPAP) via a facemask (any duration) then please select this. For the purposes of this study high flow oxygen delivered via nasal cannulae is not considered NIV.

If neither IMV nor NIV are provided then please select No.

5.2 Acute Myocardial Infarction: No/ Yes

If the clinicians believe that the patient has an acute Myocardial Infarction then please select Yes. If you’re not sure (is it just a troponin rise?) then please ask your principal investigator to adjudicate.

5.3 New onset Atrial Fibrillation: No/ Yes

If the clinicians believe that the patient has atrial fibrillation that was not present prior to the operation (i.e. no history of chronic or paroxysmal AF) and is more than briefly present, then please select yes.

5.4. New onset of other dysrhythmia: No/ Yes

If the clinicians believe that patient has any new dysrhythmia (includes SVT, VF and VT) that was not present prior to the operation and is more than briefly present, then please select yes.

5.5. Highest creatinine within the first week.

This will allow us to determine if the patient met criteria for acute kidney injury (AKI). Leave blank if you do not have measured creatinine postoperatively.

5.6 . Renal replacement therapy: No/Yes.

If the patient received at least one episode of renal replacement therapy (including haemodialysis, haemofiltration, haemodiafiltration, peritoneal dialysis) and this isn’t a usual occurrence for them (i.e. they don’t usually require any form of renal replacement therapy, RRT) then please select Yes. It is not important if they received the RRT intermittently or continuously.

If they have chronic RRT or did not receive any RRT then please select No.

5.7 . Parenteral nutrition: No/Yes.

If the patient received at least one bag of parenteral nutrition (PN) and this isn’t a usual occurrence for them (i.e. they don’t have chronic intestinal failure) then please select Yes. If they have chronic intestinal failure or did not receive any PN then please select No.

Parenteral nutrition does not include simple dextrose infusions.

5.8. Antibiotics for a newly diagnosed infection:

If the clinicians believe that patient has an infection and they have started some antibiotics then please select yes. A further selection will appear and please select the most appropriate of: skin (or soft tissue), respiratory, urinary, abdominal, lines, other. During their postoperative recovery they may have multiple infections – please select all that apply.

5.9. Severity of surgical complication

This is the Accordion classification of surgical complication. Choose one of the following:

1. None
2. Mild complication: Requires only minor invasive procedures that can be done at the bedside such as insertion of intravenous lines, urinary catheters, and nasogastric tubes, and drainage of wound infections. Physiotherapy and the following drugs are allowed-antiemetics, antipyretics, analgesics, diuretics, electrolytes, and physiotherapy.
3. Moderate complication: Requires pharmacologic treatment with drugs other than such allowed for minor complications, for instance antibiotics. Blood transfusions and total parenteral nutrition are also included.
4. Severe complication: All complications requiring endoscopic or interventional radiologic procedures or re-operation as well as complications resulting in failure of one or more organ systems.
5. Death

5.10. Survival to hospital discharge: Yes or No (mandatory)

All patients are to be followed up either until discharge or for a maximum of 30 days if they stay in hospital.

For the item 5.10. Survival to hospital discharge:

- If the answer is Yes the date of discharge is shown
- If the answer is No the date of death is shown

If the patient is still in the hospital you do not have additional option to indicate this status. In this case, please use the flag to enter a note and ignore question 5.10, and complete item 5.11 and save.

How to use the flag on item 5.10 :


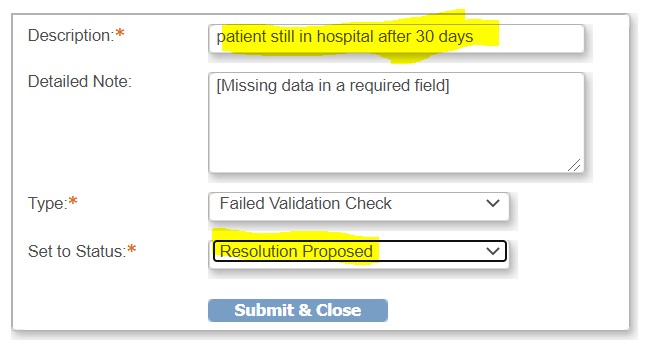


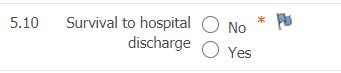


**Please monitor the patient’s status until day 30 or hospital discharge**. If they are alive at day 30 but die on day 31 (or later) then, for the purposes of this CRF and study, their 30-day mortality status is alive.

6.1.-6.5. The first question aims to double-check that this form is being completed only in appropriate patients. (mandatory)

Then there are three questions that aim to further characterise the patient with PVI: (mandatory)

- - Are they receiving a continuous infusion of neuraxial anaesthesia/analgesia i.e. epidural infusion: Yes/No.
  - Are they receiving a continuous infusion of sedative drug i.e. propofol or midazolam or similar: Yes/No.
  - Does the patient have an airway in place (endotracheal tube, tracheostomy or supraglottic airway): Yes/No.

6.6. HOW was it determined that the patient should be receiving PVI. (Mandatory)

The investigator needs to determine how the clinical team decided to use a PVI. There is a choice of two options:

Either “Already receiving a vasopressor infusion and attempts to lower the infusion rate produced unacceptable hypotension”

Or “It was decided that the patient would no longer benefit from further attempts to increase the cardiac output through administration of IV fluids and the blood pressure was unacceptably low.”

If the second choice is selected that the investigator must choose an option that helps us understand why this was decided, one of the following options must be chosen:

- Clinical assessment alone (vital signs-examination-lab results)
- Clinical assessment AND a measurement of preload responsiveness using cardiac output monitoring (or some direct surrogate of)
- Clinical assessment AND a measurement of preload responsiveness using echocardiography
- Clinical assessment AND a previously established maximum for IV fluid administration has been met i.e. 2L or 20ml/kg etc...
- other
- unknown

It may be difficult to determine this solely from the documentation and we would like to avoid too many patients where “other” or “unknown” is selected as it’s not useful information. Please talk to your clinicians and politely enquire which of the options is most suitable.

7.1 SOFA score

This is the sequential organ failure score. It is widely used in critical care and can simply be determined. There is a link to an online calculator.

We are interested in the *highest* score in the first 24 hours after surgery.

Calculating the SOFA score

Healthy person scores 0

Maximally sick person scores 24

**Respiratory**

If an Arterial Blood Gas is available then please use the values taken at the same time for PaO2

(partial pressure of oxygen in arterial blood) and FiO2 (fraction of inspired oxygen 0.21 = 21% = air)

| **PaO_2_/FiO_2_ (kPa)** | **SOFA score** |
| --- | --- |
| ≥ 53.3 | 0 |
| < 53.3 | +1 |
| < 40 | +2 |
| < 26.7 **and** mechanically ventilated | +3 |
| < 13.3 **and** mechanically ventilated | +4 |

If Arterial Blood gases have NOT been done in the 6 hours prior to enrolment, then use the values taken at the same time for SpO2 (Saturations of oxygen in arterial blood, from pulse oximetry) and

FiO2 (fraction of inspired oxygen 0.21 = 21% = air)

| **SpO_2_/FiO_2_** | **SOFA score** |
| --- | --- |
| ≥ 512 | 0 |
| < 512 | +1 |
| < 357 | +2 |
| < 214 | +3 |
| < 89 | +4 |

Reference: <https://www.ncbi.nlm.nih.gov/pmc/articles/PMC3776410/>

**Nervous system**

| **Glasgow coma scale** | **SOFA score** |
| --- | --- |
| 15 | 0 |
| 13–14 (delirium) | +1 |
| 10–12 (obtunded) | +2 |
| 6–9 (semi-comatose) | +3 |
| < 6 (comatose) | +4 |

**Cardiovascular**

If MAP hasn’t been recorded or charted by Systolic and Diastolic have been, then calculate the MAP using this formula: MAP = 1/3 (SBP – DBP) + DBP [norepinephrine](https://en.wikipedia.org/wiki/Norepinephrine) = noradrenaline

| **Mean arterial pressure OR administration of vasopressors required** | **SOFA score** |
| --- | --- |
| MAP ≥ 70 mmHg | 0 |
| MAP < 70 mmHg | +1 |
| dopamine ≤ 5 μg/kg/min or dobutamine (any dose) | +2 |
| dopamine > 5 μg/kg/min OR [epinephrine](https://en.wikipedia.org/wiki/Epinephrine) ≤ 0.1 μg/kg/min OR [norepinephrine](https://en.wikipedia.org/wiki/Norepinephrine) ≤ 0.1 μg/kg/min | +3 |
| dopamine > 15 μg/kg/min OR epinephrine > 0.1 μg/kg/min OR norepinephrine > 0.1 μg/kg/min | +4 |

NB For converting different vasopressors into norepinephrine equivalents (all in mcg/kg/min, except vasopressin in units/min): NE = norepinephrine + epinephrine + phenylephrine/10 + dopamine/100 + metaraminol/8 + vasopressin*2.5 + angiotensin II*10 (REF https://doi.org/10.1016/j.jcrc.2020.11.002)

**Liver**

| **Bilirubin μmol/L** | **SOFA score** |
| --- | --- |
| < 20, or not measured | 0 |
| 20-32 | +1 |
| 33-101 | +2 |
| 102-204 | +3 |
| > 204 | +4 |

**Coagulation**

| **Platelets ×10^3^/μl** | **SOFA score** |
| --- | --- |
| ≥ 150, or not measured | 0 |
| < 150 | +1 |
| < 100 | +2 |
| < 50 | +3 |
| < 20 | +4 |

**Renal**

| **Creatinine μmol/L (or urine output)** | **SOFA score** |
| --- | --- |
| < 110 | 0 |
| 110-170 | +1 |
| 171-299 | +2 |
| 300-440 or < 500 ml/d | +3 |
| > 440 or < 200 ml/d | +4 |

7.2 -7.8 MAP target

Typically, in patients receiving PVI there is a target blood pressure and most commonly it is a target for the mean arterial pressure (MAP).

We are interested in the MAP target for each of day 0, 1, 2, 3, 4, 5 and 6.

If the MAP target is documented as a range, i.e. 65-70mmHg then please use the lower number (65mmHg in this case).

If it is unknown then this can be indicated.

7.9-7.24 Blood pressure

For each day we would you to identify the highest and the lowest paired BP (systolic and diastolic) during that calendar day. Please leave blank if you have no available data.

7.25-7.32 Vasoactive drug infusions = vasopressors and/or inotropes

For each day we would you to indicate if the patient is receiving any amount of each of the vasoactive drugs as an infusion.

7.33-7.37 Outcomes

In the first 30 days following surgery, how many days (in total, not necessarily serially) was there:

1. Receipt of ventilation (IMV or NIV)
2. Vasopressor infusion
3. Parenteral nutrition
4. Renal replacement therapy
5. Time spent on the ICU/HDU/PACU.

The definitions for these are unchanged from earlier.

7.38 COVID questions

Testing for the presence of virus, **not** antibodies to the virus.

The perioperative period is considered to be one week before surgery and anytime during the hospital stay after.

they also need CRF2 completed about them.

## **Method S6: Squeeze analysis data set selection**


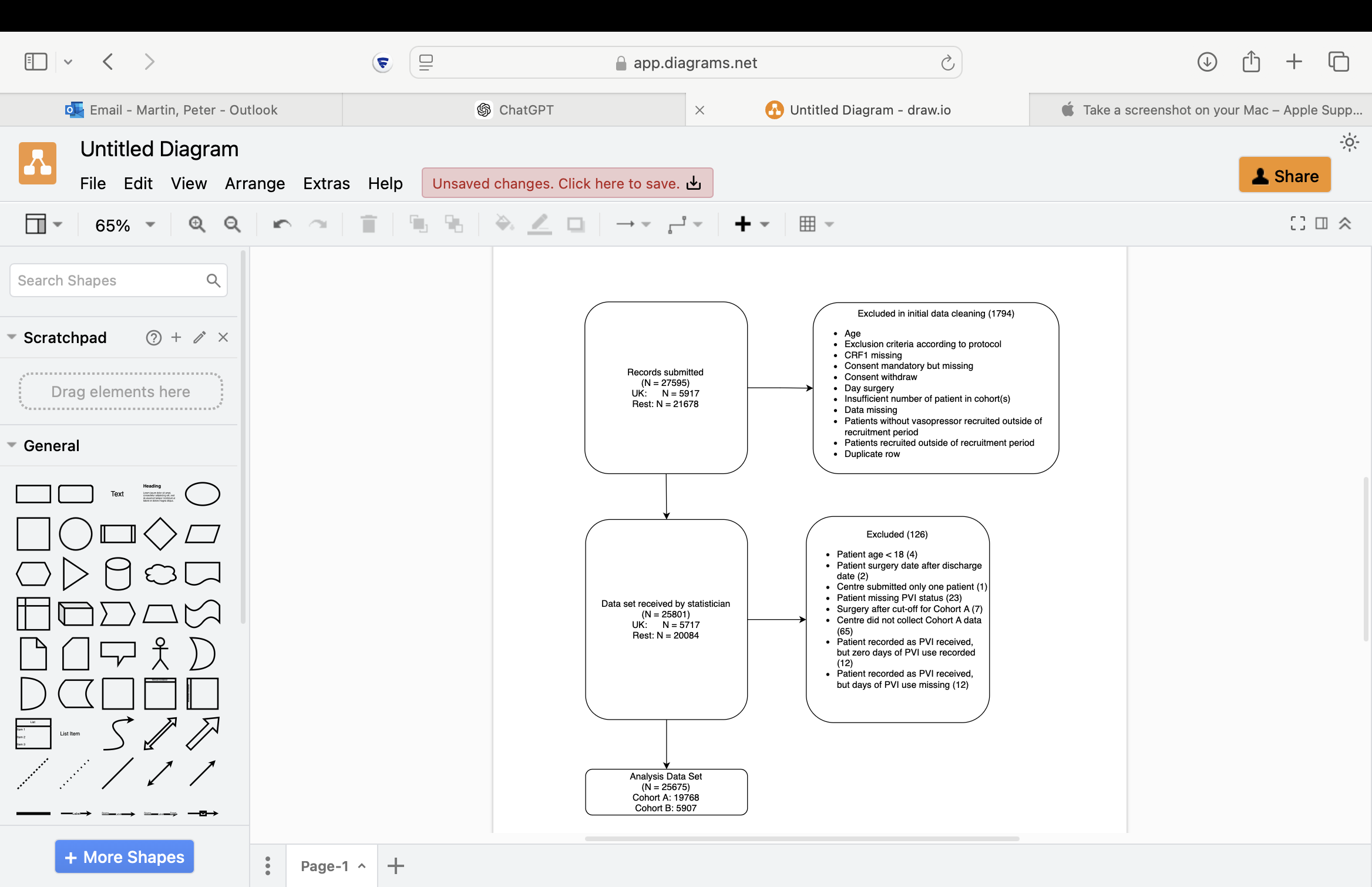


Flow diagram of the data set selection.

## **Method S7: Statistical data analysis: methodology**

A statistical analysis plan (SAP) was published alongside the study protocol prior to completion of data collection. The analysis that was carried out deviates from the SAP in some respects. These deviations are listed, summarized and justified in detail in the Table below. There were two broad reasons for these deviations:

1. Initial exploratory analysis of the data revealed considerable variation in rates of PVI use between countries and between hospitals in the same country. The modelling of this variation became a more important aim of the analysis than had been envisaged during the planning of the study. In particular, it was important to establish whether patient characteristics could explain some or all of the variation in PVI use rates between countries and hospitals. Thus it was decided, contrary to the SAP, to use only Cohort A in analyses relating characteristics of the patient and surgical procedure to PVI use as an outcome. Inclusion of Cohort B in this analysis would have made it impossible to derive estimates of between-country or between-hospital variation, as all patients in Cohort B were in receipt of PVI by definition.
2. We encountered considerable sparsity in the data: 67 out of 228 hospitals reported no patient with PVI use in Cohort A. This made complex three-level models (patients clustered within hospitals, clustered within countries) impossible to estimate using restricted maximum likelihood or similar methods. We instead adopted a Bayesian modelling strategy, using weakly informative priors to aid convergence. This meant that some complex procedures anticipated in the SAP could not feasibly be carried out, including adapted lasso methods and fractional polynomials. We also chose to model ordinal outcomes as binary to aid interpretation of relatively complex results.

The text that follows explains the statistical methods used in more detail than the manuscript.

**Table: Deviations from the Statistical Analysis Plan (SAP)**

| **Section in the SAP** | **Deviation** | **Reason** |
| --- | --- | --- |
| Aim 2 | Only Cohort A was used for this analysis. | Analysis under Aim 1 revealed considerable between-country variation and resulted in all countries being used for analysis. This led to a shift in focus on estimating between-country variation and examining variables that may account for it. This analysis would have been biased if Cohort B had been included, since countries differed considerably in the number of patients they submitted for Cohort B. |
| Aim 2 | Lasso shrinkage was not used | The decision to model between-country variation necessitated a three-level model (patients within hospitals within countries) deployed for Aim 2, which was made more complicated by the presence of zeroes (hospitals and countries with no PVI use), and hence necessitated Bayesian estimation for model convergence, as well as multiple imputation of missing values. The model complexity was thus already high, and a decision was made not to add to it by additionally employing lasso shrinkage. |
| Aim 2 | Stratification by type of anaesthesia not done. | Planned at a later stage. |
| Aim 2 | Fractional polynomials not used. | Age was the only predictor modelled as a continuous variable. There is no standard routine for using fractional polynomials in combination with Bayesian models. Exploration suggested that a simple linear + square transformation modelled the relationship well. |
| Aim 2 | Changes in the list of predictors (Appendix A in the SAP) | Some intraoperative covariates were mistakenly listed under “pre-operative predictors”. Some covariates were added after consideration (e.g. intraoperative MAP). The preoperative covariates that were used can be seen in Table S5. The intraoperative covariates used can be seen in Table S6. |
| Aim 3 | Ordered outcomes were modelled as binary instead. | Since models were relatively complex analytically, and the analyses are essentially exploratory, a simpler approach was preferred. |
| Aim 4 | Information on vasopressor dosage was only collected in the UK and thus not analysed for this manuscript. | Half of national coordinators said this would be too onerous to collect. |
| Aim 6 | Adaptive lasso was not used. | The model complexity (Bayesian three-level models) was high, and a decision was made not to add to it by additionally employing lasso shrinkage. |

**Modelling PVI use**

Models of our primary outcome, PVI use, were estimated on Cohort A only. To model the variation of PVI use across hospitals and countries in the presence of sparsity (no observed PVI use in some hospitals and some countries), we used Bayesian mixed effects logistic regression with random intercepts for hospitals and countries. We used a weakly informative prior centred on our prior estimate of 2 % for the fixed intercept [Normal(-3.89, 100)] and non-informative priors [Cauchy(0, 10)] for the random effect standard deviations. These priors are so weak as to have essentially no effect on the point estimates or credible intervals, but they aid convergence. We call this Model 1. Estimates of the random effect variances were transformed into median odds ratios. To account for regression to the mean, country- and hospital-specific estimates of PVI use were derived using best linear unbiased estimators.

As outlined in the SAP, the distribution of country-level PVI use was visualized graphically (see Figure 1 in the manuscript, and Figures S1, S2, and S3 in the appendix) and discussed within the team, with respect to the decision whether to analyse data from high-income countries separately. There was some association of country income level and PVI use, whereby higher income in a country was associated with higher rates of PVI use. However, there was no clear-cut division of PVI use rates by country income. We thus decided to analyse data from all countries in all subsequent analyses.

To explore whether characteristics of the patient and surgical procedure could explain some of the between-country and between-hospital variation, we extended Model 1 by using a prespecified set of pre-operative variables as predictors, and estimating their fixed effects (using weakly informative default priors; Model 2). Finally, in Model 3, we added a set of prespecified intraoperative variables to Model 2. Models 2 and 3 also had the further purpose to explore associations of patient and procedure characteristics with PVI use. In both Models 2 and 3, we used the same priors for the intercept and random effect standard deviations as in Model 1. We used flat priors for the coefficients of all covariates.

Some covariates used in Models 2 & 3 had missing observations. The percentages of cases with at least one missing value were 27 % and 28 % for Models 2 and 3, respectively. We used multiple imputation for missing values to impute 30 data sets. The imputation model was the same for both Model 2 and Model 3, and contained all pre-operative variables, all intraoperative variables, and (as auxiliary variables) all outcomes. Models were estimated on all 30 data sets, with four Monte Carlo chains per data set. Point estimates of parameters were derived as the means of the posterior distribution, while the 2.5th and 97.5th percentiles were taken as the limits of 95 % credible intervals.

**Exploring patient outcomes**

To explore patient outcomes associated with PVI use, we combined data from cohorts A and B. We conducted a comparison of Cohort B with PVI recipients in Cohort A to gauge the extent of potential selection bias. This analysis is presented in supplementary table S1. In summary, compared to PVI recipients in Cohort A, Cohort B were more likely to come from the UK, to have urgent surgery, to have major surgery, to have an intraoperative MAP < 90 mmHg, and to have an adverse outcome (Ventilation, Renal replacement therapy, Acute kidney injury, Parenteral nutrition, Atrial fibrillation, Antibiotics, Mortality). Cohort B also had longer median length of stay than PVI recipients in Cohort A.

We modelled associations between PVI use and outcomes using Bayesian multilevel logistic regression, with random intercepts for centre and country and adjusting for pre-operative variables. Bayesian multilevel quantile regression was used to model length of stay. A binary indicator for Cohort (A or B) was added to these models to adjust for residual selection bias. We used flat priors for all covariates, including for PVI use.

To explore the relationship between exposure to vasopressors (both intra- and postoperatively), we grouped patients from both cohorts into five groups: no vasopressors, intraoperative vasopressors only, boluses and enteral vasopressors (but not PVI), short-term PVI (1-2 days post-operatively) and prolonged PVI (3 days or more). We descriptively compared percentages of adverse outcomes, as well as the distribution of length of stay, across these five groups.

# **Figures**

## **Figure S1: Observed percentage of PVI by number of patients per hospital.**


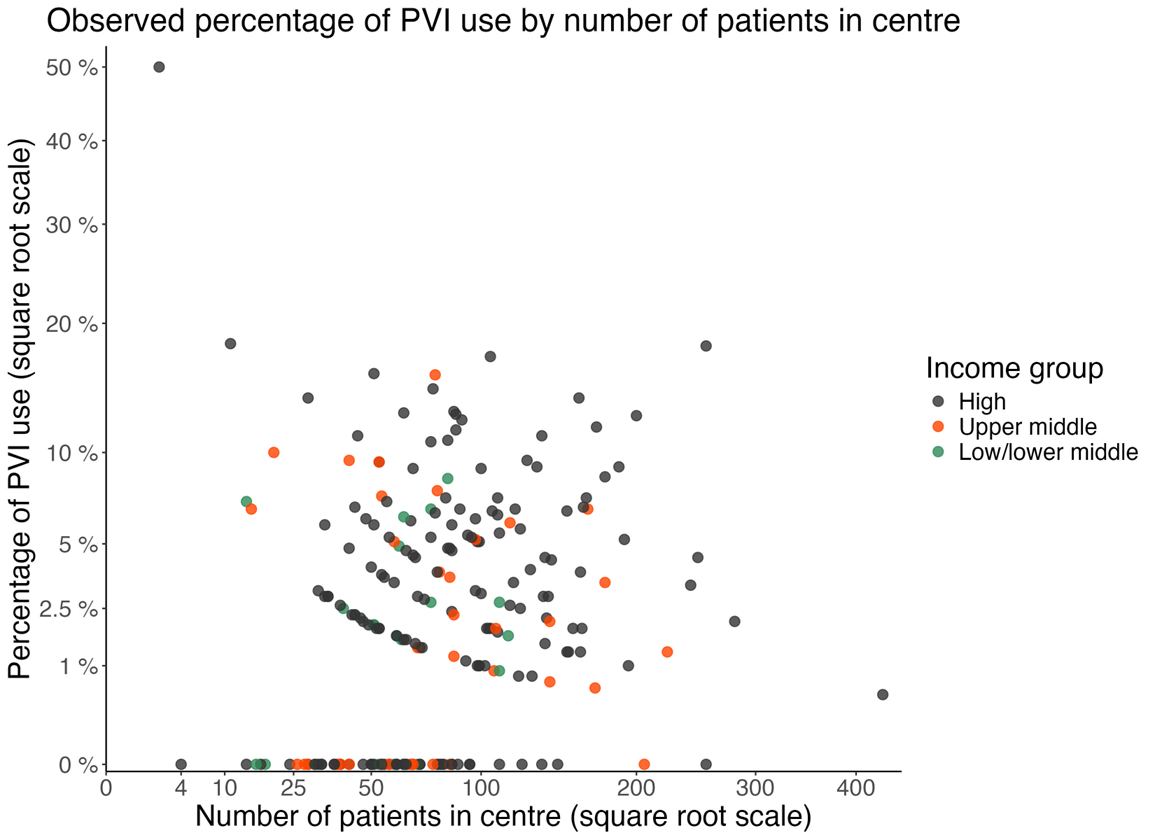


Observed percentage of Postoperative Vasopressor Infusion (PVI) by number of patients in hospital.

The scatter plot demonstrates the relationship between hospital size (x-axis) and the percentage of PVI use (y-axis), both variables presented on square root scales. Income groups defined by the 2023 World Bank Classification System.

The curvilinear patterns observed in the data distribution reflect the nature of the percentage calculations in smaller hospitals, where the possible values for PVI use are constrained by the denominator. For instance, in a center with 50 patients, PVI use can only occur in 2% increments (0%, 2%, 4%, etc.), as fractional patients are not possible.

## **Figure S2: Observed percentage of PVI use by number of patients in country**


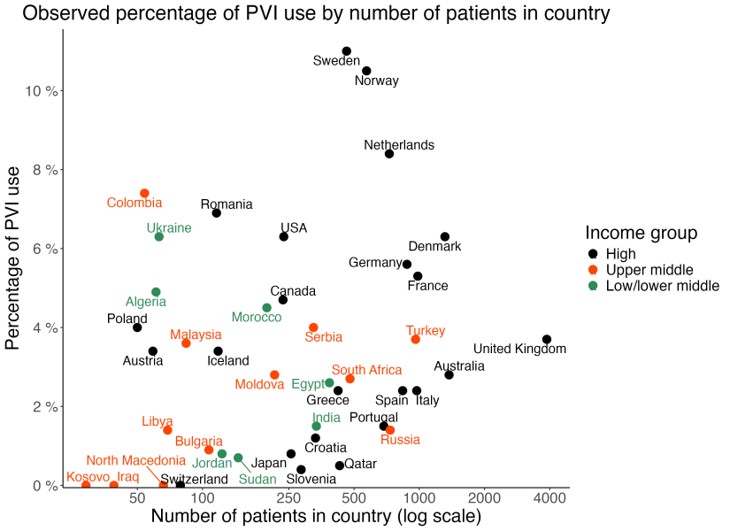


Observed percentage of PVI use by number of patients in country in Cohort A (n = 19,768). Income groups were defined using the 2023 World Bank Classification System.

## **Figure S3: Estimated percentage of PVI use in countries with ≥500 patients and ≥6 participating hospitals.**


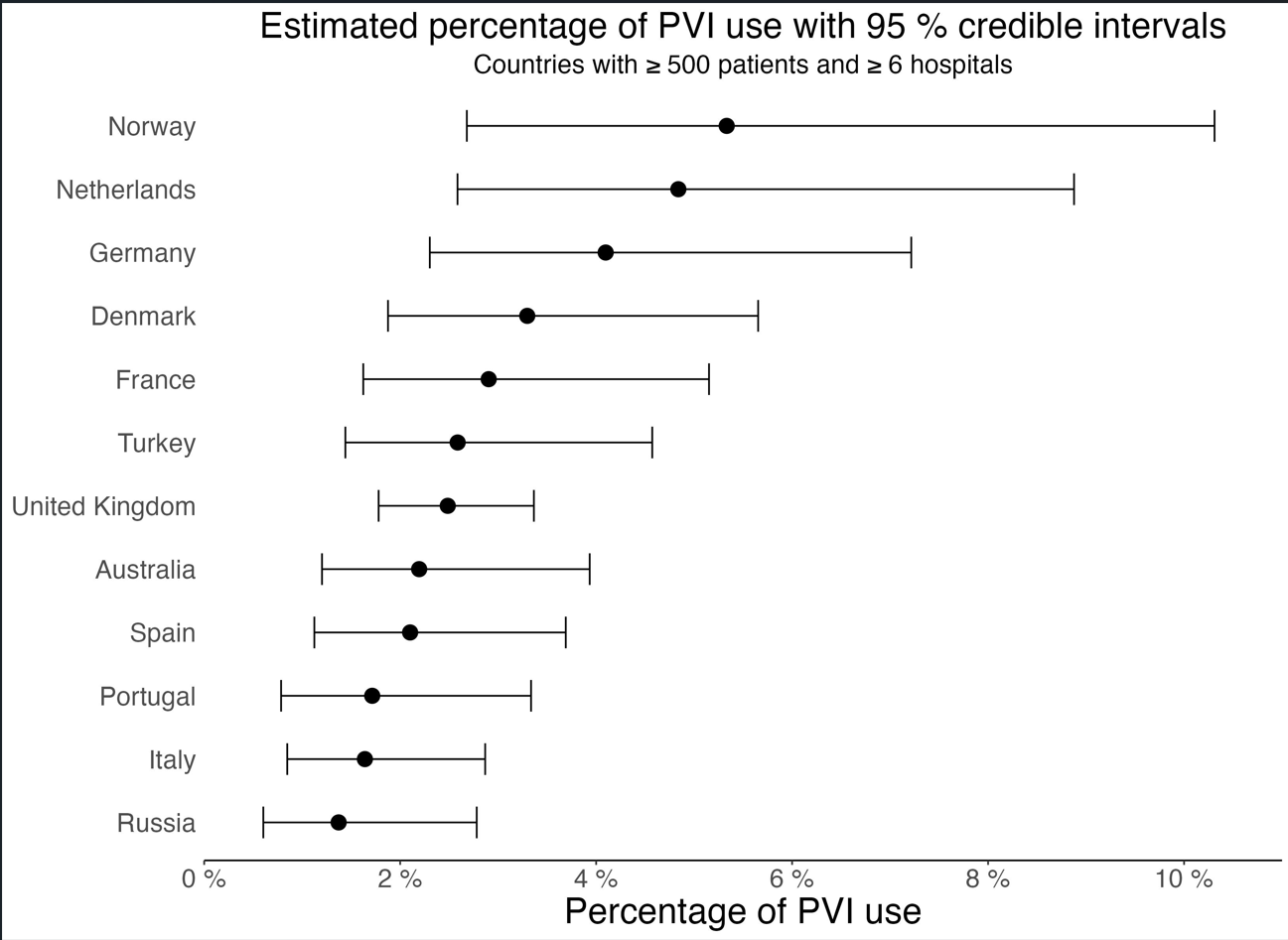


Estimated percentage of PVI use in Cohort A with 95% credible intervals (best linear unbiased estimates) from Bayesian multilevel logistic regression with random effects for country and centre, but without casemix variables. To discourage over-interpretation of estimates from countries with small sample sizes, we included in this graph only countries that contributed ≥500 patients and ≥6 hospitals.

# **Tables**

## **Table S1: Full Baseline description of the sample including data of missingness**

**Table S1a: Preoperative patient characteristics**

|  |  | **Cohort A: Total**  **(N=19,768)** | | **Coh A: No PVI**  **(N=18,998)** | | **Coh A: PVI**  **(N=770)** | | **Cohort B**  **(N=5,907)** | |
| --- | --- | --- | --- | --- | --- | --- | --- | --- | --- |
| **Characteristic** | **Category** | **Number** | **(%)** | **Number** | **(%)** | **Number** | **(%)** | **Number** | **(%)** |
| Age group | <50 | 6281 | (31.8) | 6175 | (32.5) | 106 | (13.8) | 861 | (14.6) |
|  | 50-69 | 7455 | (37.7) | 7131 | (37.5) | 324 | (42.1) | 2286 | (38.7) |
|  | >=70 | 6032 | (30.5) | 5692 | (30.0) | 340 | (44.2) | 2760 | (46.7) |
|  | Missing | 0 |  | 0 |  | 0 |  | 0 |  |
| Sex | Female | 9730 | (49.4) | 9428 | (49.8) | 302 | (39.3) | 2461 | (41.9) |
|  | Male | 9975 | (50.6) | 9508 | (50.2) | 467 | (60.7) | 3406 | (58.1) |
|  | Missing | 63 |  | 62 |  | 1 |  | 40 |  |
| ASA Grade | ASA 1 | 3720 | (18.9) | 3692 | (19.5) | 28 | ( 3.6) | 262 | ( 4.4) |
|  | ASA 2 | 9064 | (45.9) | 8852 | (46.7) | 212 | (27.6) | 1424 | (24.1) |
|  | ASA 3 | 5994 | (30.4) | 5632 | (29.7) | 362 | (47.1) | 2632 | (44.6) |
|  | ASA 4 | 900 | ( 4.6) | 757 | ( 4.0) | 143 | (18.6) | 1393 | (23.6) |
|  | ASA 5 | 53 | ( 0.3) | 30 | ( 0.2) | 23 | ( 3.0) | 190 | ( 3.2) |
|  | Missing | 37 |  | 35 |  | 2 |  | 6 |  |
| Clinical Frailty Scale | 1 | 2495 | (13.3) | 2451 | (13.6) | 44 | ( 6.0) | 215 | ( 3.8) |
|  | 2 | 6191 | (32.9) | 6028 | (33.4) | 163 | (22.1) | 1136 | (20.1) |
|  | 3 | 5367 | (28.6) | 5156 | (28.5) | 211 | (28.7) | 1739 | (30.8) |
|  | 4 | 2409 | (12.8) | 2270 | (12.6) | 139 | (18.9) | 1164 | (20.6) |
|  | 5 | 937 | ( 5.0) | 873 | ( 4.8) | 64 | ( 8.7) | 499 | ( 8.8) |
|  | 6 | 731 | ( 3.9) | 691 | ( 3.8) | 40 | ( 5.4) | 421 | ( 7.5) |
|  | 7 | 505 | ( 2.7) | 461 | ( 2.6) | 44 | ( 6.0) | 336 | ( 6.0) |
|  | 8 | 134 | ( 0.7) | 109 | ( 0.6) | 25 | ( 3.4) | 102 | ( 1.8) |
|  | 9 | 27 | ( 0.1) | 21 | ( 0.1) | 6 | ( 0.8) | 33 | ( 0.6) |
|  | Missing | 972 |  | 938 |  | 34 |  | 262 |  |
| Mean arterial pressure | <90 mmHg | 4854 | (30.5) | 4614 | (30.1) | 240 | (39.2) | 1759 | (40.3) |
| (12 hours before surgery) | 90-95.99 mgHg | 3203 | (20.1) | 3093 | (20.2) | 110 | (18.0) | 801 | (18.4) |
|  | >=96 mmHg | 7866 | (49.4) | 7604 | (49.7) | 262 | (42.8) | 1805 | (41.4) |
|  | Missing | 3845 |  | 3687 |  | 158 |  | 1542 |  |
| Mean arterial pressure | <90 mmHg | 5001 | (27.7) | 4688 | (27.0) | 313 | (44.1) | 2554 | (49.9) |
| (immediately prior to surgery) | 90-95.99 mgHg | 2935 | (16.2) | 2849 | (16.4) | 86 | (12.1) | 652 | (12.7) |
|  | >=96 mmHg | 10130 | (56.1) | 9820 | (56.6) | 310 | (43.7) | 1909 | (37.3) |
|  | Missing | 1702 |  | 1641 |  | 61 |  | 792 |  |

**Table S1b: Medical history**

|  |  | **Cohort A: Total**  **(N=19,768)** | | **Coh A: No PVI**  **(N=18,998)** | | **Coh A: PVI**  **(N=770)** | | **Cohort B**  **(N=5,907)** | |
| --- | --- | --- | --- | --- | --- | --- | --- | --- | --- |
| **Medical history** | **Category** | **Number** | **(%)** | **Number** | **(%)** | **Number** | **(%)** | **Number** | **(%)** |
| Coronary artery disease | No | 17890 | (90.5) | 17271 | (90.9) | 619 | (80.4) | 4866 | (82.4) |
|  | Yes | 1878 | ( 9.5) | 1727 | ( 9.1) | 151 | (19.6) | 1037 | (17.6) |
|  | Missing | 0 |  | 0 |  | 0 |  | 4 |  |
| Cerebrovascular disease | No | 18433 | (93.2) | 17773 | (93.6) | 660 | (85.7) | 5312 | (90.0) |
|  | Yes | 1335 | ( 6.8) | 1225 | ( 6.4) | 110 | (14.3) | 592 | (10.0) |
|  | Missing | 0 |  | 0 |  | 0 |  | 3 |  |
| Peripheral vascular disease | No | 18443 | (93.3) | 17775 | (93.6) | 668 | (86.8) | 5166 | (87.5) |
|  | Yes | 1325 | ( 6.7) | 1223 | ( 6.4) | 102 | (13.2) | 738 | (12.5) |
|  | Missing | 0 |  | 0 |  | 0 |  | 3 |  |
| Arterial fibrillation | No | 18381 | (93.0) | 17728 | (93.3) | 653 | (84.8) | 5037 | (85.3) |
|  | Yes | 1387 | ( 7.0) | 1270 | ( 6.7) | 117 | (15.2) | 867 | (14.7) |
|  | Missing | 0 |  | 0 |  | 0 |  | 3 |  |
| Heart failure | No | 18698 | (94.6) | 18011 | (94.8) | 687 | (89.2) | 5196 | (88.0) |
|  | Yes | 1069 | ( 5.4) | 986 | ( 5.2) | 83 | (10.8) | 707 | (12.0) |
|  | Missing | 1 |  | 1 |  | 0 |  | 4 |  |
| Hypertension | No | 11886 | (60.1) | 11529 | (60.7) | 357 | (46.4) | 2806 | (47.5) |
|  | Yes | 7881 | (39.9) | 7468 | (39.3) | 413 | (53.6) | 3097 | (52.5) |
|  | Missing | 1 |  | 1 |  | 0 |  | 4 |  |
| Diabetes | No | 16624 | (84.1) | 16028 | (84.4) | 596 | (77.4) | 4492 | (76.1) |
|  | Insulin-dependent | 990 | ( 5.0) | 934 | ( 4.9) | 56 | ( 7.3) | 503 | ( 8.5) |
|  | Non-insulin dependent | 2152 | (10.9) | 2034 | (10.7) | 118 | (15.3) | 909 | (15.4) |
|  | Missing | 2 |  | 2 |  | 0 |  | 3 |  |
| Chronic liver disease | No | 19247 | (97.4) | 18522 | (97.5) | 725 | (94.2) | 5577 | (94.5) |
|  | Yes | 520 | ( 2.6) | 475 | ( 2.5) | 45 | ( 5.8) | 325 | ( 5.5) |
|  | Missing | 1 |  | 1 |  | 0 |  | 5 |  |
| Chronic respiratory disease | No | 17153 | (86.8) | 16533 | (87.0) | 620 | (80.5) | 4726 | (80.1) |
|  | COPD | 1243 | ( 6.3) | 1152 | ( 6.1) | 91 | (11.8) | 745 | (12.6) |
|  | Other | 1371 | ( 6.9) | 1312 | ( 6.9) | 59 | ( 7.7) | 432 | ( 7.3) |
|  | Missing | 1 |  | 1 |  | 0 |  | 4 |  |
| Steroid use | No | 19073 | (96.5) | 18338 | (96.5) | 735 | (95.5) | 5628 | (95.4) |
|  | Yes | 692 | ( 3.5) | 657 | ( 3.5) | 35 | ( 4.5) | 273 | ( 4.6) |
|  | Missing | 3 |  | 3 |  | 0 |  | 6 |  |

**Table S1c: Regular mediations**

|  |  | **Cohort A: Total**  **(N=19,768)** | | **Coh A: No PVI**  **(N=18,998)** | | **Coh A: PVI**  **(N=770)** | | **Cohort B**  **(N=5,907)** | |
| --- | --- | --- | --- | --- | --- | --- | --- | --- | --- |
| **Medication** | **Category** | **Number** | **(%)** | **Number** | **(%)** | **Number** | **(%)** | **Number** | **(%)** |
| Alpha Blocker | no | 19250 | (97.4) | 18505 | (97.4) | 745 | (96.8) | 5731 | (97.0) |
|  | yes: took day of surgery | 189 | ( 1.0) | 180 | ( 0.9) | 9 | ( 1.2) | 51 | ( 0.9) |
|  | yes: omitted day of surgery | 212 | ( 1.1) | 198 | ( 1.0) | 14 | ( 1.8) | 69 | ( 1.2) |
|  | yes: unknown | 117 | ( 0.6) | 115 | ( 0.6) | 2 | ( 0.3) | 56 | ( 0.9) |
| Angio-receptor blocker | no | 17691 | (89.5) | 17027 | (89.6) | 664 | (86.2) | 5140 | (87.0) |
|  | yes: took day of surgery | 689 | ( 3.5) | 663 | ( 3.5) | 26 | ( 3.4) | 187 | ( 3.2) |
|  | yes: omitted day of surgery | 1091 | ( 5.5) | 1025 | ( 5.4) | 66 | ( 8.6) | 396 | ( 6.7) |
|  | yes: unknown | 297 | ( 1.5) | 283 | ( 1.5) | 14 | ( 1.8) | 184 | ( 3.1) |
| Beta blocker | no | 16667 | (84.3) | 16135 | (84.9) | 532 | (69.1) | 4282 | (72.5) |
|  | yes: took day of surgery | 2020 | (10.2) | 1890 | ( 9.9) | 130 | (16.9) | 851 | (14.4) |
|  | yes: omitted day of surgery | 645 | ( 3.3) | 581 | ( 3.1) | 64 | ( 8.3) | 377 | ( 6.4) |
|  | yes: unknown | 436 | ( 2.2) | 392 | ( 2.1) | 44 | ( 5.7) | 397 | ( 6.7) |
| Calcium blocker | no | 17319 | (87.6) | 16681 | (87.8) | 638 | (82.9) | 4966 | (84.1) |
|  | yes: took day of surgery | 1160 | ( 5.9) | 1113 | ( 5.9) | 47 | ( 6.1) | 335 | ( 5.7) |
|  | yes: omitted day of surgery | 842 | ( 4.3) | 779 | ( 4.1) | 63 | ( 8.2) | 309 | ( 5.2) |
|  | yes: unknown | 447 | ( 2.3) | 425 | ( 2.2) | 22 | ( 2.9) | 297 | ( 5.0) |
| Diuretic | no | 17604 | (89.1) | 16980 | (89.4) | 624 | (81.0) | 4912 | (83.2) |
|  | yes: took day of surgery | 779 | ( 3.9) | 740 | ( 3.9) | 39 | ( 5.1) | 294 | ( 5.0) |
|  | yes: omitted day of surgery | 1024 | ( 5.2) | 949 | ( 5.0) | 75 | ( 9.7) | 421 | ( 7.1) |
|  | yes: unknown | 361 | ( 1.8) | 329 | ( 1.7) | 32 | ( 4.2) | 280 | ( 4.7) |
| Any antihypertensive | No | 12215 | (61.8) | 11867 | (62.5) | 348 | (45.2) | 2780 | (47.1) |
|  | Yes | 7553 | (38.2) | 7131 | (37.5) | 422 | (54.8) | 3127 | (52.9) |
| NSAID | no | 19000 | (96.1) | 18256 | (96.1) | 744 | (96.6) | 5712 | (96.7) |
|  | yes: took day of surgery | 233 | ( 1.2) | 226 | ( 1.2) | 7 | ( 0.9) | 48 | ( 0.8) |
|  | yes: omitted day of surgery | 372 | ( 1.9) | 361 | ( 1.9) | 11 | ( 1.4) | 69 | ( 1.2) |
|  | yes: unknown | 163 | ( 0.8) | 155 | ( 0.8) | 8 | ( 1.0) | 78 | ( 1.3) |

*Note: no missing values recorded for regular medications.*

**Table S1d: Procedure characteristics**

|  |  | **Cohort A: Total**  **(N=19,768)** | | **Coh A: No PVI**  **(N=18,998)** | | **Coh A: PVI**  **(N=770)** | | **Cohort B**  **(N=5,907)** | |
| --- | --- | --- | --- | --- | --- | --- | --- | --- | --- |
| **Characteristic** | **Category** | **Number** | **(%)** | **Number** | **(%)** | **Number** | **(%)** | **Number** | **(%)** |
| Reason for surgery | Infection | 2841 | (14.4) | 2711 | (14.3) | 130 | (16.9) | 1218 | (20.6) |
|  | Fracture | 4739 | (24.0) | 4463 | (23.5) | 276 | (35.8) | 1866 | (31.6) |
|  | Cancer | 2862 | (14.5) | 2794 | (14.7) | 68 | ( 8.8) | 433 | ( 7.3) |
|  | Bleeding | 760 | ( 3.8) | 689 | ( 3.6) | 71 | ( 9.2) | 604 | (10.2) |
|  | Other | 8566 | (43.3) | 8341 | (43.9) | 225 | (29.2) | 1785 | (30.2) |
|  | Missing | 0 |  | 0 |  | 0 |  | 1 |  |
| Surgical procedure | Breast | 598 | ( 3.0) | 594 | ( 3.1) | 4 | ( 0.5) | 21 | ( 0.4) |
|  | Gynaecological | 1383 | ( 7.0) | 1354 | ( 7.1) | 29 | ( 3.8) | 196 | ( 3.3) |
|  | Head and neck | 1920 | ( 9.7) | 1870 | ( 9.8) | 50 | ( 6.5) | 246 | ( 4.2) |
|  | Hepato-biliary | 979 | ( 5.0) | 920 | ( 4.8) | 59 | ( 7.7) | 457 | ( 7.7) |
|  | Kidney/urological | 2194 | (11.1) | 2129 | (11.2) | 65 | ( 8.4) | 436 | ( 7.4) |
|  | Lower gastro-intestinal | 2781 | (14.1) | 2610 | (13.7) | 171 | (22.2) | 1853 | (31.4) |
|  | Orthopaedic | 4953 | (25.1) | 4853 | (25.5) | 100 | (13.0) | 711 | (12.0) |
|  | Plastics / Cutaneous | 902 | ( 4.6) | 886 | ( 4.7) | 16 | ( 2.1) | 120 | ( 2.0) |
|  | Upper gastro-intestinal | 1271 | ( 6.4) | 1172 | ( 6.2) | 99 | (12.9) | 729 | (12.3) |
|  | Neurological/spinal | 1154 | ( 5.8) | 1093 | ( 5.8) | 61 | ( 7.9) | 281 | ( 4.8) |
|  | Vascular | 920 | ( 4.7) | 830 | ( 4.4) | 90 | (11.7) | 592 | (10.0) |
|  | Other | 711 | ( 3.6) | 685 | ( 3.6) | 26 | ( 3.4) | 264 | ( 4.5) |
|  | Missing | 2 |  | 2 |  | 0 |  | 1 |  |
| Severity | Minor | 2553 | (12.9) | 2536 | (13.4) | 17 | ( 2.2) | 102 | ( 1.7) |
|  | Intermediate | 9857 | (49.9) | 9678 | (51.0) | 179 | (23.2) | 938 | (15.9) |
|  | Major | 7354 | (37.2) | 6780 | (35.7) | 574 | (74.5) | 4866 | (82.4) |
|  | Missing | 4 |  | 4 |  | 0 |  | 1 |  |
| Urgency | Urgent | 6449 | (32.6) | 6069 | (32.0) | 380 | (49.4) | 3524 | (59.7) |
|  | Not urgent | 13306 | (67.4) | 12917 | (68.0) | 389 | (50.6) | 2380 | (40.3) |
|  | Missing | 13 |  | 12 |  | 1 |  | 3 |  |

**Table S1e: Intraoperative variables**

|  |  | **Cohort A: Total**  **(N=19,768)** | | **Coh A: No PVI**  **(N=18,998)** | | **Coh A: PVI**  **(N=770)** | | **Cohort B**  **(N=5,907)** | |
| --- | --- | --- | --- | --- | --- | --- | --- | --- | --- |
| **Variable** | **Category** | **Number** | **(%)** | **Number** | **(%)** | **Number** | **(%)** | **Number** | **(%)** |
| Airway | Endotracheal tube | 12980 | (65.9) | 12273 | (64.8) | 707 | (91.9) | 5415 | (91.9) |
|  | Supraglottic | 2566 | (13.0) | 2553 | (13.5) | 13 | ( 1.7) | 94 | ( 1.6) |
|  | O2 facemask or nasal canula | 4156 | (21.1) | 4107 | (21.7) | 49 | ( 6.4) | 386 | ( 6.5) |
|  | Missing | 66 |  | 65 |  | 1 |  | 12 |  |
| Blood loss | <250 ml | 15794 | (80.6) | 15434 | (81.9) | 360 | (47.4) | 2749 | (47.2) |
|  | 251-1000 ml | 3359 | (17.1) | 3092 | (16.4) | 267 | (35.2) | 2024 | (34.7) |
|  | 1001-3000 ml | 402 | ( 2.1) | 294 | ( 1.6) | 108 | (14.2) | 828 | (14.2) |
|  | >3000 ml | 43 | ( 0.2) | 19 | ( 0.1) | 24 | ( 3.2) | 224 | ( 3.8) |
|  | Missing | 170 |  | 159 |  | 11 |  | 82 |  |
| Duration of operation | <120 mins | 6955 | (35.3) | 6869 | (36.3) | 86 | (11.2) | 628 | (10.7) |
|  | 120 - 239 mins | 7985 | (40.6) | 7786 | (41.1) | 199 | (26.0) | 1706 | (29.1) |
|  | >=240 mins | 4748 | (24.1) | 4268 | (22.6) | 480 | (62.7) | 3522 | (60.1) |
|  | Missing | 80 |  | 75 |  | 5 |  | 51 |  |
| Mean arterial pressure | <90 mmHg | 18286 | (93.2) | 17540 | (93.0) | 746 | (98.2) | 5807 | (98.9) |
| (intra-operative**)** | 90-95.99 mgHg | 700 | ( 3.6) | 693 | ( 3.7) | 7 | ( 0.9) | 36 | ( 0.6) |
|  | >=96 mmHg | 640 | ( 3.3) | 633 | ( 3.4) | 7 | ( 0.9) | 31 | ( 0.5) |
|  | Missing | 142 |  | 132 |  | 10 |  | 33 |  |

**Table S1f: Type of anaesthesia**

|  |  | **Cohort A: Total**  **(N=19,768)** | | **Coh A: No PVI**  **(N=18,998)** | | **Coh A: PVI**  **(N=770)** | | **Cohort B**  **(N=5,907)** | |
| --- | --- | --- | --- | --- | --- | --- | --- | --- | --- |
| **Type of anaesthesia** | **Category** | **Number** | **(%)** | **Number** | **(%)** | **Number** | **(%)** | **Number** | **(%)** |
| Volatile | No | 9188 | (46.5) | 8901 | (46.9) | 287 | (37.3) | 1924 | (32.6) |
|  | Yes | 10564 | (53.5) | 10082 | (53.1) | 482 | (62.7) | 3981 | (67.4) |
|  | Missing | 16 |  | 15 |  | 1 |  | 2 |  |
| TIVA | No | 14333 | (72.6) | 13810 | (72.7) | 523 | (68.0) | 4336 | (73.4) |
|  | Yes | 5419 | (27.4) | 5173 | (27.3) | 246 | (32.0) | 1569 | (26.6) |
|  | Missing | 16 |  | 15 |  | 1 |  | 2 |  |
| Sedation | No | 18303 | (92.7) | 17557 | (92.5) | 746 | (97.0) | 5775 | (97.8) |
|  | Yes | 1449 | ( 7.3) | 1426 | ( 7.5) | 23 | ( 3.0) | 130 | ( 2.2) |
|  | Missing | 16 |  | 15 |  | 1 |  | 2 |  |
| Regional | No | 17987 | (91.1) | 17264 | (90.9) | 723 | (94.0) | 5690 | (96.4) |
|  | Yes | 1765 | ( 8.9) | 1719 | ( 9.1) | 46 | ( 6.0) | 215 | ( 3.6) |
|  | Missing | 16 |  | 15 |  | 1 |  | 2 |  |
| Spinal | No | 16543 | (83.8) | 15823 | (83.4) | 720 | (93.6) | 5391 | (91.3) |
|  | Yes | 3209 | (16.2) | 3160 | (16.6) | 49 | ( 6.4) | 514 | ( 8.7) |
|  | Missing | 16 |  | 15 |  | 1 |  | 2 |  |
| Epidural | No | 18934 | (95.9) | 18323 | (96.5) | 611 | (79.5) | 4933 | (83.5) |
|  | Yes | 818 | ( 4.1) | 660 | ( 3.5) | 158 | (20.5) | 972 | (16.5) |
|  | Missing | 16 |  | 15 |  | 1 |  | 2 |  |

**Table S1g: Peri-operative vasopressors**

|  |  | **Cohort A: Total**  **(N=19,768)** | | **Coh A: No PVI**  **(N=18,998)** | | **Coh A: PVI**  **(N=770)** | | **Cohort B**  **(N=5,907)** | |
| --- | --- | --- | --- | --- | --- | --- | --- | --- | --- |
| **Vasopressors** | **Category** | **Number** | **(%)** | **Number** | **(%)** | **Number** | **(%)** | **Number** | **(%)** |
| Preoperative vasopressors | No | 19517 | (98.7) | 18845 | (99.2) | 672 | (87.3) | 5255 | (89.0) |
|  | Yes | 248 | ( 1.3) | 150 | ( 0.8) | 98 | (12.7) | 647 | (11.0) |
|  | Missing | 3 |  | 3 |  | 0 |  | 5 |  |
| Any intraoperative vasopressor | 0 | 13295 | (67.3) | 13214 | (69.6) | 81 | (10.5) | 574 | ( 9.7) |
|  | 1 | 6473 | (32.7) | 5784 | (30.4) | 689 | (89.5) | 5333 | (90.3) |
|  | Missing | 0 |  | 0 |  | 0 |  | 0 |  |
| Enteral vasopressors | No | 19684 | (99.6) | 18947 | (99.7) | 737 | (95.7) | 5578 | (94.4) |
|  | Yes | 84 | ( 0.4) | 51 | ( 0.3) | 33 | ( 4.3) | 329 | ( 5.6) |
|  | Missing | 0 |  | 0 |  | 0 |  | 0 |  |
| Vasopressor bolus | No | 19103 | (96.6) | 18469 | (97.2) | 634 | (82.3) | 4629 | (78.4) |
|  | Yes | 665 | ( 3.4) | 529 | ( 2.8) | 136 | (17.7) | 1278 | (21.6) |
|  | Missing | 0 |  | 0 |  | 0 |  | 0 |  |

**Table S1h: Outcomes**

|  |  | **Cohort A: Total**  **(N=19,768)** | | **Coh A: No PVI**  **(N=18,998)** | | **Coh A: PVI**  **(N=770)** | | **Cohort B**  **(N=5,907)** | |
| --- | --- | --- | --- | --- | --- | --- | --- | --- | --- |
| **Outcome** | **Category** | **Number** | **(%)** | **Number** | **(%)** | **Number** | **(%)** | **Number** | **(%)** |
| Ventilation | No | 18871 | (95.8) | 18396 | (97.2) | 475 | (61.7) | 2872 | (48.6) |
|  | Yes | 822 | ( 4.2) | 527 | ( 2.8) | 295 | (38.3) | 3032 | (51.4) |
|  | Missing | 75 |  | 75 |  | 0 |  | 3 |  |
| Myocardial infarction | No | 19629 | (99.7) | 18872 | (99.7) | 757 | (98.3) | 5738 | (97.2) |
|  | Yes | 63 | ( 0.3) | 50 | ( 0.3) | 13 | ( 1.7) | 165 | ( 2.8) |
|  | Missing | 76 |  | 76 |  | 0 |  | 4 |  |
| Atrial fibrillation | No | 19535 | (99.2) | 18802 | (99.4) | 733 | (95.2) | 5489 | (93.0) |
|  | Yes | 156 | ( 0.8) | 119 | ( 0.6) | 37 | ( 4.8) | 414 | ( 7.0) |
|  | Missing | 77 |  | 77 |  | 0 |  | 4 |  |
| Other dysrhythmia | No | 19483 | (98.9) | 18766 | (99.2) | 717 | (93.1) | 5523 | (93.6) |
|  | Yes | 207 | ( 1.1) | 154 | ( 0.8) | 53 | ( 6.9) | 380 | ( 6.4) |
|  | Missing | 78 |  | 78 |  | 0 |  | 4 |  |
| RRT | No | 19484 | (98.9) | 18759 | (99.1) | 725 | (94.2) | 5360 | (90.8) |
|  | Yes | 208 | ( 1.1) | 163 | ( 0.9) | 45 | ( 5.8) | 546 | ( 9.2) |
|  | Missing | 76 |  | 76 |  | 0 |  | 1 |  |
| Parenteral nutrition | No | 19072 | (96.9) | 18476 | (97.6) | 596 | (77.4) | 4351 | (73.7) |
|  | Yes | 619 | ( 3.1) | 445 | ( 2.4) | 174 | (22.6) | 1554 | (26.3) |
|  | Missing | 77 |  | 77 |  | 0 |  | 2 |  |
| Antibiotics | No | 17357 | (88.5) | 16891 | (89.6) | 466 | (61.6) | 3206 | (54.8) |
|  | Yes | 2245 | (11.5) | 1954 | (10.4) | 291 | (38.4) | 2649 | (45.2) |
|  | Missing | 166 |  | 153 |  | 13 |  | 52 |  |
| Any complications | No | 14439 | (73.4) | 14219 | (75.2) | 220 | (28.6) | 1572 | (26.7) |
|  | Yes | 5245 | (26.6) | 4695 | (24.8) | 550 | (71.4) | 4320 | (73.3) |
|  | Missing | 84 |  | 84 |  | 0 |  | 15 |  |
| Acute Kidney Injury | No | 7722 | (88.7) | 7290 | (89.9) | 432 | (71.4) | 3255 | (66.4) |
|  | Yes | 988 | (11.3) | 815 | (10.1) | 173 | (28.6) | 1650 | (33.6) |
|  | Missing | 11058 |  | 10893 |  | 165 |  | 1002 |  |
| 30-day mortality | No | 19250 | (97.9) | 18580 | (98.3) | 670 | (87.5) | 4932 | (84.1) |
|  | Yes | 410 | ( 2.1) | 314 | ( 1.7) | 96 | (12.5) | 929 | (15.9) |
|  | Missing | 108 |  | 104 |  | 4 |  | 46 |  |
| Length of Stay (days) | Median (IQR) | 3 | (1, 6) | 3 | (1, 6) | 10 | (6, 20) | 12 | (7, 23) |
|  | Missing | 617 |  | 496 |  | 121 |  | 1133 |  |

## **Table S2: Hospital characteristics**

Data describing recruiting centres showing the variety in size and distribution of specialties. N=214 out of 228 hospitals (6% have missing data). “number of hospitals beds” has n=211, all other n=214.

| **Variable** | **Median** | **(Min, P25, P75, Max)** |
| --- | --- | --- |
| Number of operating rooms | 12 | (2, 8, 19, 60) |
| Number of critical care beds | 21 | (0, 12, 40, 270) |
| **Number of hospital beds** | **Number** | **(%)** |
| <250 | 41 | (19) |
| >1000 | 30 | (14) |
| 250-500 | 65 | (31) |
| 501-750 | 45 | (21) |
| 751-1000 | 30 | (14) |
| **Specialty** | **Number** | **(%)** |
| Neurosurgery | 116 | (54) |
| ENT + Head & Neck | 172 | (80) |
| Thoracic | 110 | (51) |
| Transplant | 64 | (30) |
| Gastro_abdominal | 202 | (94) |
| Hepato_biliary_Pancreas | 157 | (73) |
| Urological_Kidney | 181 | (85) |
| Gynaecological | 173 | (81) |
| Orthopaedic | 188 | (88) |
| Trauma | 161 | (75) |
| Vascular | 140 | (65) |

## **Table S3: Overview of Squeeze data**

Overview of Squeeze data in Cohort A and Cohort B.

|  | **Cohort A** | **Cohort B** | **Combined data** |
| --- | --- | --- | --- |
| **Number of hospitals** | 228 | 199 | 228 |
| **Number of countries** | 42 | 40 | 42 |
| **Number of patients** | 19,768 | 5,907 | 25,675 |
| **Number of patients receiving PVI** | 770 | 5,907 | 6,677 |
| **Percentage of patients receiving PVI** | 3.9 % | 100 % |  |

## **Table S4: Estimated median odds ratios in variation of PVI use**

Estimated median odds ratios (MOR) with 95 % credible intervals adjusted for pre- and for pre- and postoperative predictor variables. The higher the MOR, the higher the variation. Interpretation of MORs is as follows:

Centre: The MOR comparing two patients with the same characteristics in different centres within the same country.

Country: The MOR comparing two patients with the same characteristics in different countries, each patient being treated at a centre with typical levels of PVI use in their respective country.

| **Model** | **No covariates** | **Adjusting for pre-operative predictor variables only** | **Adjusting for pre- and intra-operative predictors** |
| --- | --- | --- | --- |
| **Hospital** | 2.30 (1.96, 2.73) | 2.47 (2.10, 2.98) | 2.30 (1.91, 2.85) |
| **Country** | 1.78 (1.37, 2.44) | 2.02 (1.55, 2.79) | 1.95 (1.43, 2.84) |

## **Table S5: Pre-operative predictors of PVI: full model results**

Estimated odds ratios and random intercept standard deviations from a Bayesian mixed effects logistic regression of postoperative vasopressor infusion use in 19,768 patients (Squeeze Cohort A): Pre-operative predictors only. N = 19768, number of centres: 228; number of countries: 42 Number of events (PVI use): 770 (3.9 %). Missing values were imputed using multiple imputation with chained equations (mice) with 30 imputations. Bayesian posterior draws were combined to obtain a pooled posterior distribution. The estimates shown are the mean, 2.5th percentile and 97.5th percentile of the posterior distribution.

|  | Odds Ratio | 95 % credible interval | |
| --- | --- | --- | --- |
| Intercept (baseline odds) | 0.00011 | 0.00004 | 0.00027 |
| Age (centred, in years) | 1.00870 | 1.00161 | 1.01587 |
| Age(centred)^2^ | 0.99951 | 0.99921 | 0.99980 |
| Frailty (ref: CFS score 1) |  |  |  |
| CFS 2 | 1.04 | 0.71 | 1.53 |
| CFS 3 | 0.87 | 0.59 | 1.31 |
| CFS 4 | 0.92 | 0.60 | 1.43 |
| CFS 5 | 0.88 | 0.53 | 1.44 |
| CFS 6 | 0.76 | 0.44 | 1.32 |
| CFS 7 | 0.95 | 0.55 | 1.65 |
| CFS 8 | 2.15 | 1.03 | 4.40 |
| CFS 9 | 0.90 | 0.25 | 3.00 |
| MAP 12 hrs pre-surgery (ref: ≥ 96mgHG) |  |  |  |
| <90mmHg | 1.33 | 1.07 | 1.65 |
| 90-95.99mgHg | 1.12 | 0.87 | 1.46 |
| MAP immediately pre-surgery (ref: ≥ 96mgHG) |  |  |  |
| <90mmHg | 2.35 | 1.91 | 2.89 |
| 90-95.99mgHg | 1.07 | 0.81 | 1.41 |
| Reason for Surgery (ref: Other) |  |  |  |
| Infection | 1.67 | 1.25 | 2.22 |
| Fracture | 1.62 | 1.27 | 2.06 |
| Cancer | 1.01 | 0.66 | 1.54 |
| Bleeding | 1.73 | 1.19 | 2.51 |
| Surgical Procedure (ref: Orthopaedic) |  |  |  |
| Breast | 0.39 | 0.11 | 1.10 |
| Gynaecological | 1.77 | 1.03 | 3.00 |
| Head and neck | 2.19 | 1.37 | 3.47 |
| Hepato-biliary | 3.31 | 2.06 | 5.30 |
| Kidney/urological | 1.84 | 1.18 | 2.88 |
| Lower gastro-intestinal | 2.76 | 1.89 | 4.07 |
| Plastics/Cutaneous | 1.60 | 0.82 | 3.03 |
| Upper gastro-intestinal | 4.30 | 2.83 | 6.58 |
| Neurological/spinal | 1.57 | 1.00 | 2.46 |
| Vascular | 3.42 | 2.17 | 5.41 |
| Other | 2.03 | 1.14 | 3.54 |
| Severity (ref: Minor) |  |  |  |
| Intermediate | 4.59 | 2.75 | 8.05 |
| Major | 24.06 | 14.35 | 42.33 |
| ASA Grade (ref: ASA 1) |  |  |  |
| ASA2 | 2.72 | 1.76 | 4.30 |
| ASA3 | 4.93 | 3.11 | 7.99 |
| ASA4 | 14.29 | 8.47 | 24.53 |
| ASA5 | 42.41 | 17.71 | 102.18 |
| Urgency (ref: Not urgent) |  |  |  |
| Urgent | 1.82 | 1.46 | 2.27 |
| Medical history |  |  |  |
| Arterial fibrillation | 1.21 | 0.92 | 1.59 |
| Cerebrovascular | 1.28 | 0.98 | 1.66 |
| Coronary Artery | 1.20 | 0.93 | 1.53 |
| Diabetes: insulin dependent | 0.82 | 0.58 | 1.15 |
| Diabetes: non-insulin dependent | 1.05 | 0.82 | 1.35 |
| Heart failure | 0.89 | 0.64 | 1.22 |
| Hypertension | 1.06 | 0.84 | 1.33 |
| Peripheral-vascular | 0.83 | 0.60 | 1.15 |
| Chronic liver | 1.38 | 0.92 | 2.04 |
| COPD | 0.96 | 0.72 | 1.27 |
| Other chronic respiratory | 0.83 | 0.60 | 1.14 |
| Steroid use | 0.78 | 0.51 | 1.16 |
| Regular medication (ref: no) |  |  |  |
| Ace inhibitor: took on day | 0.90 | 0.58 | 1.38 |
| omitted on day | 1.08 | 0.80 | 1.46 |
| not known if took on day | 0.90 | 0.51 | 1.51 |
| Alpha blocker: took on day | 0.94 | 0.40 | 2.00 |
| omitted on day | 0.88 | 0.43 | 1.70 |
| not known if took on day | 0.16 | 0.02 | 0.74 |
| Angio receptor: took on day | 0.91 | 0.55 | 1.46 |
| omitted on day | 1.03 | 0.73 | 1.45 |
| not known if took on day | 0.83 | 0.40 | 1.61 |
| Beta-blocker: took on day | 1.16 | 0.89 | 1.51 |
| omitted on day | 1.73 | 1.20 | 2.46 |
| not known if took on day | 2.33 | 1.44 | 3.72 |
| Calcium blocker: took on day | 0.74 | 0.51 | 1.07 |
| omitted on day | 1.33 | 0.94 | 1.87 |
| not known if took on day | 0.72 | 0.40 | 1.26 |
| Diuretic: took on day | 0.78 | 0.51 | 1.17 |
| omitted on day | 0.91 | 0.65 | 1.25 |
| not known if took on day | 1.42 | 0.82 | 2.40 |
| NSAID: took on day | 0.76 | 0.31 | 1.69 |
| omitted on day | 0.86 | 0.42 | 1.66 |
| not known if took on day | 0.81 | 0.31 | 1.90 |
| Random intercept: standard deviations |  |  |  |
| Country | 0.74 | 0.46 | 1.08 |
| Centre | 0.95 | 0.78 | 1.14 |

## **Table S6: Pre- and intra-operative predictors of PVI use (full model results)**

Estimated odds ratios and random intercept standard deviations from a Bayesian mixed effects logistic regression of postoperative vasopressor infusion use in 19,768 patients (Squeeze Cohort A): Pre- and intra-operative predictors. N = 19768, number of centres: 228; number of countries: 42 Number of events (PVI use): 770 (3.9 %).

Missing values were imputed using multiple imputation with chained equations (mice) with 30 imputations. Bayesian posterior draws were combined to obtain a pooled posterior distribution. The estimates shown are the mean, 2.5th percentile and 97.5th percentile of the posterior distribution.

|  | Odds Ratio | 95 % credible interval | |
| --- | --- | --- | --- |
| Intercept (baseline odds) | 0.00015 | 0.00003 | 0.00061 |
| PRE-OPERATIVE VARIABLES |  |  |  |
| Age (centred, in years) | 1.00875 | 1.00071 | 1.01687 |
| Age(centred)^2^ | 0.99975 | 0.99942 | 1.00007 |
| Frailty (ref: CFS score 1) |  |  |  |
| CFS 2 | 0.98 | 0.64 | 1.53 |
| CFS 3 | 0.76 | 0.49 | 1.20 |
| CFS 4 | 0.83 | 0.52 | 1.37 |
| CFS 5 | 0.77 | 0.44 | 1.36 |
| CFS 6 | 0.79 | 0.43 | 1.47 |
| CFS 7 | 0.85 | 0.46 | 1.60 |
| CFS 8 | 2.02 | 0.86 | 4.66 |
| CFS 9 | 1.95 | 0.45 | 7.55 |
| MAP 12 hrs pre-surgery (ref: ≥ 96mgHG) |  |  |  |
| <90mmHg | 1.19 | 0.93 | 1.53 |
| 90-95.99mmHg | 1.28 | 0.95 | 1.73 |
| MAP immediately pre-surgery (ref: ≥ 96mgHG) |  |  |  |
| <90mmHg | 1.92 | 1.51 | 2.45 |
| 90-95.99mmHg | 0.99 | 0.72 | 1.34 |
| Reason for Surgery (ref: Other) |  |  |  |
| Infection | 1.78 | 1.28 | 2.47 |
| Fracture | 1.13 | 0.86 | 1.50 |
| Cancer | 0.84 | 0.53 | 1.34 |
| Bleeding | 1.05 | 0.67 | 1.63 |
| Surgical Procedure (ref: Orthopaedic) |  |  |  |
| Breast | 0.49 | 0.12 | 1.64 |
| Gynaecological | 1.56 | 0.83 | 2.90 |
| Head and neck | 2.34 | 1.38 | 3.99 |
| Hepato-biliary | 1.70 | 0.98 | 2.95 |
| Kidney/urological | 1.81 | 1.09 | 3.00 |
| Lower gastro-intestinal | 1.73 | 1.11 | 2.71 |
| Plastics/Cutaneous | 1.54 | 0.73 | 3.14 |
| Upper gastro-intestinal | 2.65 | 1.62 | 4.34 |
| Neurological/spinal | 1.73 | 1.04 | 2.88 |
| Vascular | 2.24 | 1.33 | 3.77 |
| Other | 1.96 | 1.03 | 3.69 |
| Severity (ref: Minor) |  |  |  |
| Intermediate | 2.06 | 1.14 | 3.89 |
| Major | 3.37 | 1.81 | 6.52 |
| ASA Grade (ref: ASA 1) |  |  |  |
| ASA2 | 2.41 | 1.45 | 4.07 |
| ASA3 | 3.54 | 2.09 | 6.13 |
| ASA4 | 9.37 | 5.13 | 17.39 |
| ASA5 | 19.65 | 6.86 | 56.66 |
| Urgency (ref: Not urgent) |  |  |  |
| Urgent | 1.84 | 1.43 | 2.38 |
| Medical history |  |  |  |
| Arterial fibrillation | 1.19 | 0.87 | 1.62 |
| Cerebrovascular | 1.41 | 1.04 | 1.90 |
| Coronary Artery | 1.13 | 0.85 | 1.49 |
| Diabetes: insulin dependent | 0.80 | 0.53 | 1.19 |
| Diabetes: non-insulin dependent | 1.06 | 0.80 | 1.40 |
| Heart failure | 1.11 | 0.77 | 1.60 |
| Hypertension | 0.97 | 0.75 | 1.27 |
| Peripheral-vascular | 0.80 | 0.55 | 1.16 |
| Chronic liver | 1.42 | 0.90 | 2.22 |
| COPD | 1.00 | 0.72 | 1.37 |
| Other chronic respiratory | 0.77 | 0.53 | 1.11 |
| Steroid use | 0.94 | 0.59 | 1.46 |
| Regular medication (ref: no) |  |  |  |
| Ace inhibitor: took on day | 0.90 | 0.55 | 1.45 |
| omitted on day | 1.11 | 0.78 | 1.56 |
| not known if took on day | 0.92 | 0.50 | 1.67 |
| Alpha blocker: took on day | 0.88 | 0.35 | 2.06 |
| omitted on day | 0.82 | 0.36 | 1.77 |
| not known if took on day | 0.28 | 0.04 | 1.32 |
| Angio receptor: took on day | 0.86 | 0.49 | 1.46 |
| omitted on day | 0.92 | 0.62 | 1.35 |
| not known if took on day | 0.82 | 0.35 | 1.79 |
| Beta-blocker: took on day | 1.23 | 0.91 | 1.67 |
| omitted on day | 1.80 | 1.19 | 2.70 |
| not known if took on day | 1.91 | 1.11 | 3.25 |
| Calcium blocker: took on day | 0.71 | 0.46 | 1.08 |
| omitted on day | 1.11 | 0.74 | 1.65 |
| not known if took on day | 0.56 | 0.29 | 1.07 |
| Diuretic: took on day | 0.90 | 0.56 | 1.43 |
| omitted on day | 1.03 | 0.71 | 1.48 |
| not known if took on day | 2.20 | 1.20 | 3.97 |
| NSAID: took on day | 0.63 | 0.23 | 1.55 |
| omitted on day | 0.98 | 0.45 | 1.98 |
| not known if took on day | 0.58 | 0.18 | 1.66 |
|  |  |  |  |
| INTRA-OPERATIVE VARIABLES |  |  |  |
| Duration of operation (ref: < 120 mins) |  |  |  |
| 120-239 mins | 1.03 | 0.73 | 1.47 |
| ≥ 240mins | 1.62 | 1.10 | 2.39 |
| Blood loss (ref: 0 – 250 ml) |  |  |  |
| 251-1000ml | 1.44 | 1.13 | 1.85 |
| 1001-3000ml | 2.39 | 1.56 | 3.66 |
| >3000ml | 4.17 | 1.66 | 10.57 |
| Intraoperative MAP (ref: ≥ 96mgHG) |  |  |  |
| <90mmHg | 0.45 | 0.20 | 1.13 |
| 90-95.99mmHg | 0.64 | 0.19 | 2.15 |
| Type of anaesthesia (not mutually exclusive) |  |  |  |
| Volatile | 0.76 | 0.37 | 1.53 |
| TIVA | 0.73 | 0.36 | 1.45 |
| Sedation | 1.38 | 0.71 | 2.65 |
| Regional | 1.16 | 0.78 | 1.72 |
| Spinal | 0.67 | 0.41 | 1.08 |
| Epidural | 3.06 | 2.26 | 4.13 |
| Airway (ref: O2 facemask or nasal canula) |  |  |  |
| Endotracheal tube | 1.58 | 0.71 | 3.61 |
| Supraglottic | 0.63 | 0.23 | 1.67 |
| Vasoactive drugs (not mutually exclusive) |  |  |  |
| Preoperative vasopressors | 5.74 | 3.80 | 8.65 |
| Any intraoperative vasopressors | 7.22 | 5.35 | 9.85 |
| Enteral vasopressors | 6.98 | 3.53 | 13.82 |
| Bolus | 3.57 | 2.59 | 4.90 |
| Crystalloid (ref: None) |  |  |  |
| ≤500ml | 0.84 | 0.43 | 1.69 |
| 501-1500ml | 1.01 | 0.54 | 1.95 |
| >1500ml | 2.44 | 1.30 | 4.73 |
| Colloid (ref: None) |  |  |  |
| ≤500ml | 2.24 | 1.65 | 3.04 |
| >500ml | 2.16 | 1.35 | 3.45 |
| Blood products (ref: None) |  |  |  |
| ≤500ml | 1.57 | 1.08 | 2.26 |
| >500ml | 1.94 | 1.33 | 2.82 |
| Random intercept: standard deviations |  |  |  |
| Country | 0.70 | 0.37 | 1.09 |
| Centre | 0.87 | 0.68 | 1.10 |

## **Table S7: Distributions of outcome measures by Cohort (A or B) and PVI use**

|  |  | **Cohort A (N = 19768)** | | | | | | **Cohort B (N = 5907)** | |
| --- | --- | --- | --- | --- | --- | --- | --- | --- | --- |
| **Outcome measure** | **Category** | **Total** | **(%)** | **No PVI** | **(%)** | **PVI** | **(%)** | **Total** | **(%)** |
| Ventilation | No | 18871 | (95.8) | 18396 | (97.2) | 475 | (61.7) | 2872 | (48.6) |
|  | Yes | 822 | ( 4.2) | 527 | ( 2.8) | 295 | (38.3) | 3032 | (51.4) |
| Myocardial infarction | No | 19629 | (99.7) | 18872 | (99.7) | 757 | (98.3) | 5738 | (97.2) |
|  | Yes | 63 | ( 0.3) | 50 | ( 0.3) | 13 | ( 1.7) | 165 | ( 2.8) |
| Atrial fibrillation | No | 19535 | (99.2) | 18802 | (99.4) | 733 | (95.2) | 5489 | (93.0) |
|  | Yes | 156 | ( 0.8) | 119 | ( 0.6) | 37 | ( 4.8) | 414 | ( 7.0) |
| Other dysrhythmia | No | 19483 | (98.9) | 18766 | (99.2) | 717 | (93.1) | 5523 | (93.6) |
|  | Yes | 207 | ( 1.1) | 154 | ( 0.8) | 53 | ( 6.9) | 380 | ( 6.4) |
| RRT | No | 19484 | (98.9) | 18759 | (99.1) | 725 | (94.2) | 5360 | (90.8) |
|  | Yes | 208 | ( 1.1) | 163 | ( 0.9) | 45 | ( 5.8) | 546 | ( 9.2) |
| Parenteral nutrition | No | 19072 | (96.9) | 18476 | (97.6) | 596 | (77.4) | 4351 | (73.7) |
|  | Yes | 619 | ( 3.1) | 445 | ( 2.4) | 174 | (22.6) | 1554 | (26.3) |
| Antibiotics | No | 17357 | (88.5) | 16891 | (89.6) | 466 | (61.6) | 3206 | (54.8) |
|  | Yes | 2245 | (11.5) | 1954 | (10.4) | 291 | (38.4) | 2649 | (45.2) |
| Complications | No | 14439 | (73.4) | 14219 | (75.2) | 220 | (28.6) | 1572 | (26.7) |
|  | Yes | 5245 | (26.6) | 4695 | (24.8) | 550 | (71.4) | 4320 | (73.3) |
| AKI | No | 7722 | (88.7) | 7290 | (89.9) | 432 | (71.4) | 3255 | (66.4) |
|  | Yes | 988 | (11.3) | 815 | (10.1) | 173 | (28.6) | 1650 | (33.6) |
| 30-day mortality | No | 19250 | (97.9) | 18580 | (98.3) | 670 | (87.5) | 4932 | (84.1) |
|  | Yes | 410 | ( 2.1) | 314 | ( 1.7) | 96 | (12.5) | 929 | (15.9) |
| Length of Stay | Median (IQR) | 3 | (1, 6) | 3 | (1, 6) | 10 | (6, 20) | 12 | (7, 23) |

## **Table S8: Full results of outcome models**

**Table S8a: Ventilation.** Estimates from a Bayesian mixed effects logistic regression of ventilation on PVI and pre-operative predictors of PVI. Complete cases (n = 18257, using Cohorts A and B)

|  | **OR** | **95 % Credible Interval** | |
| --- | --- | --- | --- |
| Intercept | 0.002 | (0.001, | 0.003) |
| PVI | 24.418 | (18.383, | 32.491) |
| CohortB (bias adjustment) | 1.087 | (0.839, | 1.411) |
| Age (centred at 60) | 0.989 | (0.984, | 0.994) |
| Age (centred at 60) squared | 1.000 | (1.000, | 1.000) |
| Frailty: CFS 2 (ref: CFS1) | 0.990 | (0.723, | 1.366) |
| CFS 3 | 1.175 | (0.848, | 1.636) |
| CFS 4 | 1.243 | (0.882, | 1.767) |
| CFS 5 | 1.327 | (0.889, | 1.984) |
| CFS 6 | 1.348 | (0.893, | 2.036) |
| CFS 7 | 1.450 | (0.948, | 2.221) |
| CFS 8 | 2.514 | (1.364, | 4.568) |
| CFS 9 | 1.263 | (0.456, | 3.501) |
| MAP 12hrs prior: <90mmHg (ref: ≥96mmHg) | 0.828 | (0.711, | 0.965) |
| 90-95.99mgHg | 0.828 | (0.692, | 0.987) |
| MAP immed. prior: <90mmHg (ref: ≥96mmHg) | 1.269 | (1.093, | 1.477) |
| 90-95.99mgHg | 1.175 | (0.968, | 1.428) |
| Reason for surgery: Infection (ref: Other) | 1.220 | (1.001, | 1.492) |
| Fracture | 1.004 | (0.832, | 1.216) |
| Cancer | 0.698 | (0.487, | 1.004) |
| Bleeding | 1.759 | (1.328, | 2.315) |
| Surgical procedure: Breast (ref: Orthopaedic) | 1.948 | (0.923, | 3.892) |
| Gynaecological | 1.185 | (0.739, | 1.865) |
| Head and neck | 6.154 | (4.265, | 8.819) |
| Hepato-biliary | 2.739 | (1.888, | 3.967) |
| Kidney/urological | 1.617 | (1.120, | 2.335) |
| Lower gastro-intestinal | 2.519 | (1.843, | 3.448) |
| Plastics/Cutaneous | 2.073 | (1.235, | 3.411) |
| Upper gastro-intestinal | 3.925 | (2.783, | 5.546) |
| Neurological/spinal | 3.679 | (2.493, | 5.463) |
| Vascular | 1.349 | (0.919, | 1.962) |
| Other | 3.043 | (1.971, | 4.622) |
| Operative severity: Intermediate (ref: Minor) | 1.558 | (1.093, | 2.256) |
| Major | 2.949 | (2.054, | 4.286) |
| ASA Grade 2 (ref: ASA 1) | 1.185 | (0.885, | 1.605) |
| ASA 3 | 1.627 | (1.193, | 2.238) |
| ASA 4 | 4.011 | (2.833, | 5.679) |
| ASA 5 | 8.059 | (4.123, | 16.083) |
| Urgency: Urgent (ref: not urgent) | 2.631 | (2.212, | 3.125) |
| Random effect SDs | **SD** |  |  |
| Country | 0.762 | (0.471, | 1.116) |
| Centre | 1.109 | (0.964, | 1.274) |

**Table S8b: Myocardial infarction.** Estimates from a Bayesian mixed effects logistic regression of myocardial infarction on PVI and pre-operative predictors of PVI. Complete cases (n = 18256, using Cohorts A and B)

|  | **OR** | **95 % Credible Interval** | |
| --- | --- | --- | --- |
| Intercept | 0.001 | (0, | 0.003) |
| PVI | 3.924 | (1.680, | 8.634) |
| CohortB (bias adjustment) | 1.602 | (0.783, | 3.571) |
| Age (centred at 60) | 1.016 | (1.002, | 1.032) |
| Age (centred at 60) squared | 1.000 | (0.999, | 1.001) |
| Frailty: CFS 2 (ref: CFS1) | 0.747 | (0.275, | 2.372) |
| CFS 3 | 0.975 | (0.361, | 3.131) |
| CFS 4 | 0.842 | (0.297, | 2.789) |
| CFS 5 | 1.224 | (0.403, | 4.296) |
| CFS 6 | 1.281 | (0.412, | 4.670) |
| CFS 7 | 1.423 | (0.439, | 5.130) |
| CFS 8 | 1.279 | (0.288, | 5.721) |
| CFS 9 | 0.989 | (0.036, | 10.757) |
| MAP 12hrs prior: <90mmHg (ref: ≥96mmHg) | 1.019 | (0.665, | 1.550) |
| 90-95.99mgHg | 1.078 | (0.663, | 1.714) |
| MAP immed. prior: <90mmHg (ref: ≥96mmHg) | 1.009 | (0.670, | 1.52) |
| 90-95.99mgHg | 0.870 | (0.479, | 1.533) |
| Reason for surgery: Infection (ref: Other) | 0.881 | (0.512, | 1.499) |
| Fracture | 0.926 | (0.548, | 1.58) |
| Cancer | 0.502 | (0.178, | 1.359) |
| Bleeding | 1.560 | (0.812, | 2.925) |
| Surgical procedure: Breast (ref: Orthopaedic) | 0.517 | (0.022, | 4.197) |
| Gynaecological | 0.478 | (0.099, | 1.864) |
| Head and neck | 1.025 | (0.344, | 2.82) |
| Hepato-biliary | 1.047 | (0.395, | 2.791) |
| Kidney/urological | 1.422 | (0.606, | 3.391) |
| Lower gastro-intestinal | 0.734 | (0.334, | 1.676) |
| Plastics/Cutaneous | 0.201 | (0.009, | 1.595) |
| Upper gastro-intestinal | 0.847 | (0.350, | 2.105) |
| Neurological/spinal | 0.240 | (0.034, | 1.093) |
| Vascular | 2.019 | (0.893, | 4.691) |
| Other | 1.120 | (0.340, | 3.474) |
| Operative severity: Intermediate (ref: Minor) | 1.544 | (0.607, | 4.562) |
| Major | 1.575 | (0.614, | 4.782) |
| ASA Grade 2 (ref: ASA 1) | 1.573 | (0.526, | 5.795) |
| ASA 3 | 2.885 | (0.944, | 10.988) |
| ASA 4 | 3.676 | (1.131, | 14.295) |
| ASA 5 | 6.182 | (1.436, | 29.522) |
| Urgency: Urgent (ref: not urgent) | 1.155 | (0.716, | 1.867) |
| Random effect SDs | **SD** |  |  |
| Country | 0.460 | (0.029, | 1.019) |
| Centre | 1.025 | (0.726, | 1.361) |

**Table S8c: Atrial fibrillation.** Estimates from a Bayesian mixed effects logistic regression of atrial fibrillation on PVI and pre-operative predictors of PVI. Complete cases (n = 18255, using Cohorts A and B)

|  | **OR** | **95 % Credible Interval** | |
| --- | --- | --- | --- |
| Intercept | 0.001 | (0.000, | 0.002) |
| PVI | 3.910 | (2.319, | 6.431) |
| CohortB (bias adjustment) | 1.440 | (0.912, | 2.340) |
| Age (centred at 60) | 1.052 | (1.039, | 1.065) |
| Age (centred at 60) squared | 1.000 | (0.999, | 1.000) |
| Frailty: CFS 2 (ref: CFS1) | 0.879 | (0.455, | 1.785) |
| CFS 3 | 0.817 | (0.423, | 1.685) |
| CFS 4 | 0.942 | (0.479, | 1.935) |
| CFS 5 | 0.634 | (0.300, | 1.392) |
| CFS 6 | 0.712 | (0.328, | 1.582) |
| CFS 7 | 0.870 | (0.398, | 1.976) |
| CFS 8 | 0.837 | (0.288, | 2.322) |
| CFS 9 | 3.010 | (0.764, | 10.937) |
| MAP 12hrs prior: <90mmHg (ref: ≥96mmHg) | 1.215 | (0.944, | 1.572) |
| 90-95.99mgHg | 1.108 | (0.816, | 1.503) |
| MAP immed. prior: <90mmHg (ref: ≥96mmHg) | 0.744 | (0.573, | 0.968) |
| 90-95.99mgHg | 0.994 | (0.710, | 1.382) |
| Reason for surgery: Infection (ref: Other) | 0.819 | (0.588, | 1.142) |
| Fracture | 0.884 | (0.643, | 1.223) |
| Cancer | 1.023 | (0.533, | 1.940) |
| Bleeding | 0.585 | (0.344, | 0.960) |
| Procedure: Gynaecological (ref: Orthopaedic) | 0.923 | (0.260, | 2.797) |
| Head and neck | 2.724 | (1.278, | 5.658) |
| Hepato-biliary | 1.949 | (0.950, | 4.054) |
| Kidney/urological | 1.990 | (1.001, | 3.957) |
| Lower gastro-intestinal | 3.077 | (1.737, | 5.610) |
| Plastics/Cutaneous | 1.294 | (0.533, | 2.996) |
| Upper gastro-intestinal | 3.553 | (1.680, | 7.396) |
| Neurological/spinal | 0.553 | (0.081, | 2.361) |
| Vascular | 4.230 | (2.302, | 7.965) |
| Other (incl Breast)* | 2.103 | (1.099, | 4.085) |
| Operative severity: Intermediate (ref: Minor) | 1.684 | (0.828, | 3.728) |
| Major | 2.360 | (1.166, | 5.211) |
| ASA Grade 2 (ref: ASA 1) | 1.332 | (0.661, | 2.980) |
| ASA 3 | 1.679 | (0.820, | 3.784) |
| ASA 4 | 2.023 | (0.955, | 4.698) |
| ASA 5 | 2.334 | (0.868, | 6.589) |
| Urgency: Urgent (ref: not urgent) | 1.866 | (1.382, | 2.524) |
| Random effect SDs | **SD** |  |  |
| Country | 0.226 | (0.013, | 0.519) |
| Centre | 0.719 | (0.535, | 0.920) |

*Note: *There was no case of atrial fibrillation observed among patients receiving breast surgery, so breast surgery patients were combined with the category “other surgical procedure” in this analysis.*

**Table S8d: Other dysrhythmia.** Estimates from a Bayesian mixed effects logistic regression of Other dysrhythmia on PVI and pre-operative predictors of PVI. Complete cases (n = 18255, using Cohorts A and B)

|  | **OR** | **95 % Credible Interval** | |
| --- | --- | --- | --- |
| Intercept | 0.001 | (0.000, | 0.003) |
| PVI | 4.869 | (3.174, | 7.435) |
| CohortB (bias adjustment) | 0.728 | (0.498, | 1.083) |
| Age (centred at 60) | 1.008 | (0.999, | 1.017) |
| Age (centred at 60) squared | 1.000 | (1.000, | 1.000) |
| Frailty: CFS 2 (ref: CFS1) | 1.747 | (0.853, | 3.869) |
| CFS 3 | 1.986 | (0.947, | 4.537) |
| CFS 4 | 1.975 | (0.911, | 4.574) |
| CFS 5 | 2.375 | (1.068, | 5.617) |
| CFS 6 | 2.114 | (0.929, | 5.050) |
| CFS 7 | 2.857 | (1.240, | 6.829) |
| CFS 8 | 2.453 | (0.937, | 6.690) |
| CFS 9 | 1.852 | (0.361, | 7.859) |
| MAP 12hrs prior: <90mmHg (ref: ≥96mmHg) | 0.831 | (0.638, | 1.076) |
| 90-95.99mgHg | 0.914 | (0.672, | 1.230) |
| MAP immed. prior: <90mmHg (ref: ≥96mmHg) | 1.069 | (0.822, | 1.399) |
| 90-95.99mgHg | 0.984 | (0.685, | 1.394) |
| Reason for surgery: Infection (ref: Other) | 1.528 | (1.100, | 2.160) |
| Fracture | 1.542 | (1.101, | 2.154) |
| Cancer | 0.957 | (0.545, | 1.676) |
| Bleeding | 1.349 | (0.856, | 2.086) |
| Surgical procedure: Breast (ref: Orthopaedic) | 0.154 | (0.007, | 1.055) |
| Gynaecological | 0.815 | (0.372, | 1.706) |
| Head and neck | 1.452 | (0.801, | 2.623) |
| Hepato-biliary | 1.069 | (0.594, | 1.934) |
| Kidney/urological | 0.789 | (0.439, | 1.398) |
| Lower gastro-intestinal | 0.946 | (0.597, | 1.519) |
| Plastics/Cutaneous | 0.307 | (0.070, | 0.989) |
| Upper gastro-intestinal | 1.294 | (0.771, | 2.184) |
| Neurological/spinal | 0.973 | (0.501, | 1.822) |
| Vascular | 1.150 | (0.654, | 2.009) |
| Other | 1.017 | (0.467, | 2.088) |
| Operative severity: Intermediate (ref: Minor) | 1.597 | (0.873, | 3.132) |
| Major | 2.175 | (1.174, | 4.268) |
| ASA Grade 2 (ref: ASA 1) | 1.153 | (0.625, | 2.255) |
| ASA 3 | 2.027 | (1.082, | 4.017) |
| ASA 4 | 3.186 | (1.603, | 6.524) |
| ASA 5 | 4.458 | (1.815, | 10.779) |
| Urgency: Urgent (ref: not urgent) | 1.493 | (1.112, | 2.019) |
| Random effect SDs | **SD** |  |  |
| Country | 0.256 | (0.011, | 0.632) |
| Centre | 0.912 | (0.722, | 1.122) |

**Table S8e: Renal replacement therapy.** Estimates from a Bayesian mixed effects logistic regression of renal replacement therapy on PVI and pre-operative predictors of PVI. Complete cases (n = 18256, using Cohorts A and B)

|  | **OR** | **95 % Credible Interval** | |
| --- | --- | --- | --- |
| Intercept | 0.001 | (0.000, | 0.002) |
| PVI | 3.101 | (1.892, | 4.965) |
| CohortB (bias adjustment) | 1.525 | (0.985, | 2.441) |
| Age (centred at 60) | 0.984 | (0.977, | 0.992) |
| Age (centred at 60) squared | 1.000 | (0.999, | 1.000) |
| Frailty: CFS 2 (ref: CFS1) | 1.755 | (0.923, | 3.526) |
| CFS 3 | 1.765 | (0.914, | 3.637) |
| CFS 4 | 2.046 | (1.044, | 4.233) |
| CFS 5 | 2.189 | (1.074, | 4.700) |
| CFS 6 | 2.806 | (1.368, | 5.980) |
| CFS 7 | 1.690 | (0.786, | 3.791) |
| CFS 8 | 2.565 | (1.102, | 6.341) |
| CFS 9 | 3.778 | (1.029, | 13.046) |
| MAP 12hrs prior: <90mmHg (ref: ≥96mmHg) | 0.752 | (0.590, | 0.950) |
| 90-95.99mgHg | 0.710 | (0.526, | 0.950) |
| MAP immed. prior: <90mmHg (ref: ≥96mmHg) | 1.594 | (1.254, | 2.034) |
| 90-95.99mgHg | 0.934 | (0.655, | 1.312) |
| Reason for surgery: Infection (ref: Other) | 1.131 | (0.868, | 1.471) |
| Fracture | 0.687 | (0.499, | 0.934) |
| Cancer | 0.449 | (0.226, | 0.865) |
| Bleeding | 0.977 | (0.671, | 1.411) |
| Surgical procedure: Breast (ref: Orthopaedic) | 1.422 | (0.381, | 4.301) |
| Gynaecological | 0.787 | (0.304, | 1.840) |
| Head and neck | 1.350 | (0.711, | 2.569) |
| Hepato-biliary | 1.412 | (0.761, | 2.628) |
| Kidney/urological | 3.162 | (1.875, | 5.327) |
| Lower gastro-intestinal | 1.399 | (0.865, | 2.306) |
| Plastics/Cutaneous | 2.136 | (1.081, | 4.139) |
| Upper gastro-intestinal | 1.750 | (1.025, | 3.030) |
| Neurological/spinal | 0.559 | (0.249, | 1.194) |
| Vascular | 2.351 | (1.394, | 4.043) |
| Other | 1.880 | (0.939, | 3.729) |
| Operative severity: Intermediate (ref: Minor) | 1.055 | (0.684, | 1.644) |
| Major | 1.007 | (0.646, | 1.609) |
| ASA Grade 2 (ref: ASA 1) | 2.117 | (1.103, | 4.298) |
| ASA 3 | 4.961 | (2.564, | 10.000) |
| ASA 4 | 10.695 | (5.405, | 22.089) |
| ASA 5 | 7.968 | (3.239, | 19.978) |
| Urgency: Urgent (ref: not urgent) | 1.601 | (1.217, | 2.110) |
| Random effect SDs | **SD** |  |  |
| Country | 0.275 | (0.014, | 0.703) |
| Centre | 0.921 | (0.725, | 1.127) |

**Table S8f: Parenteral nutrition.** Estimates from a Bayesian mixed effects logistic regression of parenteral nutrition on PVI and pre-operative predictors of PVI. Complete cases (n = 18255, using Cohorts A and B)

|  | **OR** | **95 % Credible Interval** | |
| --- | --- | --- | --- |
| Intercept | 0.001 | (0.000, | 0.002) |
| PVI | 5.423 | (4.068, | 7.210) |
| CohortB (bias adjustment) | 1.071 | (0.822, | 1.402) |
| Age (centred at 60) | 0.994 | (0.989, | 0.999) |
| Age (centred at 60) squared | 1.000 | (0.999, | 1.000) |
| Frailty: CFS 2 (ref: CFS1) | 1.380 | (0.949, | 2.025) |
| CFS 3 | 1.625 | (1.108, | 2.403) |
| CFS 4 | 1.701 | (1.134, | 2.554) |
| CFS 5 | 1.667 | (1.065, | 2.627) |
| CFS 6 | 1.806 | (1.131, | 2.891) |
| CFS 7 | 2.172 | (1.326, | 3.511) |
| CFS 8 | 4.890 | (2.673, | 8.924) |
| CFS 9 | 3.622 | (1.465, | 8.937) |
| MAP 12hrs prior: <90mmHg (ref: ≥96mmHg) | 1.079 | (0.919, | 1.267) |
| 90-95.99mgHg | 0.952 | (0.791, | 1.143) |
| MAP immed. prior: <90mmHg (ref: ≥96mmHg) | 1.238 | (1.049, | 1.453) |
| 90-95.99mgHg | 1.104 | (0.900, | 1.360) |
| Reason for surgery: Infection (ref: Other) | 1.023 | (0.826, | 1.268) |
| Fracture | 1.518 | (1.244, | 1.851) |
| Cancer | 1.076 | (0.649, | 1.780) |
| Bleeding | 0.963 | (0.715, | 1.293) |
| Surgical procedure: Breast (ref: Orthopaedic) | 0.353 | (0.054, | 1.418) |
| Gynaecological | 1.712 | (0.932, | 3.102) |
| Head and neck | 2.708 | (1.608, | 4.576) |
| Hepato-biliary | 7.058 | (4.411, | 11.498) |
| Kidney/urological | 2.077 | (1.277, | 3.435) |
| Lower gastro-intestinal | 9.474 | (6.175, | 14.841) |
| Plastics/Cutaneous | 1.715 | (0.828, | 3.435) |
| Upper gastro-intestinal | 11.715 | (7.519, | 18.702) |
| Neurological/spinal | 1.413 | (0.800, | 2.515) |
| Vascular | 1.702 | (1.033, | 2.866) |
| Other | 1.821 | (0.992, | 3.290) |
| Operative severity: Intermediate (ref: Minor) | 1.171 | (0.800, | 1.748) |
| Major | 2.175 | (1.489, | 3.230) |
| ASA Grade 2 (ref: ASA 1) | 1.402 | (0.969, | 2.049) |
| ASA 3 | 2.307 | (1.575, | 3.423) |
| ASA 4 | 3.037 | (2.007, | 4.651) |
| ASA 5 | 3.033 | (1.645, | 5.631) |
| Urgency: Urgent (ref: not urgent) | 1.869 | (1.532, | 2.274) |
| Random effect SDs | **SD** |  |  |
| Country | 0.564 | (0.304, | 0.884) |
| Centre | 0.913 | (0.770, | 1.074) |

**Table S8g: Antibiotics.** Estimates from a Bayesian mixed effects logistic regression of antibiotics on PVI and pre-operative predictors of PVI. Complete cases (n = 18173, using Cohorts A and B)

|  | **OR** | **95 % Credible Interval** | |
| --- | --- | --- | --- |
| Intercept | 0.017 | (0.012, | 0.024) |
| PVI | 3.396 | (2.739, | 4.197) |
| CohortB (bias adjustment) | 1.078 | (0.874, | 1.335) |
| Age (centred at 60) | 0.998 | (0.995, | 1.002) |
| Age (centred at 60) squared | 1.000 | (1.000, | 1.000) |
| Frailty: CFS 2 (ref: CFS1) | 1.388 | (1.134, | 1.703) |
| CFS 3 | 1.646 | (1.318, | 2.05)0 |
| CFS 4 | 1.758 | (1.391, | 2.225) |
| CFS 5 | 1.826 | (1.393, | 2.397) |
| CFS 6 | 2.477 | (1.877, | 3.263) |
| CFS 7 | 1.929 | (1.440, | 2.592) |
| CFS 8 | 2.839 | (1.848, | 4.400) |
| CFS 9 | 1.720 | (0.785, | 3.736) |
| MAP 12hrs prior: <90mmHg (ref: ≥96mmHg) | 1.169 | (1.052, | 1.299) |
| 90-95.99mgHg | 0.979 | (0.865, | 1.110) |
| MAP immed. prior: <90mmHg (ref: ≥96mmHg) | 1.145 | (1.024, | 1.277) |
| 90-95.99mgHg | 1.016 | (0.887, | 1.162) |
| Reason for surgery: Infection (ref: Other) | 2.539 | (2.215, | 2.909) |
| Fracture | 1.295 | (1.131, | 1.487) |
| Cancer | 1.192 | (0.969, | 1.468) |
| Bleeding | 1.292 | (1.042, | 1.600) |
| Surgical procedure: Breast (ref: Orthopaedic) | 0.377 | (0.205, | 0.650) |
| Gynaecological | 0.660 | (0.493, | 0.885) |
| Head and neck | 0.937 | (0.730, | 1.200) |
| Hepato-biliary | 1.298 | (1.021, | 1.653) |
| Kidney/urological | 1.326 | (1.071, | 1.651) |
| Lower gastro-intestinal | 1.724 | (1.433, | 2.073) |
| Plastics/Cutaneous | 1.141 | (0.849, | 1.528) |
| Upper gastro-intestinal | 1.497 | (1.203, | 1.873) |
| Neurological/spinal | 0.873 | (0.671, | 1.147) |
| Vascular | 0.854 | (0.667, | 1.089) |
| Other | 1.485 | (1.124, | 1.967) |
| Operative severity: Intermediate (ref: Minor) | 1.106 | (0.926, | 1.323) |
| Major | 1.511 | (1.252, | 1.828) |
| ASA Grade 2 (ref: ASA 1) | 1.234 | (1.020, | 1.486) |
| ASA 3 | 1.484 | (1.207, | 1.824) |
| ASA 4 | 2.051 | (1.606, | 2.616) |
| ASA 5 | 1.919 | (1.186, | 3.097) |
| Urgency: Urgent (ref: not urgent) | 2.164 | (1.916, | 2.439) |
| Random effect SDs | **SD** |  |  |
| Country | 0.300 | (0.097, | 0.576) |
| Centre | 0.700 | (0.600, | 0.808) |

**Table S8h: Postoperative complications.** Estimates from a Bayesian mixed effects logistic regression of postoperative complications on PVI and pre-operative predictors of PVI. Complete cases (n = 18244, using Cohorts A and B)

|  | **OR** | **95 % Credible Interval** | |
| --- | --- | --- | --- |
| Intercept | 0.037 | (0.026, | 0.053) |
| PVI | 5.233 | (4.214, | 6.528) |
| CohortB (bias adjustment) | 0.936 | (0.745, | 1.171) |
| Age (centred at 60) | 1.002 | (0.999, | 1.006) |
| Age (centred at 60) squared | 1.000 | (1.000, | 1.000) |
| Frailty: CFS 2 (ref: CFS1) | 1.218 | (1.035, | 1.434) |
| CFS 3 | 1.398 | (1.172, | 1.664) |
| CFS 4 | 1.602 | (1.318, | 1.939) |
| CFS 5 | 1.829 | (1.459, | 2.302) |
| CFS 6 | 2.080 | (1.629, | 2.658) |
| CFS 7 | 1.693 | (1.289, | 2.206) |
| CFS 8 | 1.819 | (1.180, | 2.838) |
| CFS 9 | 2.290 | (1.002, | 5.364) |
| MAP 12hrs prior: <90mmHg (ref: ≥96mmHg) | 1.050 | (0.953, | 1.154) |
| 90-95.99mgHg | 0.989 | (0.888, | 1.099) |
| MAP immed. prior: <90mmHg (ref: ≥96mmHg) | 1.199 | (1.087, | 1.323) |
| 90-95.99mgHg | 1.111 | (0.990, | 1.247) |
| Reason for surgery: Infection (ref: Other) | 1.503 | (1.321, | 1.711) |
| Fracture | 1.285 | (1.147, | 1.445) |
| Cancer | 1.059 | (0.894, | 1.251) |
| Bleeding | 1.476 | (1.202, | 1.811) |
| Surgical procedure: Breast (ref: Orthopaedic) | 0.624 | (0.448, | 0.862) |
| Gynaecological | 0.730 | (0.586, | 0.908) |
| Head and neck | 0.727 | (0.598, | 0.887) |
| Hepato-biliary | 1.052 | (0.848, | 1.297) |
| Kidney/urological | 0.906 | (0.756, | 1.085) |
| Lower gastro-intestinal | 1.306 | (1.110, | 1.533) |
| Plastics/Cutaneous | 1.038 | (0.806, | 1.331) |
| Upper gastro-intestinal | 1.261 | (1.045, | 1.524) |
| Neurological/spinal | 0.759 | (0.614, | 0.937) |
| Vascular | 0.949 | (0.772, | 1.161) |
| Other | 1.146 | (0.893, | 1.471) |
| Operative severity: Intermediate (ref: Minor) | 1.772 | (1.517, | 2.072) |
| Major | 3.363 | (2.850, | 3.988) |
| ASA Grade 2 (ref: ASA 1) | 1.437 | (1.238, | 1.665) |
| ASA 3 | 1.892 | (1.592, | 2.245) |
| ASA 4 | 2.745 | (2.203, | 3.431) |
| ASA 5 | 3.338 | (1.888, | 6.072) |
| Urgency: Urgent (ref: not urgent) | 1.698 | (1.524, | 1.888) |
| Random effect SDs | **SD** |  |  |
| Country | 0.363 | (0.021, | 0.802) |
| Centre | 1.290 | (1.142, | 1.451) |

**Table S8i: Acute Kidney Injury.** Estimates from a Bayesian mixed effects logistic regression of Acute Kidney Injury on PVI and pre-operative predictors of PVI. Complete cases (n = 10247, using Cohorts A and B)

|  | **OR** | **95 % Credible Interval** | |
| --- | --- | --- | --- |
| Intercept | 0.036 | (0.014, | 0.084) |
| PVI | 2.784 | (2.159, | 3.574) |
| CohortB (bias adjustment) | 1.023 | (0.800, | 1.315) |
| Age (centred at 60) | 1.005 | (1.001, | 1.010) |
| Age (centred at 60) squared | 1.000 | (1.000, | 1.000) |
| Frailty: CFS 2 (ref: CFS1) | 1.221 | (0.904, | 1.668) |
| CFS 3 | 1.321 | (0.969, | 1.819) |
| CFS 4 | 1.355 | (0.978, | 1.889) |
| CFS 5 | 1.382 | (0.958, | 1.988) |
| CFS 6 | 1.521 | (1.059, | 2.186) |
| CFS 7 | 1.503 | (1.019, | 2.220) |
| CFS 8 | 1.411 | (0.840, | 2.367) |
| CFS 9 | 1.548 | (0.657, | 3.573) |
| MAP 12hrs prior: <90mmHg (ref: ≥96mmHg) | 1.078 | (0.915, | 1.265) |
| 90-95.99mgHg | 1.158 | (1.016, | 1.322) |
| MAP immed. prior: <90mmHg (ref: ≥96mmHg) | 0.985 | (0.826, | 1.175) |
| 90-95.99mgHg | 0.908 | (0.793, | 1.040) |
| Reason for surgery: Infection (ref: Other) | 0.848 | (0.690, | 1.037) |
| Fracture | 1.042 | (0.769, | 1.396) |
| Cancer | 1.279 | (0.994, | 1.639) |
| Bleeding | 0.853 | (0.715, | 1.015) |
| Surgical procedure: Breast (ref: Orthopaedic) | 0.754 | (0.334, | 1.831) |
| Gynaecological | 0.810 | (0.374, | 1.888) |
| Head and neck | 1.769 | (0.841, | 4.062) |
| Hepato-biliary | 3.551 | (1.707, | 8.056) |
| Kidney/urological | 1.631 | (0.793, | 3.659) |
| Lower gastro-intestinal | 1.031 | (0.488, | 2.35) |
| Plastics/Cutaneous | 1.527 | (0.681, | 3.682) |
| Upper gastro-intestinal | 1.372 | (0.657, | 3.108) |
| Neurological/spinal | 0.626 | (0.281, | 1.518) |
| Vascular | 1.930 | (0.922, | 4.449) |
| Other | 1.515 | (0.699, | 3.543) |
| Operative severity: Intermediate (ref: Minor) | 1.077 | (0.817, | 1.423) |
| Major | 1.426 | (1.079, | 1.898) |
| ASA Grade 2 (ref: ASA 1) | 1.392 | (1.034, | 1.878) |
| ASA 3 | 2.076 | (1.520, | 2.853) |
| ASA 4 | 3.069 | (2.175, | 4.359) |
| ASA 5 | 2.215 | (1.261, | 3.878) |
| Urgency: Urgent (ref: not urgent) | 0.736 | (0.633, | 0.857) |
| Random effect SDs | **SD** |  |  |
| Country | 0.254 | (0.027, | 0.512) |
| Centre | 0.573 | (0.464, | 0.688) |

**Table S8j: 30-day in-hospital mortality.** Estimates from a Bayesian mixed effects logistic regression of 30-day mortality on PVI and pre-operative predictors of PVI. Complete cases (n = 18197, using Cohorts A and B)

|  | **OR** | **95 % Credible Interval** | |
| --- | --- | --- | --- |
| Intercept | 0.005 | (0.003, | 0.011) |
| PVI | 3.819 | (2.681, | 5.425) |
| CohortB (bias adjustment) | 1.024 | (0.733, | 1.431) |
| Age (centred at 60) | 1.013 | (1.007, | 1.019) |
| Age (centred at 60) squared | 1.000 | (1.000, | 1.001) |
| Frailty: CFS 2 (ref: CFS1) | 1.324 | (0.851, | 2.110) |
| CFS 3 | 1.411 | (0.885, | 2.322) |
| CFS 4 | 1.832 | (1.129, | 3.019) |
| CFS 5 | 2.147 | (1.282, | 3.682) |
| CFS 6 | 1.784 | (1.047, | 3.078) |
| CFS 7 | 2.583 | (1.529, | 4.468) |
| CFS 8 | 3.065 | (1.638, | 5.837) |
| CFS 9 | 10.590 | (4.366, | 25.726) |
| MAP 12hrs prior: <90mmHg (ref: ≥96mmHg) | 1.194 | (0.988, | 1.443) |
| 90-95.99mgHg | 1.104 | (0.882, | 1.378) |
| MAP immed. prior: <90mmHg (ref: ≥96mmHg) | 1.312 | (1.081, | 1.590) |
| 90-95.99mgHg | 0.953 | (0.727, | 1.243) |
| Reason for surgery: Infection (ref: Other) | 1.199 | (0.958, | 1.501) |
| Fracture | 1.004 | (0.780, | 1.293) |
| Cancer | 0.718 | (0.475, | 1.083) |
| Bleeding | 1.432 | (1.065, | 1.915) |
| Surgical procedure: Breast (ref: Orthopaedic) | 0.979 | (0.360, | 2.366) |
| Gynaecological | 0.935 | (0.516, | 1.643) |
| Head and neck | 1.069 | (0.661, | 1.713) |
| Hepato-biliary | 1.248 | (0.802, | 1.950) |
| Kidney/urological | 0.791 | (0.509, | 1.228) |
| Lower gastro-intestinal | 1.263 | (0.894, | 1.802) |
| Plastics/Cutaneous | 1.326 | (0.764, | 2.269) |
| Upper gastro-intestinal | 1.496 | (1.022, | 2.212) |
| Neurological/spinal | 0.607 | (0.350, | 1.026) |
| Vascular | 1.021 | (0.670, | 1.563) |
| Other | 0.784 | (0.424, | 1.423) |
| Operative severity: Intermediate (ref: Minor) | 0.871 | (0.617, | 1.236) |
| Major | 0.887 | (0.622, | 1.277) |
| ASA Grade 2 (ref: ASA 1) | 0.806 | (0.544, | 1.207) |
| ASA 3 | 1.054 | (0.702, | 1.616) |
| ASA 4 | 2.586 | (1.681, | 4.035) |
| ASA 5 | 4.811 | (2.679, | 8.702) |
| Urgency: Urgent (ref: not urgent) | 2.576 | (2.049, | 3.224) |
| Random effect SDs | **SD** |  |  |
| Country | 0.692 | (0.476, | 0.967) |
| Centre | 0.403 | (0.251, | 0.554) |

**Table S8k: Length of stay.** Estimates from a Bayesian mixed effects quantile regression of median Length of Stay on PVI and pre-operative predictors of PVI. Complete cases (n = 17169, using Cohorts A and B)

|  | **b** | **95 % Credible Interval** | |
| --- | --- | --- | --- |
| Intercept | 0.918 | (0.363, | 1.497) |
| PVI | 4.453 | (3.829, | 5.090) |
| CohortB (bias adjustment) | 1.965 | (1.277, | 2.633) |
| Age (centred at 60) | 0.006 | (0.001, | 0.012) |
| Age (centred at 60) squared | 0.000 | (0.000, | 0.000) |
| Frailty: CFS 2 (ref: CFS1) | 0.169 | (-0.049, | 0.383) |
| CFS 3 | 0.378 | (0.125, | 0.633) |
| CFS 4 | 0.695 | (0.375, | 1.015) |
| CFS 5 | 1.398 | (0.944, | 1.863) |
| CFS 6 | 1.639 | (1.124, | 2.176) |
| CFS 7 | 1.750 | (1.108, | 2.383) |
| CFS 8 | 2.609 | (1.341, | 4.046) |
| CFS 9 | 3.549 | (0.176, | 7.025) |
| MAP 12hrs prior: <90mmHg (ref: ≥96mmHg) | 0.260 | (0.093, | 0.425) |
| 90-95.99mgHg | 0.091 | (-0.083, | 0.263) |
| MAP immed. prior: <90mmHg (ref: ≥96mmHg) | 0.236 | (0.071, | 0.405) |
| 90-95.99mgHg | 0.067 | (-0.115, | 0.249) |
| Reason for surgery: Infection (ref: Other) | 0.762 | (0.517, | 1.009) |
| Fracture | 0.536 | (0.341, | 0.728) |
| Cancer | 0.451 | (0.183, | 0.722) |
| Bleeding | 0.625 | (0.211, | 1.056) |
| Surgical procedure: Breast (ref: Orthopaedic) | -1.349 | (-1.762, | -0.938) |
| Gynaecological | -0.541 | (-0.847, | -0.241) |
| Head and neck | -0.501 | (-0.773, | -0.224) |
| Hepato-biliary | -0.084 | (-0.426, | 0.275) |
| Kidney/urological | -0.685 | (-0.952, | -0.421) |
| Lower gastro-intestinal | 0.349 | (0.073, | 0.621) |
| Plastics/Cutaneous | 0.116 | (-0.245, | 0.482) |
| Upper gastro-intestinal | 0.251 | (-0.090, | 0.600) |
| Neurological/spinal | 0.045 | (-0.298, | 0.394) |
| Vascular | -0.921 | (-1.313, | -0.525) |
| Other | -0.262 | (-0.688, | 0.176) |
| Operative severity: Intermediate (ref: Minor) | 0.819 | (0.622, | 1.021) |
| Major | 2.498 | (2.262, | 2.731) |
| ASA Grade 2 (ref: ASA 1) | 0.188 | (-0.003, | 0.380) |
| ASA 3 | 0.855 | (0.599, | 1.116) |
| ASA 4 | 3.297 | (2.717, | 3.870) |
| ASA 5 | 5.948 | (3.682, | 8.788) |
| Urgency: Urgent (ref: not urgent) | 1.088 | (0.895, | 1.282) |
| Random effect SDs | **SD** |  |  |
| Country | 1.305 | (0.954, | 1.744) |
| Centre | 0.849 | (0.705, | 1.009) |

## **Table S9: Outcomes by length of vasopressor use (full data for Figure 2)**

**Table S9a: Definitions of the five groups of vasopressor use displayed in Figure 2**

|  | Vasopressors received | | | |
| --- | --- | --- | --- | --- |
| **Group** | Intra-operative vasopressors | Bolus and/or enteral vasopressors | PVI 1-2 days | PVI 3+days |
| **None** | No | No | No | No |
| **Intra-operative vasopressors only** | Yes | No | No | No |
| **Boluses/enteral vasopressors** | (either) | Yes | No | No |
| **PVI 1-2 days** | (either) | (either) | Yes | No |
| **Prolonged PVI** | (either) | (either) | No | Yes |

**Table S9b: Numbers and percentages of the five categories of vasopressor use**

|  | Number | Percent |
| --- | --- | --- |
| None | 9570 | 37.3 % |
| Intra-operative vasopressors only | 8867 | 34.5 % |
| Intraoperative & postoperative boluses/enteral vasopressors | 561 | 2.2 % |
| PVI 1-2 days | 3992 | 15.5 % |
| Prolonged PVI | 2685 | 10.5 % |
| Total | 25675 | 100.0 % |

*Note: These percentages do not constitute estimates of population proportions, since they come from a combined data set using Cohort A and Cohort B.*

**Table S9c: Outcomes of surgery by category of vasopressor use (Cohort A + B combined, n = 25675)**

| **Variable** | **Vasopressor use** | **None** | **(%)** | **Intra-operative only** | **(%)** | **Post-op bolus or enteral** | **(%)** | **PVI 1-2 days** | **(%)** | **Prolonged PVI** | **(%)** |
| --- | --- | --- | --- | --- | --- | --- | --- | --- | --- | --- | --- |
| **Ventilation** | **No** | 9302 | (97.5) | 8598 | (97.4) | 496 | (89.5) | 2510 | (62.9) | 837 | (31.2) |
|  | **Yes** | 237 | (2.5) | 232 | (2.6) | 58 | (10.5) | 1481 | (37.1) | 1846 | (68.8) |
| **Myocardial infarction** | **No** | 9524 | (99.9) | 8801 | (99.7) | 547 | (98.7) | 3915 | (98.1) | 2580 | (96.2) |
|  | **Yes** | 14 | (0.1) | 29 | (0.3) | 7 | (1.3) | 75 | (1.9) | 103 | (3.8) |
| **Arterial fibrillation** | **No** | 9510 | (99.7) | 8744 | (99.0) | 548 | (98.9) | 3808 | (95.4) | 2414 | (90.0) |
|  | **Yes** | 28 | (0.3) | 85 | (1.0) | 6 | (1.1) | 182 | (4.6) | 269 | (10.0) |
| **Dysrhythmia** | **No** | 9494 | (99.5) | 8733 | (98.9) | 539 | (97.3) | 3801 | (95.3) | 2439 | (90.9) |
|  | **Yes** | 44 | (0.5) | 95 | (1.1) | 15 | (2.7) | 189 | (4.7) | 244 | (9.1) |
| **RRT** | **No** | 9464 | (99.2) | 8755 | (99.2) | 540 | (97.5) | 3829 | (95.9) | 2256 | (84.0) |
|  | **Yes** | 75 | (0.8) | 74 | (0.8) | 14 | (2.5) | 162 | (4.1) | 429 | (16.0) |
| **Parenteral nutrition** | **No** | 9392 | (98.5) | 8557 | (96.9) | 527 | (95.1) | 3276 | (82.1) | 1671 | (62.3) |
|  | **Yes** | 147 | (1.5) | 271 | (3.1) | 27 | (4.9) | 715 | (17.9) | 1013 | (37.7) |
| **Antibiotics** | **No** | 8642 | (91.0) | 7779 | (88.4) | 470 | (85.3) | 2583 | (65.3) | 1089 | (41.0) |
|  | **Yes** | 857 | (9.0) | 1016 | (11.6) | 81 | (14.7) | 1371 | (34.7) | 1569 | (59.0) |
| **Any complications** | **No** | 7556 | (79.2) | 6334 | (71.8) | 329 | (59.4) | 1375 | (34.5) | 417 | (15.6) |
|  | **Yes** | 1979 | (20.8) | 2491 | (28.2) | 225 | (40.6) | 2609 | (65.5) | 2261 | (84.4) |
| **AKI** | **No** | 3280 | (92.4) | 3785 | (88.3) | 225 | (83.0) | 2449 | (74.1) | 1238 | (56.1) |
|  | **Yes** | 268 | (7.6) | 501 | (11.7) | 46 | (17.0) | 855 | (25.9) | 968 | (43.9) |
| **Mortality (30 day)*** | **No** | 9394 | (99.5) | 8649 | (99.1) | 537 | (98.9) | 3567 | (96.9) | 2035 | (85.1) |
|  | **Yes** | 43 | (0.5) | 75 | (0.9) | 6 | (1.1) | 114 | (3.1) | 355 | (14.9) |
| **Length of Stay^$^** | **Median (IQR)** | 11 | (8, 18) | 11 | (8, 19) | 11 | (8, 18) | 13 | (9, 22) | 20 | (12, 34) |

*Notes: this descriptive analysis uses all patients (Cohorts A +B, receiving PVI or not, n = 25,675). Sample sizes for some outcomes differ due to missing values in the outcome variables.*

**Mortality (30-day): the sample is those who survived beyond the period during which PVI use was assessed (at least 7 days), N = 24775.*

*^$^Length of stay: the sample consists of patients who stayed in hospital beyond the period during which PVI use was assessed (at least 7 days), N = 8353.*

## **Table S10** **Type of vasoactive infusion given by day post-surgery (full data for Figure 3)**

| Infusion | Day^$^ | Number | Percent |  | Infusion | Day^$^ | Number | Percent |
| --- | --- | --- | --- | --- | --- | --- | --- | --- |
| Noradrenaline | 0 | 5292 | 79.3% |  | Phenylephrine | 0 | 329 | 4.9% |
|  | 1 | 4034 | 60.4% |  |  | 1 | 106 | 1.6% |
|  | 2 | 2251 | 33.7% |  |  | 2 | 29 | 0.4% |
|  | 3 | 1299 | 19.5% |  |  | 3 | 12 | 0.2% |
|  | 4 | 840 | 12.6% |  |  | 4 | 7 | 0.1% |
|  | 5 | 616 | 9.2% |  |  | 5 | 7 | 0.1% |
|  | 6 | 479 | 7.2% |  |  | 6 | 7 | 0.1% |
| Angiotensin | 0 | 6 | 0.1% |  | Terlipressin | 0 | 21 | 0.3% |
|  | 1 | 7 | 0.1% |  |  | 1 | 21 | 0.3% |
|  | 2 | 8 | 0.1% |  |  | 2 | 11 | 0.2% |
|  | 3 | 5 | 0.1% |  |  | 3 | 8 | 0.1% |
|  | 4 | 6 | 0.1% |  |  | 4 | 6 | 0.1% |
|  | 5 | 5 | 0.1% |  |  | 5 | 2 | 0.0% |
|  | 6 | 3 | 0.0% |  |  | 6 | 0 | 0.0% |
| Dopamine | 0 | 144 | 2.2% |  | Vasopressin | 0 | 300 | 4.5% |
|  | 1 | 100 | 1.5% |  |  | 1 | 297 | 4.4% |
|  | 2 | 70 | 1.0% |  |  | 2 | 170 | 2.5% |
|  | 3 | 59 | 0.9% |  |  | 3 | 89 | 1.3% |
|  | 4 | 55 | 0.8% |  |  | 4 | 53 | 0.8% |
|  | 5 | 29 | 0.4% |  |  | 5 | 38 | 0.6% |
|  | 6 | 23 | 0.3% |  |  | 6 | 23 | 0.3% |
| Ephinephrine | 0 | 258 | 3.9% |  | Dobutamine* | 0 | 148 | 2.2% |
|  | 1 | 195 | 2.9% |  |  | 1 | 165 | 2.5% |
|  | 2 | 104 | 1.6% |  |  | 2 | 122 | 1.8% |
|  | 3 | 63 | 0.9% |  |  | 3 | 92 | 1.4% |
|  | 4 | 49 | 0.7% |  |  | 4 | 45 | 0.7% |
|  | 5 | 34 | 0.5% |  |  | 5 | 33 | 0.5% |
|  | 6 | 25 | 0.4% |  |  | 6 | 23 | 0.3% |
| Metaraminol | 0 | 859 | 12.9% |  | Milrinone* | 0 | 5 | 0.1% |
|  | 1 | 580 | 8.7% |  |  | 1 | 8 | 0.1% |
|  | 2 | 259 | 3.9% |  |  | 2 | 5 | 0.1% |
|  | 3 | 91 | 1.4% |  |  | 3 | 5 | 0.1% |
|  | 4 | 34 | 0.5% |  |  | 4 | 4 | 0.1% |
|  | 5 | 17 | 0.3% |  |  | 5 | 4 | 0.1% |
|  | 6 | 7 | 0.1% |  |  | 6 | 4 | 0.1% |

Notes: Percentages are calculated relative to the total number of patients in the sample who received postoperative vasopressor infusions (n = 6,677). *The iodilators dobutamine and milrinone were not counted as vasopressors. They are included here to document their frequency of use among patients who also received post-operative infusions with vasopressors (PVI). Patients who did not receive PVI may have received iodilators, but are not included in this table.. $Day: Calendar day after surgery (0 = day of surgery)

## **Table S11: Assessment of postoperative vasopressor infusion need**

Question 6.5 in CRF 2 asked the question: “How was it assessed that this patient should receive a vasopressor infusion?” This question was only asked about patients who did receive postoperative vasopressor infusions (according to the Squeeze definition). The responses in all Squeeze data are summarized in the table below (Cohort A + Cohort B)

| **Assessment of PVI need** | **Count** | **Percentage (%)** |
| --- | --- | --- |
| Already receiving | 4313 | 64.6 |
| Fluids not working: Cardiac output monitoring | 413 | 6.2 |
| Fluids not working: Clinical assessment alone | 1227 | 18.4 |
| Fluids not working: Echocardiography | 129 | 1.9 |
| Fluids not working: Other | 40 | 0.6 |
| Fluids not working: Unknown | 52 | 0.8 |
| Fluids not working: maximum fluids met | 500 | 7.5 |
| Missing | 3 | 0.0 |
| Total | 6677 | 100.0 |

## **Table S12: PVI use by MAP target**

|  | No MAP target recorded | | All MAP targets ≤ 65 | | At least one MAP target > 65 | | Total | |
| --- | --- | --- | --- | --- | --- | --- | --- | --- |
| 1-2 days PVI use | 1548 | (64.3) | 1444 | (60.5) | 1000 | (53.1) | 3992 | (59.8) |
| 3 or more days PVI use | 858 | (35.7) | 944 | (39.5) | 883 | (46.9) | 2685 | (40.2) |
| Total | 2406 | (100.0) | 2388 | (100.0) | 1883 | (100.0) | 6677 | (100.0) |

Prolonged PVI use by MAP targets (Squeeze recipients of PVI only). Number (percentages in brackets)
